# Supplementary material for: A robust platform streamlining aromatic noncanonical amino acid biosynthesis and genetic code expansion in Escherichia coli
Source: Nat Commun. 2025 Sep 29;16:8605. doi: 10.1038/s41467-025-63679-6 (PMC12480666; doi:10.1038/s41467-025-63679-6)
Supplement: Supplementary file 1 — Supplementary Information [file 41467_2025_63679_MOESM1_ESM.pdf]

A robust platform streamlining aromatic noncanonical amino acid biosynthesis and genetic code expansion in *Escherichia coli*

Jingxuan Zhang,[1,2#] Keying Yu,[3, 1#] Yali Xu,[4#] Wushuang Zhao,[1,5] Yulian Li,[1,5] Ying Wang[2], Florian P. Seebeck,[6] Xiao-Hua Chen,[4,7*] and Cangsong Liao[1,3,5*]

[1]State Key Laboratory of Chemical Biology, Shanghai Institute of Material Medica, Chinese Academy of Sciences, Shanghai 201203, China.
[2]State Key Laboratory of Synthetic Biology, Tianjin University, Tianjin 300072, China
[3]School of Chinese Materia Medica, Nanjing University of Chinese Medicine, Nanjing 210023, China
[4]State Key Laboratory of Drug Research, Shanghai Institute of Materia Medica, Chinese Academy of Sciences, Shanghai 201203, China
[5]University of Chinese Academy of Sciences, Beijing 100049, China
[6]Department for Chemistry, University of Basel, Basel, Switzerland
[7]School of Pharmaceutical Science and Technology, Hangzhou Institute for Advanced Study, University of Chinese Academy of Sciences, Hangzhou 310024, China

[#]These authors contributed equally to this work.

*To whom correspondence should be addressed: Cangsong Liao: csliao@simm.ac.cn
Xiao-Hua Chen: xhchen@simm.ac.cn

Contents

Supplementary Figures and Tables 3

Supplementary Methods 31

Supplementary method 1. General information 31

Supplementary method 2. Synthesis and characterization of ncAA products 33

Supplementary method 3. Substrate scope of aromatic ncAAs biosynthesis in culture. 48

Supplementary method 4. Expression, fluorescence measurement and purification of sfGFP 49

Supplementary method 5. Macrocyclic peptide biosynthesis and analysis. 50

Supplementary method 6. Expression and purification of pAzF-containing antibody fragments. 51

Supplementary method 7. Protein conjugation 52

Supplementary method 8. Confocal imaging 52

NMR spectra 53

High resolution mass spectra 70

Peptide macrocycles mass spectra 78

Supplementary Figures and Tables

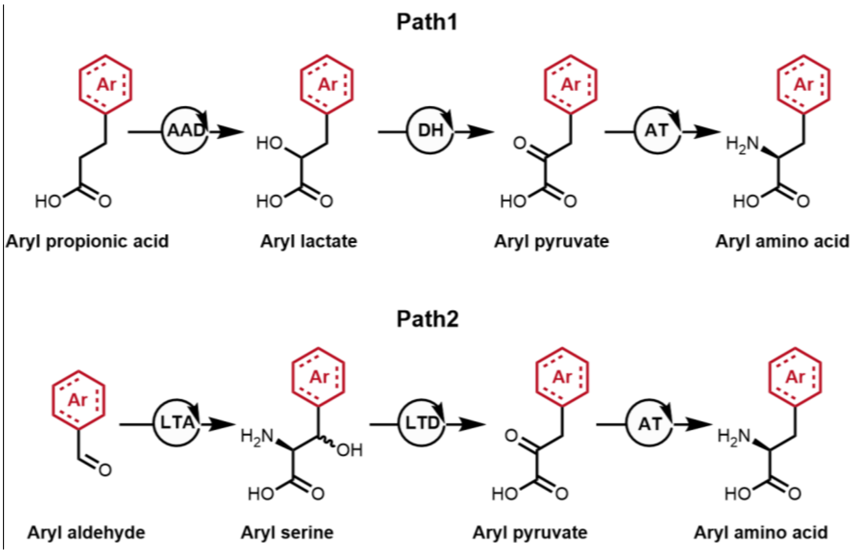

Supplementary Figure 1 Two biosynthetic pathways were designed for the synthesis of ncAAs *in vivo*. a. Aryl propionic acid was used as starting materials and dioxygenase (AAD) catalyzed the Cα-H oxyfunctionalization to give Aryl lactate, which was then oxidized by dehydrogenase (DH) to aryl pyruvate and then converted to aryl amino acid by aminotransferase (AT). **b.** Aryl aldehyde was used as starting materials and the first reaction is an aldol reaction with glycine by L-threonine aldolase (LTA) to produce aryl serine. The intermediate was then transformed to aryl pyruvate by L-threonine deaminase (LTD) and converted to aryl amino acid by aminotransferase (AT).

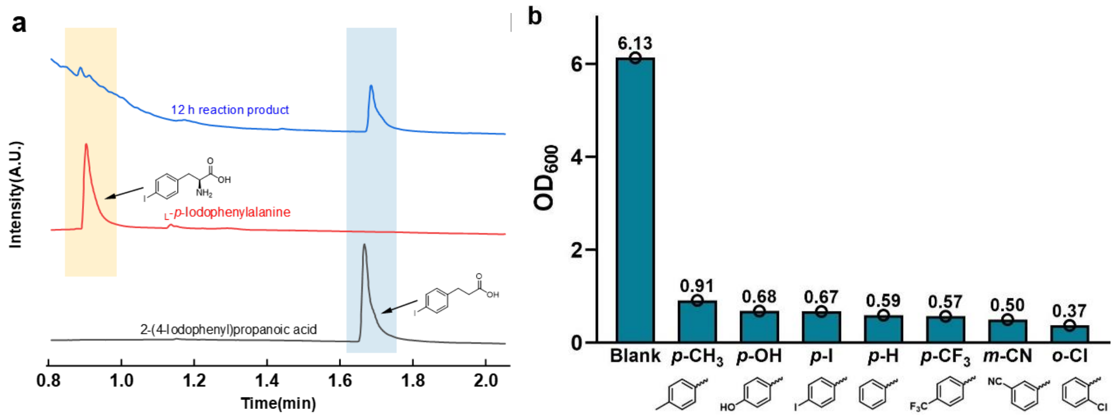

Supplementary Figure 2 The transformation and impact of 2-(4-Iodophenyl)propanoic acid in cell culture. a. The conversion of 2-(4-Iodophenyl)propanoic acid in clutrue as a representative. **b**. OD_{600} of *E. coli* BL21 (SdpA-PaHADH) after the addition of the reaction mixtures with phenylpropanoic acid derivatives after 12 hours. 1 mM 2-(4-Iodophenyl)propanoic acid, 2 mM ascorbic acid, 4 mM *α*-ketoglutarate and 100 μM $FeSO_4$ were added to the culture medium of *E. coli* BL21 (DE3) harboring dioxygenase SdpA and hydroxy acid dehydrogenase PaHADH at the same time of IPTG inducing expression when OD_{600} reached 1.0. The strains were then induced at 30 °C for 12 h, and samples were taken every two hours to detect bacterial concentration (OD_{600}). The conversion of 2-(4-Iodophenyl)propanoic acid was monitored by UPLC-MS after 12 hours.

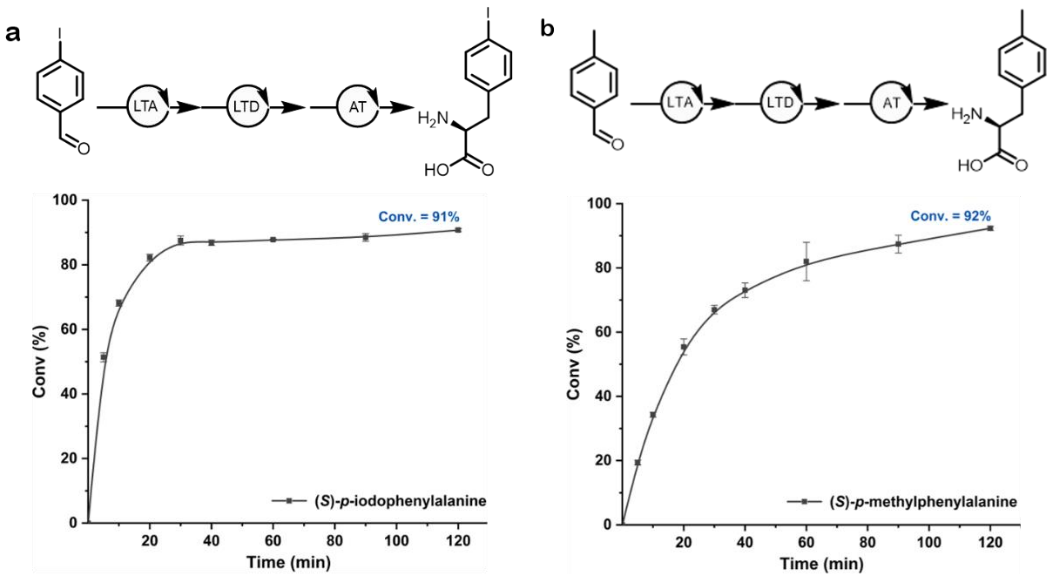

Supplementary Figure 3 Cascade reaction for synthesis of amino acids from aromatic aldehydes by purified enzyme. Conversion of reaction using *p*-iodobenzaldehyde **(a)** or *p*-methylbenzaldehyde **(b)** as substrate were monitored by UPLC-MS with standard curve of the products. 1 mL mixture contained 1 mM aromatic aldehydes (10% DMSO), 5 mM glycine (Gly), 5 mM L-glutamic acid (L-Glu), 0.1 mg·mL^{-1} PpLTA, 0.1 mg·mL^{-1} RpTD and 0.1 mg·mL^{-1} TyrB in phosphate buffer (50 mM Na_2HPO_4, pH 7.5, 50 mM NaCl). The mixture was stirred at room temperature (25 °C). Error bars represent the mean ± s.d. of n = 3 independent samples. Source data of **a** and **b** are provided in Source Data file.

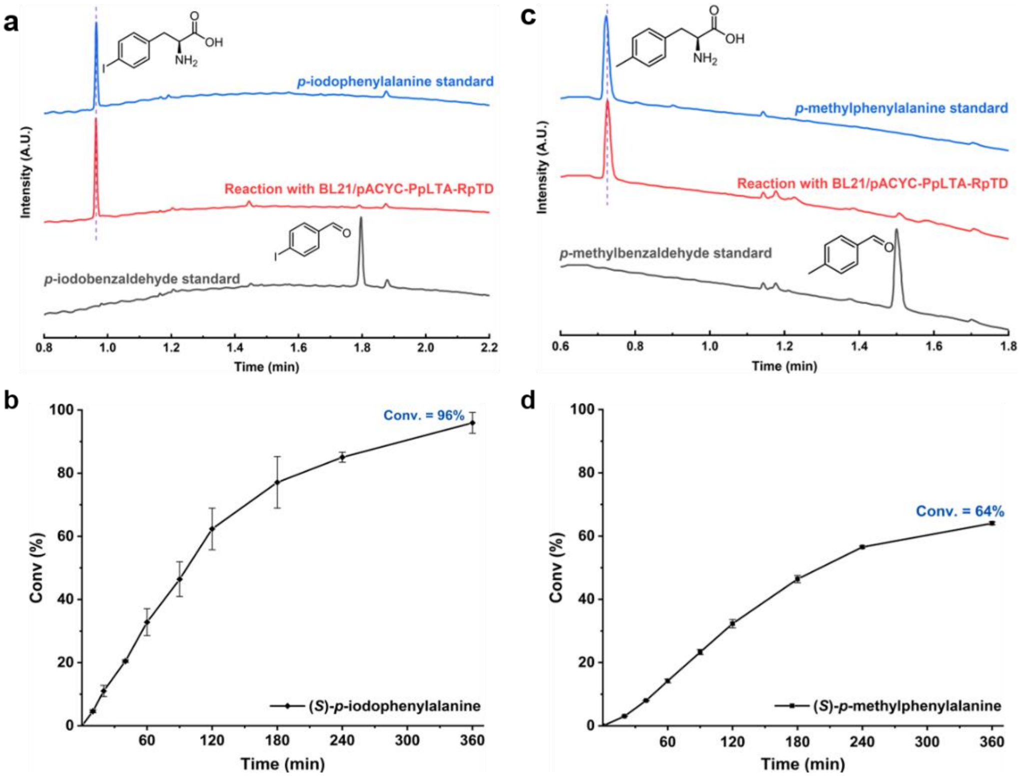

Supplementary Figure 4 Synthesis of amino acids from aldehydes by lyophilized *E. coli* BL21 (PpLTA-RpTD) whole-cell catalysis. UPLC-UV chromatogram for qualitative analysis of the reaction using *p*-iodobenzaldehyde **(a)** or *p*-methylbenzaldehyde **(b)**. Conversion of reaction using *p*-iodobenzaldehyde **(c)** or *p*-methylbenzaldehyde **(d)** as substrate were monitored by UPLC-MS with standard curve of the products. 1 mL mixture contained 1 mM aromatic aldehydes (10% DMSO), 5 mM Gly, 5 mM L-Glu and 5 mg lyophilized cells in phosphate buffer (50 mM Na_2HPO_4, pH 7.4, 50 mM NaCl). The mixture was stirred at room temperature (25 °C) and analyzed by UPLC-MS. Error bars represent the mean ± s.d. of n = 2 independent samples. Source data of **b** and **d** are provided in Source Data file.

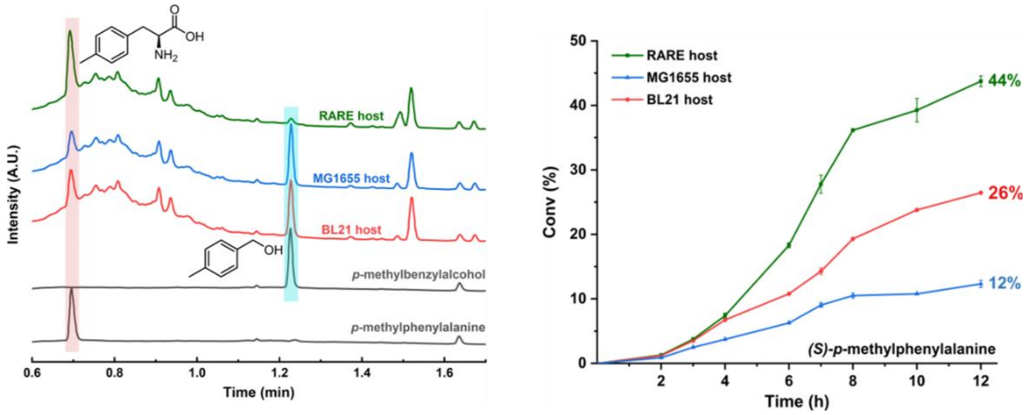

Supplementary Figure 5 Synthesis of amino acids from aldehydes in cultures of *E. coli* host expression PpLTA and RpTD. Products and by-products were detected by UPLC-MS. The conversion of *p*-methylphenylalanine from *p*-methylbenzaldehyde monitored with standard curve of the product. The reaction was carried out in a 50 mL shaking flasks. *p*-methylbenzaldehyde and Gly with a final concentration of 1 mM and 50 mM were added to the medium at the same time of inducing expression, and the final concentration of 20 μM pyridoxal 5′-phosphate (PLP) was added to promote the reaction. Error bars represent the mean ± s.d. of n = 3 independent samples. Source data are provided in Source Data file.

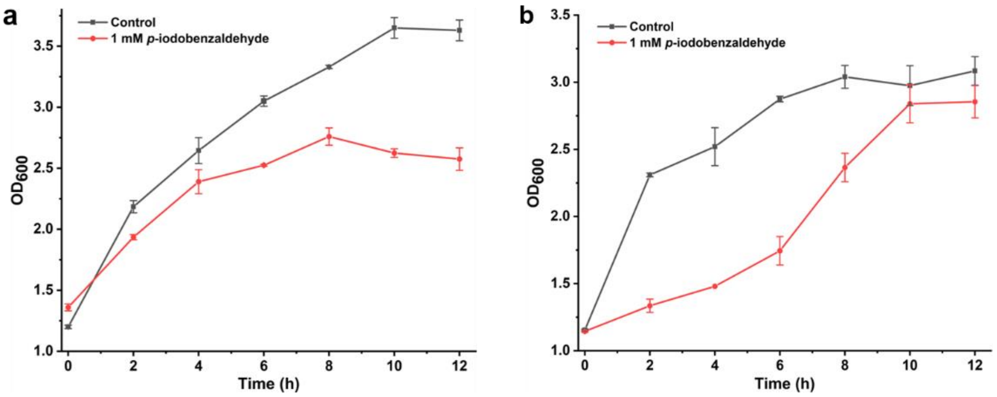

Supplementary Figure 6 The impact of *p*-iodobenzaldehyde on cell growth of *E. coli*. a. *E. coli* BL21 (DE3) harboring PpLTA and RpTD. **b.** *E. coli* RARE (DE3) harboring PpLTA and RpTD.1 mM *p*-iodobenzaldehyde, 50 mM Gly and 20 μM PLP were added to the culture medium at the same time of IPTG inducing expression when OD_{600} reached 1.0. No reactive components were added to the control group. The strains were then induced at 30 °C for 12 h, and samples were taken every two hours to detect bacterial concentration (OD_{600}). Error bars represent the mean ± s.d. of n = 2 independent samples. Source data of **a** and **b** are provided in Source Data file.

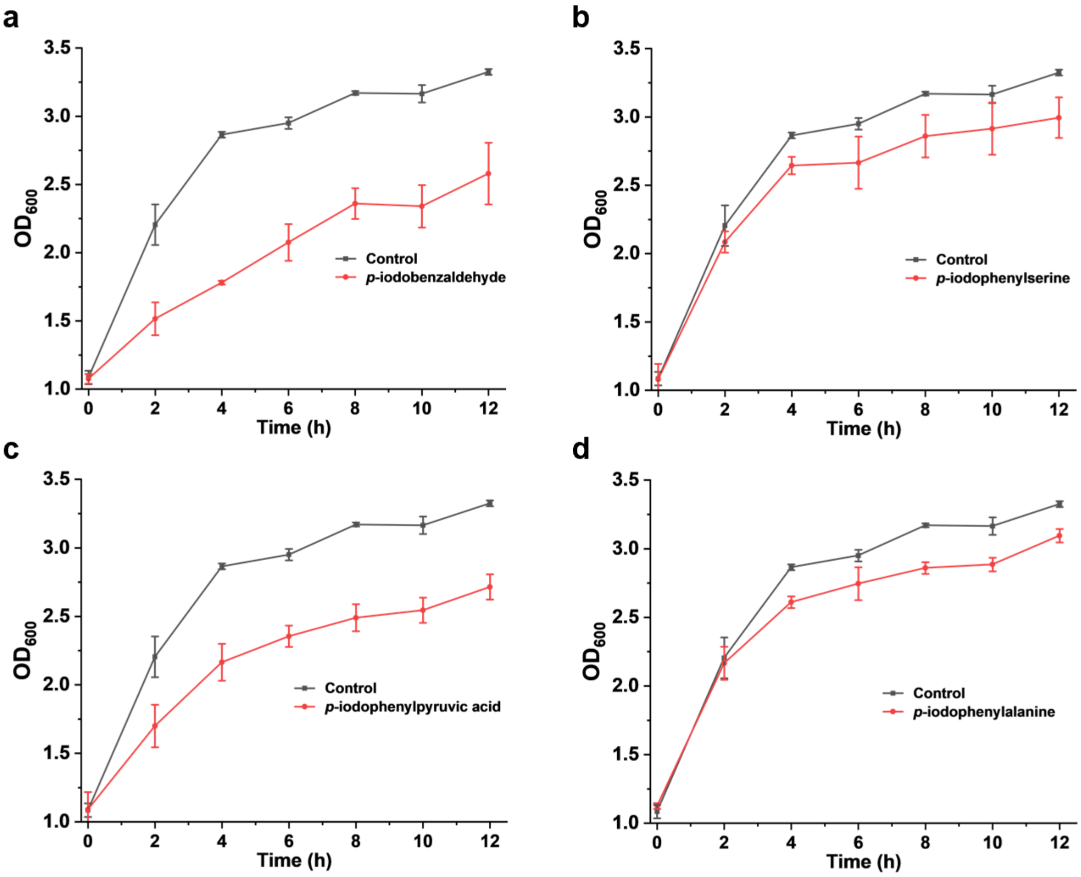

Supplementary Figure 7 The impact of intermediates of aryl-aldehydes to ncAAs on cell growth of *E. coli*. *E. coli* RARE (DE3) harboring PpLTA and RpTD. 1 mM *p*-iodobenzaldehyde **(a)**, 1 mM *p*-iodophenylserine **(b)**, 1 mM *p*-iodophenylpyruvic acid **(c)** or 1 mM *p*-iodophenylalanine **(d)** were added to the culture medium at the same time of IPTG inducing expression when OD_{600} reached 1.0. No reactive components were added to the control group. The strains were then induced at 30 °C for 12 h, and samples were taken every two hours to detect bacterial concentration (OD_{600}). Error bars represent the mean ± s.d. of n = 2 independent samples. Source data of all figures are provided in Source Data file.

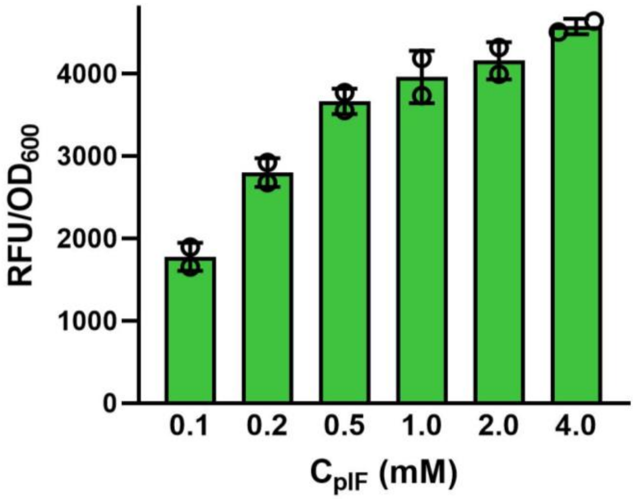

Supplementary Figure 8 GFP production level was related to ncAA concentration. Detection of sfGFP(Y151pIF) fluorescence intensity of strain expressed PpLTA-RpTD, pIFRS/tRNA$^{Pyl}_{CUA}$ pair and sfGFP(Y151TAG). Different *p*-iodobenzaldehyde concentration were added in the culture and induced at 30℃ for 24 h. The density of cells and the fluorescence intensity (Ex: 485 nm; Em: 528 nm) of sfGFP with incorporation of biosynthetic pIF was detected by a plate reader. Error bars represent the mean ± s.d. of n = 2 independent samples. Source data are provided in Source Data file.

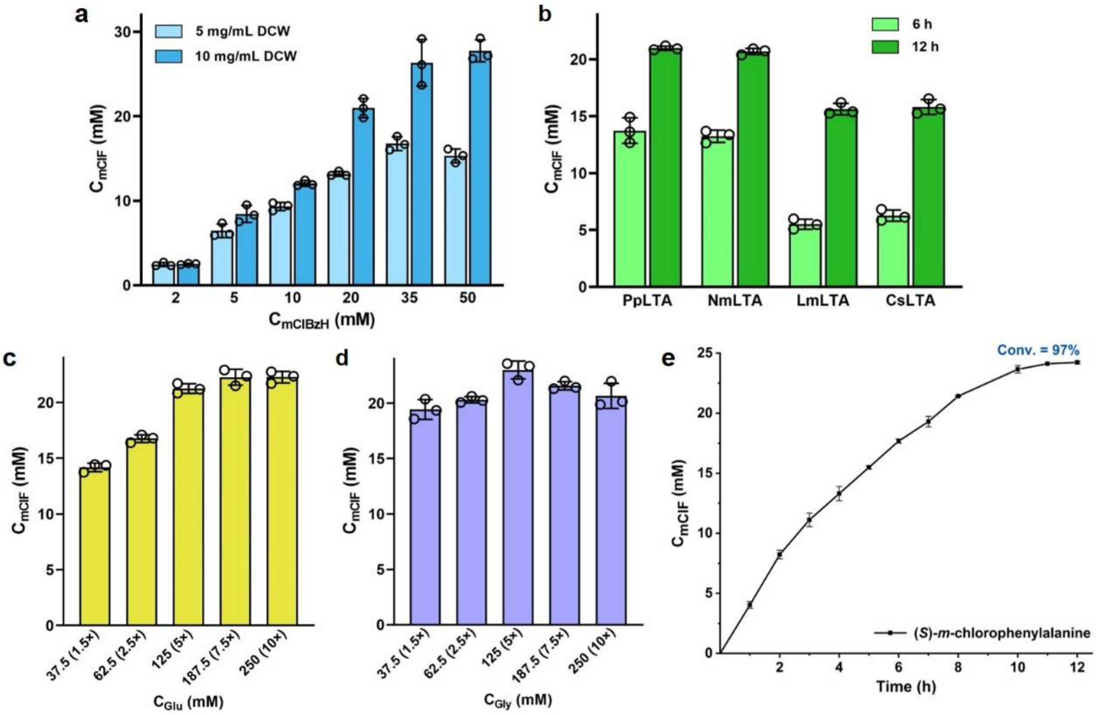

Supplementary Figure 9 Optimization of *E. coli* whole-cell catalysis conditions with *m*-Chlorobenzaldehyde as substrate. a. Optimization of substrate concentration and catalyst loading. 5 mg·mL^{-1} and 10 mg·mL^{-1} lyophilized cells were used as catalysts under different concentration gradients. 25 mM substrate concentration and 10 mg·mL^{-1} catalyst were selected for reaction. **b.** Comparison of catalytic efficiency of different LTAs. PpLTA was used for whole-cell cascade reaction. **c.** Optimization of L-Glu concentration. 5 equivalent of substrates showed the best reaction efficiency. **d.** Optimization of Gly concentration. 5 equivalent of substrates showed the best reaction efficiency. **e.** The conversion of mClF from *m*-Chlorobenzaldehyde under optimal condition was monitored with standard curve of the product: 25 mM substrates, 187.5 mM L-Glu, 125 mM Gly and 10 mg·mL^{-1} lyophilized cell *E. coli* BL21 (DE3)/pACYC-PpLTA-RpTD in phosphate buffer (50 mM Na_2HPO_4, pH 7.4, 50 mM NaCl) with 10% DMSO. The conversion detected by UPLC-MS was 97%. All the error bars represent the mean ± s.d. of n = 3 independent samples. mClBzH, *m*-chlorobenzaldehyde; mClF, *m*-chlorophenylalanine. Source data of all figures are provided in Source Data file.

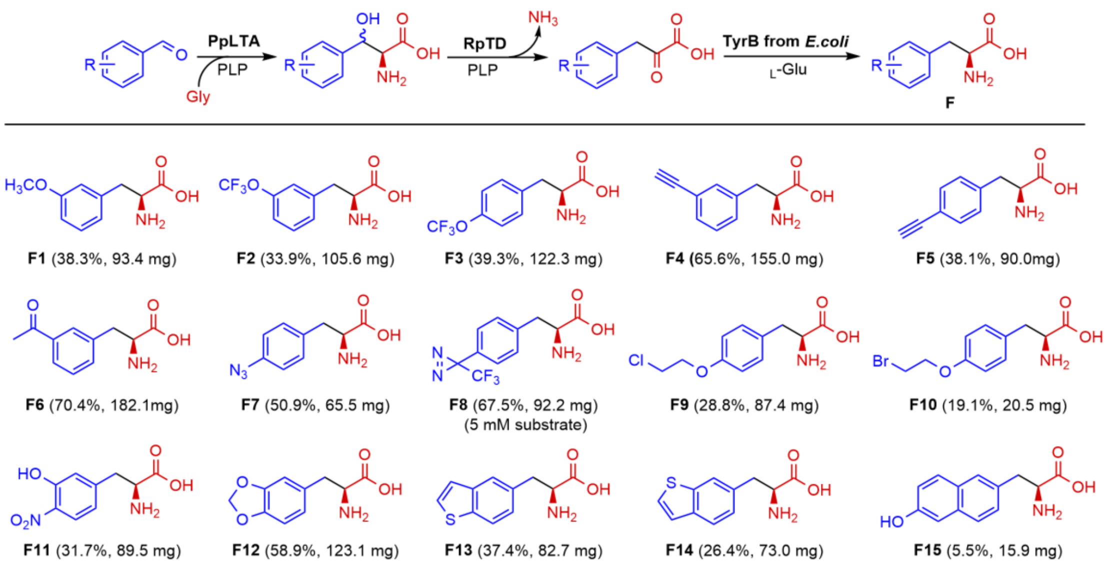

Supplementary Figure 10 Synthesis of Aromatic ncAAs by lyophilized *E. coli* (PpLTA-RpTD) whole-cell catalyst. 50 mL reaction mixtures contained 25 mM Aromatic aldehydes except 5 mM of substrate for **F8**, 125 mM Gly and 187.5 mM L-Glu (F8 group maintained the same proportion), 0.5 g lyophilized cell *E. coli* BL21 (DE3)/pACYC-PpLTA-RpTD in phosphate buffer (50 mM Na_2HPO_4, pH 7.4, 50 mM NaCl) with 10% DMSO. The mixture was stirred at room temperature (25 °C) for 12 h and products were purified and characterized by HR-MS and NMR spectroscopy.

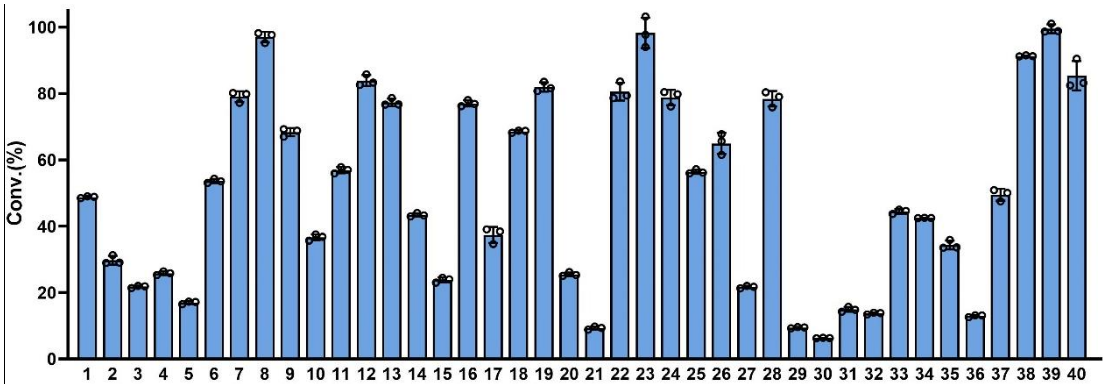

Supplementary Figure 11 Biosynthetic efficiency of ncAAs from aromatic aldehyde precursors by lyophilized *E. coli* RARE (CsLTA-RpTD) whole-cell catalyst. In whole-cell catalysis, 1mL reaction mixtures contained 1 mM aromatic aldehydes, 10 mM Gly, 10 mM L-Glu and 10 mg lyophilized cell *E. coli* RARE/pACYC-CsLTA-RpTD in phosphate buffer (50 mM Na_2HPO_4, pH 7.4, 50 mM NaCl) with 10% DMSO. The mixture was stirred at room temperature (25 °C) for 12 h and products were characterized by UPLC-MS. Error bars represent the mean ± s.d. of n = 3 independent samples. Source data are provided in Source Data file.

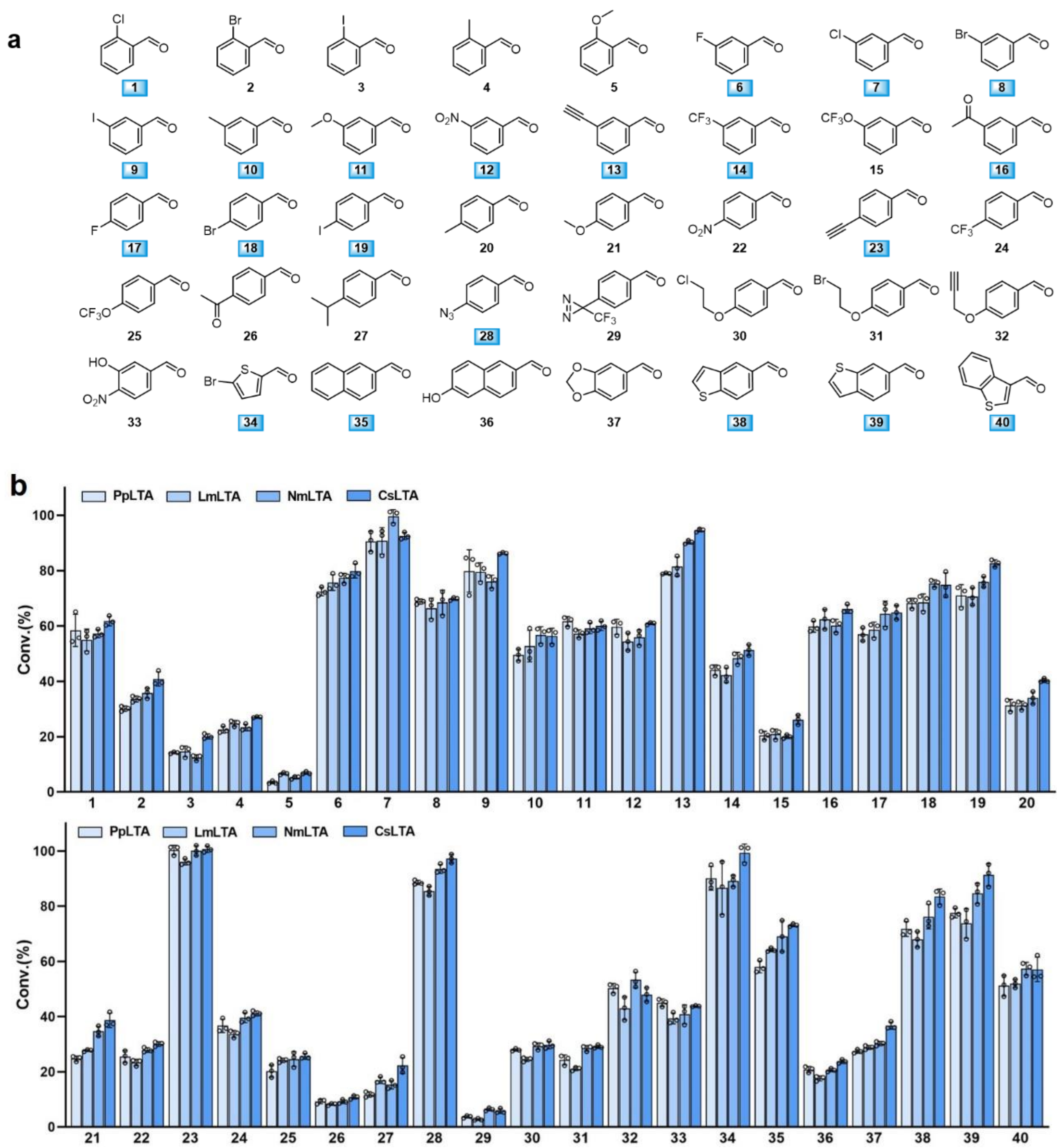

Supplementary Figure 12 Biosynthetic efficiency of ncAAs in medium from aromatic aldehyde precursors with *E. coli* varying LTAs. a. Various structures of aromatic aldehyde precursors. Substrates with a conversion rate of more than 50% were identified in blue boxes. **b.** Four LTA variants were screened for efficiency and scope of ncAA biosynthesis. Reaction was carried out in 96-deep well plates. Aromatic aldehydes, Gly and PLP with a final concentration of 1 mM, 50 mM and 20 μM were added to the medium at the same time with expression induction. The products were detected and the conversions were calculated after reaction at 30 ℃ for 12 h. Error bars represent the mean ± s.d. of n = 3 independent samples. Source data of **b** are provided in Source Data file.

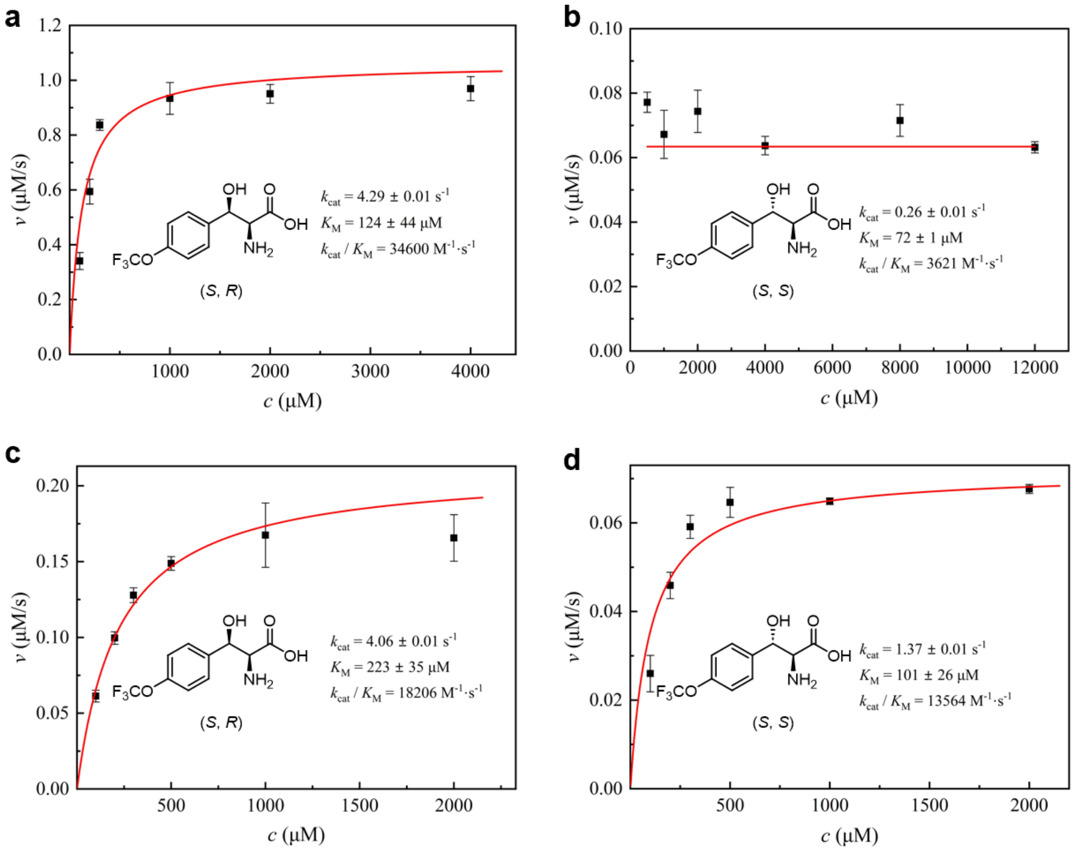

Supplementary figure 13 enzyme kinetics for the aldol reaction in ncAA biosynthesis. Kinetic analysis of PpLTA or CsLTA for *p*-(Trifluoromethoxy)benzaldehyde were monitored by UPLC-MS with standard curve of the product. **a**. For the (*2S, 3R*)-product of PpLTA, the substrate was dissolved in DMSO and diluted to 0.1 mM, 0.2 mM, 0.3 mM, 1 mM, 2 mM, 4 mM. **b**. For the (*2S, 3S*)-product of PpLTA, the substrate was dissolved in DMSO and diluted to 0.5 mM, 1 mM, 2 mM, 4 mM, 8 mM, 12 mM. For the (*2S, 3R*)-product (**c**) of CsLTA or (*2S, 3S*)-product (**d**) of CsLTA, the substrate was dissolved in DMSO and diluted to 0.1 mM, 0.2 mM, 0.3 mM, 0.5 mM, 1 mM, 2 mM. The concentration of the substrate glycine was always saturated solution. 10 μg·mL^{-1} PpLTA or 2 μg·mL^{-1} CsLTA in phosphate buffer (50 mM Na_2HPO_4, pH 7.5, 50 mM NaCl). The mixture was stirred at room temperature (25 °C). Sample of the reaction solution were taken every 30 s for analysis. Error bars represent the mean ± s.d. of n = 3 independent samples. Source data of all figures are provided in Source Data file.

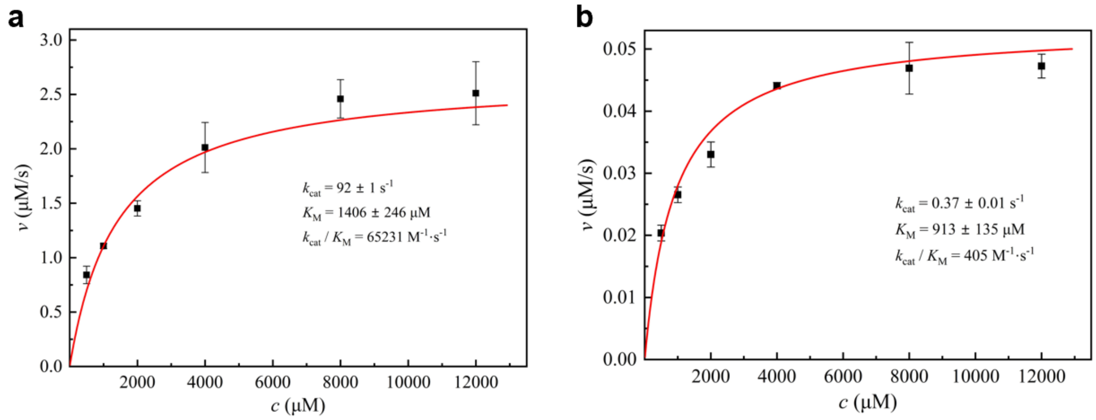

Supplementary figure 14 enzyme kinetics for the deamination in ncAA biosynthesis. Kinetic analysis of RpTD for *p*-(Trifluoromethoxy)phenylserine were monitored by UPLC-MS with standard curve of the product. Substrate was dissolved in phosphate buffer and diluted to 0.5 mM, 1 mM, 2 mM, 4 mM, 8 mM, 12 mM. For (*2S, 3R*)-substrate **(a)** added 1 μg·mL^{-1} and for (*2S, 3S*)-substrate **(b)** added 5 μg·mL^{-1} RpTD in phosphate buffer (50 mM Na_2HPO_4, pH 7.5, 50 mM NaCl). The mixture was stirred at room temperature (25 °C). Sample of the reaction solution were taken every 30 s for analysis. Error bars represent the mean ± s.d. of n = 3 independent samples. Source data of **a** and **b** are provided in Source Data file.

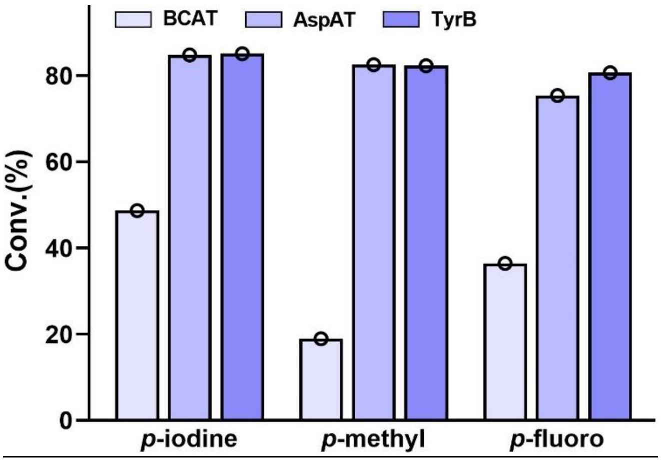

Supplementary figure 15 Synthesis of amino acids from keto acids by purified transaminase. Purified transaminase derived from *E. coli* is used to catalyze the reaction from keto acids to amino acids. 1 mL mixture contained 1 mM phenylpyruvic acid (1% DMSO), 5 mM L-glutamic acid (L-Glu), 0.1 mg·mL^{-1} ATs in phosphate buffer (50 mM Na_2HPO_4, pH 7.5, 50 mM NaCl). The mixture was stirred at room temperature (25 °C) and detected after 10 min. Source data are provided in Source Data file.

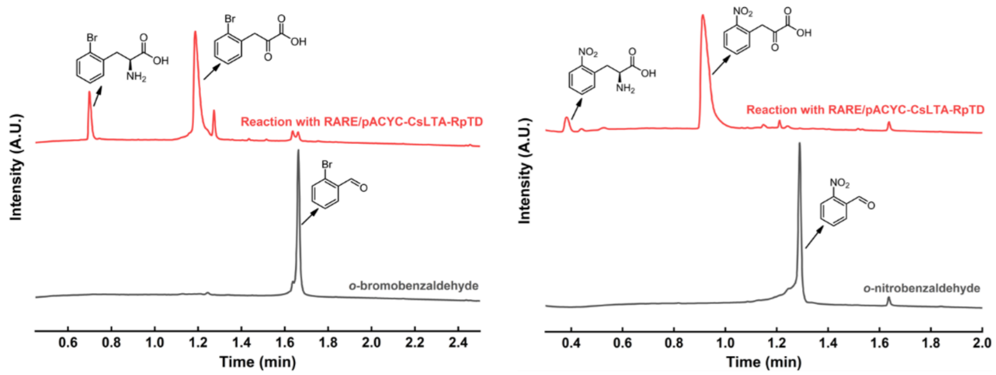

Supplementary Figure 16 Transaminase was the rate-limiting step for substrates with *ortho*-substituents. Reaction was carried out in 5 ml test tubes. 1 mM aromatic aldehydes, 10 mM Gly, 10 mM L-Glu and 10 mg lyophilized cell *E. coli* RARE/pACYC-CsLTA-RpTD in phosphate buffer (50 mM Na_2HPO_4, pH 7.4, 50 mM NaCl) with 10% DMSO. Reaction was at room temperature (25 °C) for 12 h. Transaminase was responsible for the low conversions resulting in the accumulation of α-keto acids.

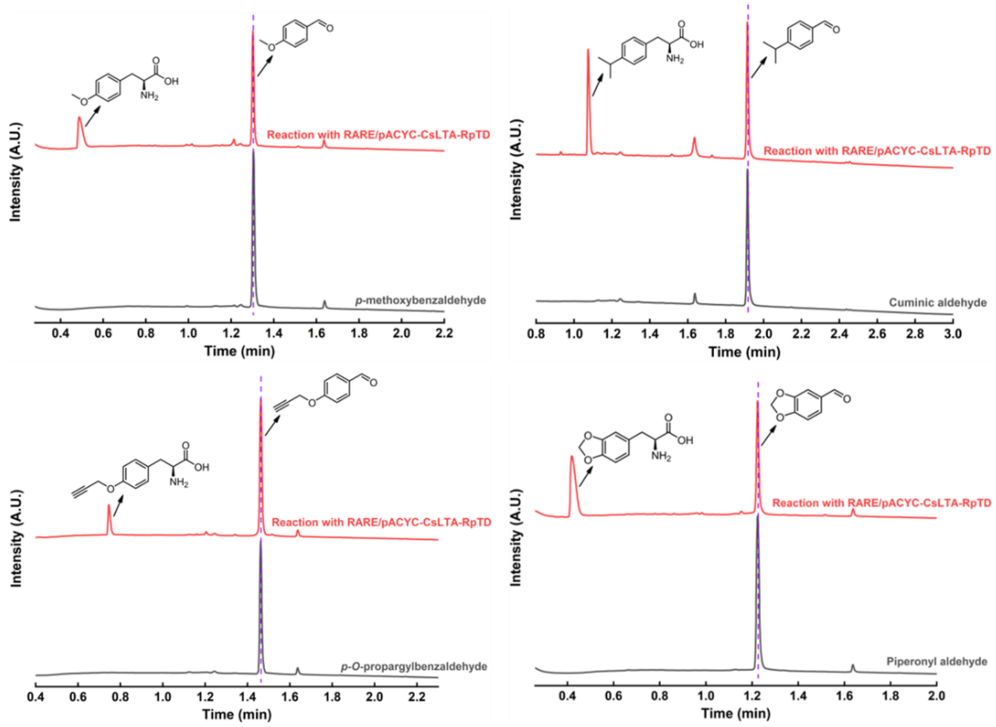

Supplementary Figure 17 Threonine aldolase was the rate-limiting step for substrates with *para*-substituents. Reaction was carried out in 5 ml test tubes. 1 mM aromatic aldehydes, 10 mM Gly, 10 mM L-Glu and 10 mg lyophilized cell *E. coli* RARE/pACYC-CsLTA-RpTD in phosphate buffer (50 mM Na_2HPO_4, pH 7.4, 50 mM NaCl) with 10% DMSO. Reaction was at room temperature (25 °C) for 12 h. Threonine aldolase was responsible for low conversions leading to the accumulation of aromatic aldehydes.

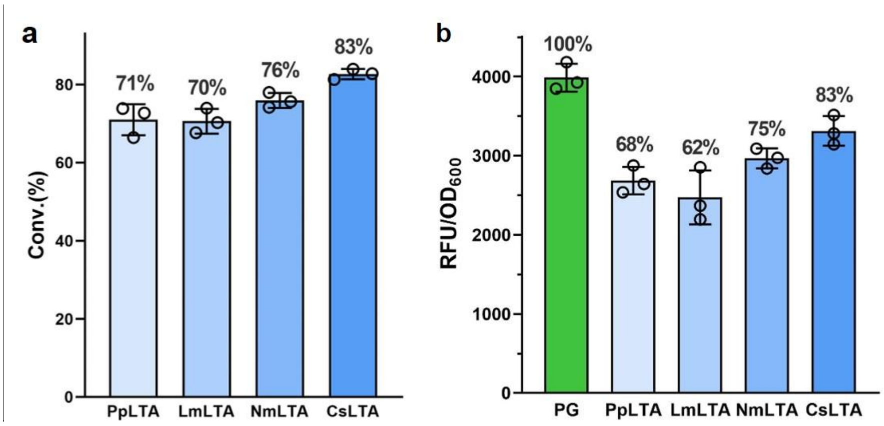

Supplementary Figure 18 sfGFP production level was related to ncAA titer. a. Transformation efficiency of *p*-iodobenzaldehyde by strains expressing different LTAs after 12 h. **b.** Detection of sfGFP(Y151pIF) fluorescence intensity of strains expressed different LTAs after 24 h fermentation. sfGFP bearing amber mutation was expressed in *E. coli* RARE (DE3) containing pIFRS/$tRNA^{Pyl}_{CUA}$ pair. 1 mM *p*-iodobenzaldehyde, 50 mM Gly and 20 μM PLP were added in the culture. The density of cells and the fluorescence intensity (Ex: 485 nm; Em: 528 nm) of sfGFP with incorporation of biosynthetic pIF was detected by a plate reader. Error bars represent the mean ± s.d. of n = 3 independent samples. Source data of **a** and **b** are provided in Source Data file.

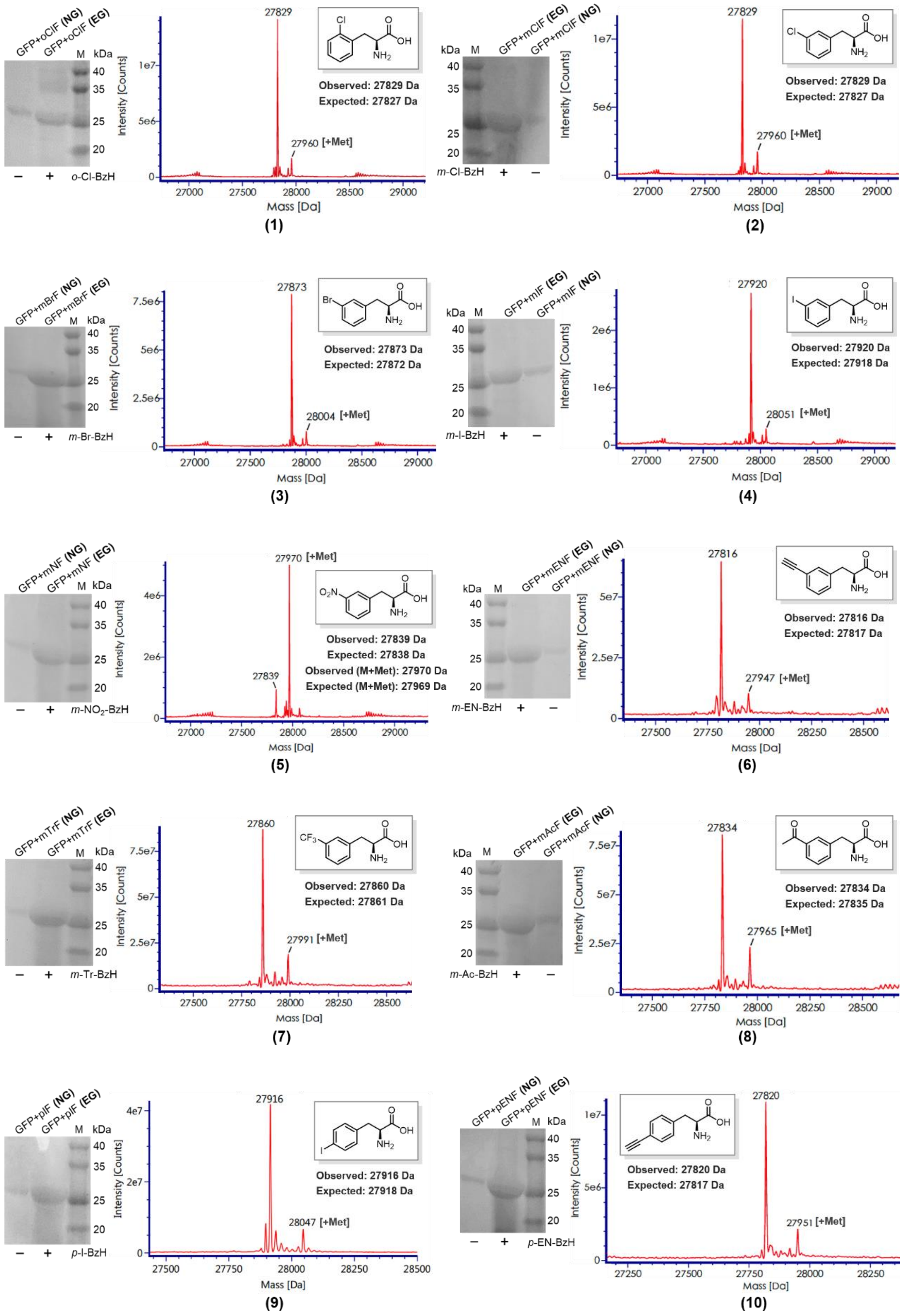

Supplementary Figure 19 High-resolution mass spectrometry of sfGFP with ncAAs modification. SfGFP was purified as described in the methods. Therein, 27970 **(5)** was mainly protein with non-cleavage of the N-terminal methionine residue. 27818 **(12)** was mainly protein with dehydrated boric acid groups. Images of uncropped gels were provided in Source Data file.

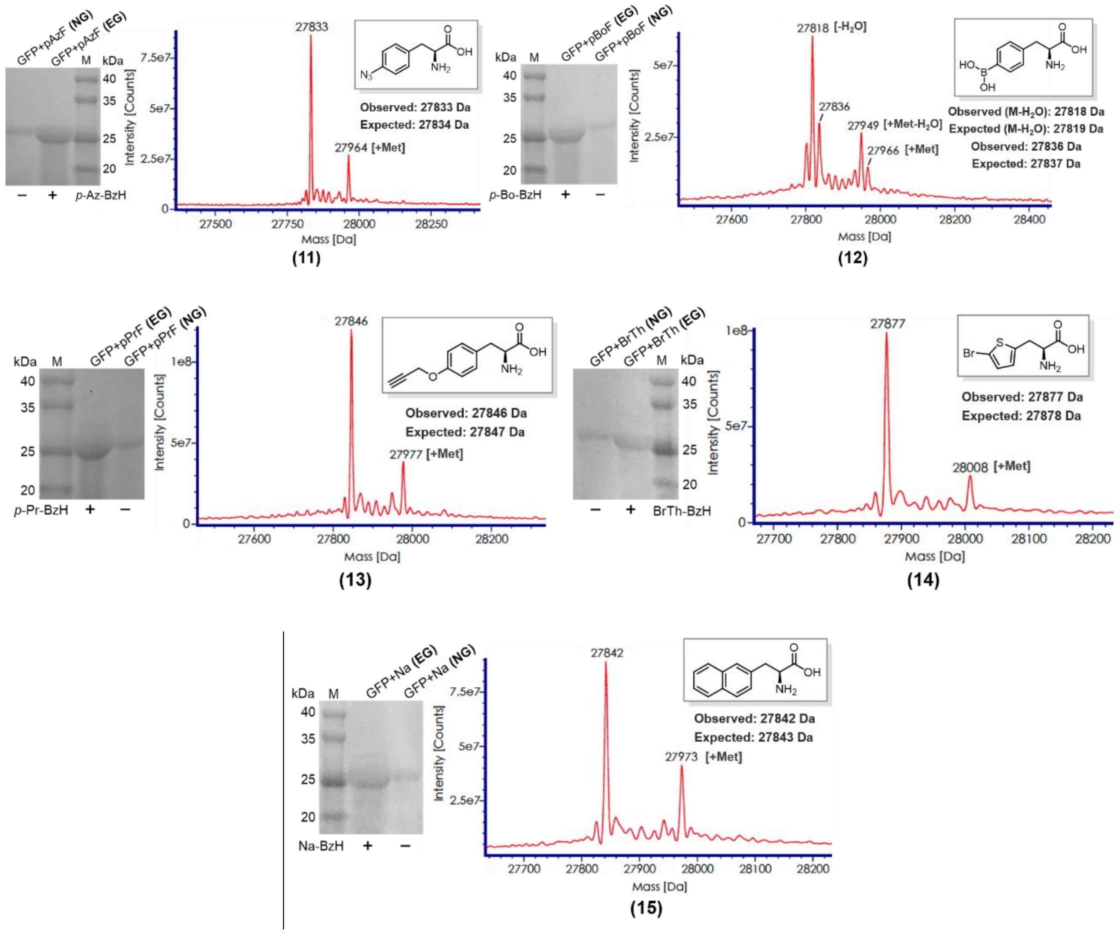

Supplementary Figure 19 (continued) High-resolution mass spectrometry of sfGFP with ncAAs modification. SfGFP was purified as described in the methods. Therein, 27970 **(5)** was mainly protein with non-cleavage of the N-terminal methionine residue. 27818 **(12)** was mainly protein with dehydrated boric acid groups. Images of uncropped gels were provided in Source Data file.

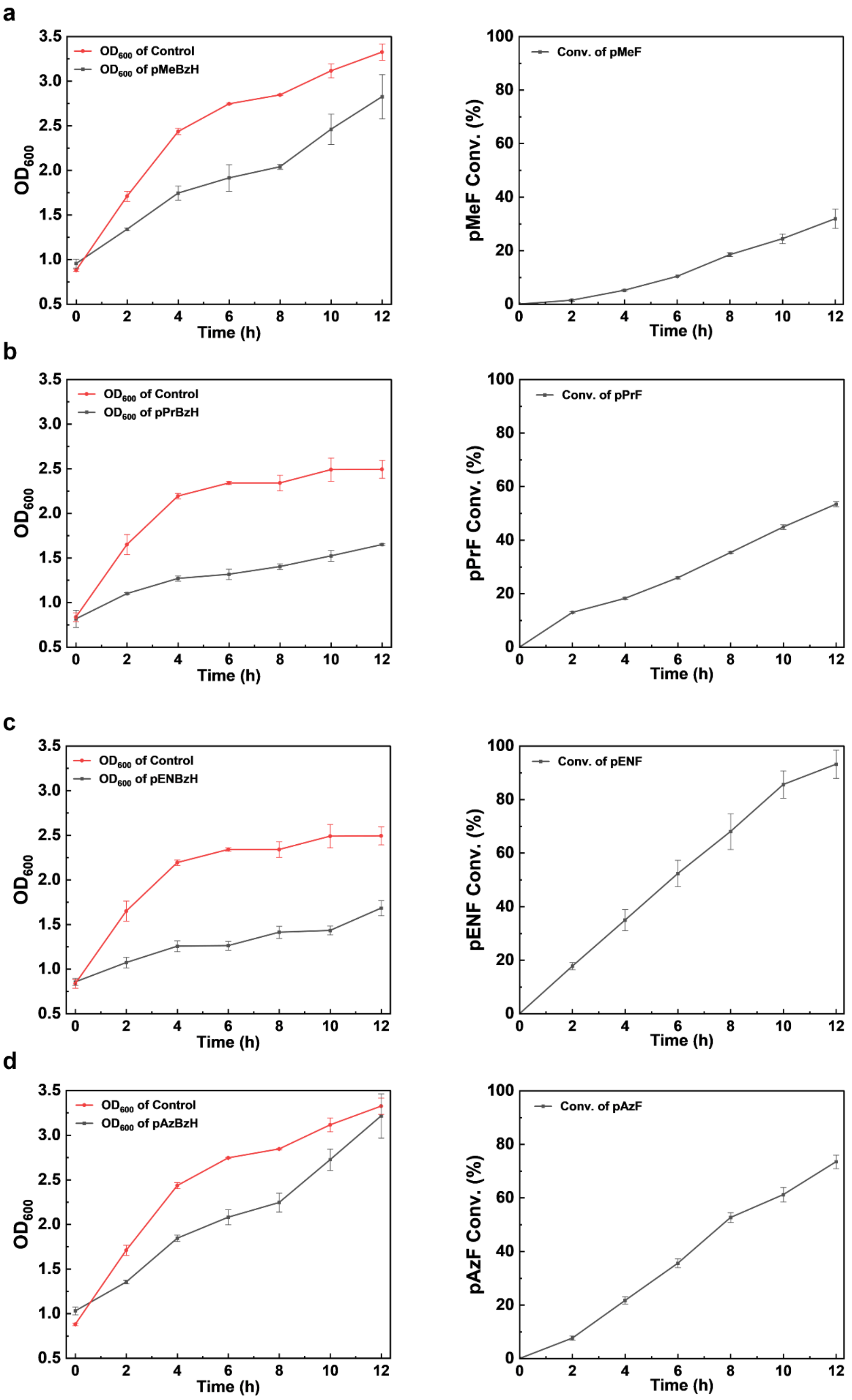

Supplementary Figure 20 The impact of aryl-aldehydes on cell growth of *E. coli* and ncAAs yields. *E. coli* RARE (DE3) harboring PpLTA and RpTD. 1 mM *p*-methylbenzaldehyde (pMeBzH) **(a)**, 1 mM *p*-propargyloxybenzaldehyde (pPrBzH) **(b)**, *p*-ethynylbenzaldehyde (pENBzH) **(c)** or 1 mM *p*-azidebenzaldehyde (pAzBzH) **(d)**, 50 mM Gly and 20 μM PLP were added to the culture medium at the same time of IPTG inducing expression when OD_{600} reached 1.0, which was regarded as 0 h. No reactive components were added to the control group. The strains were then induced at 30 °C for 12 h, and samples were taken every two hours to detect bacterial concentration (OD_{600}) and ncAAs conversion. Error bars represent the mean ± s.d. of

n = 3 independent samples. Source data of all figures are provided in Source Data file.

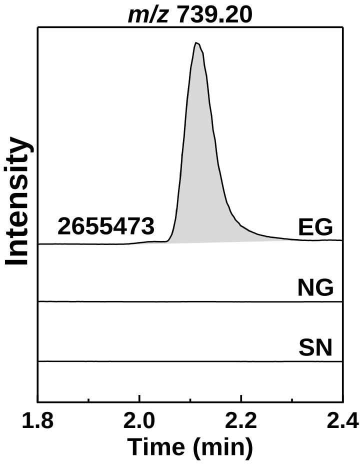

Supplementary Figure 21 Intracellular biosynthesis of macrocycle cyclo-CLLFVY. Wild type of Npu was transformed into *E. coli* RARE (DE3) and biosynthesized cyclo-CLLFVY (EG), whereas no product was detected in the culture of *E. coli* RARE (DE3) without the Npu encoding plasmid (NG) and supernatant of culture medium (SN). The peak corresponding to cyclo-CLLFVY is detected by MS-[M+H] single ion recording (SIR) channels in positive mode. The peak corresponding to cyclo-CLLFVY is highlighted. This sequence was used as a template for all subsequent ncAA incorporation studies. Source data are provided in Source Data file.

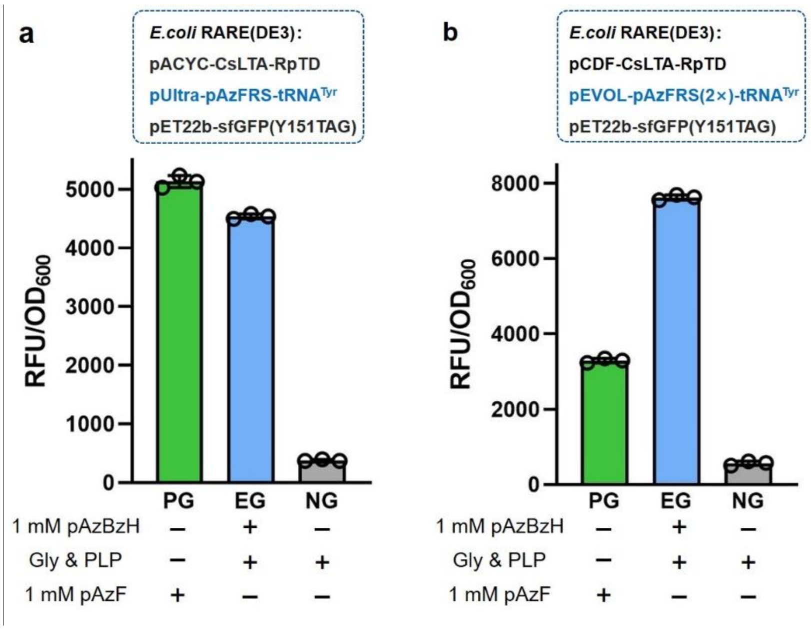

Supplementary Figure 22 Fluorescence intensity of sfGFP containing pAzF with one or two copies of pAzFRS. The fluorescence intensity of sfGFPY151TAG was measured in *E. coli* RARE (DE3) (CsLTA-RpTD) strains containing pAzFRS with one (**a**) or two (**b**) copies of *Mj*pAzFRS, after 24 hours of fermentation. The culture was supplemented with either 1 mM ncAAs (PG) or 1 mM aromatic aldehyde substrates (EG). Error bars represent the mean ± s.d. of n = 3 independent samples. Source data of **a** and **b** are provided in Source Data file.

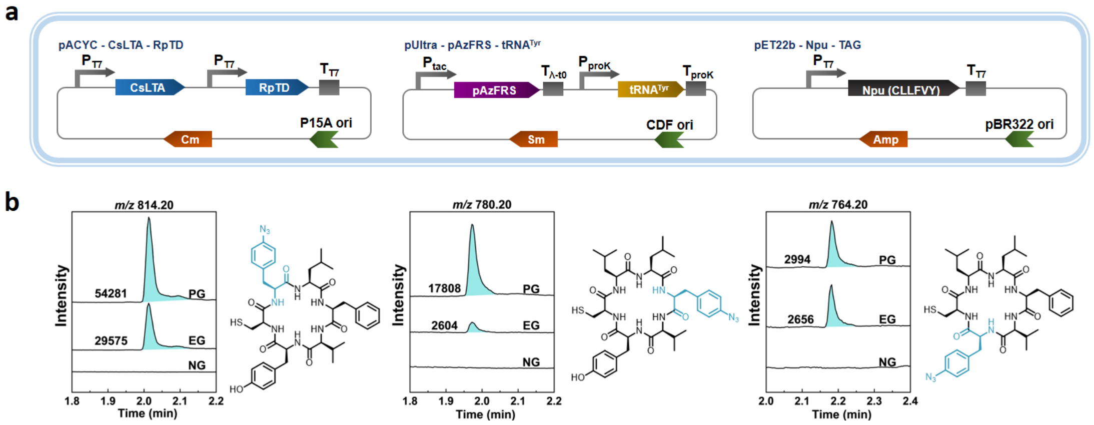

Supplementary Figure 23 Intracellular synthesis of peptide macrocycles with pUltra vector encoding pAzFRS. a. Plasmid design for coupling pAzF biosynthesis and incorporation into *Npu* of cyclo-CLLFVY. pACYCDuet-1 contained CsLTA and RpTD genes for cascade catalysis, pUltra vector contained mutant of *Mj*TyrRS/tRNA pair. tRNA expression was driven by a constitutive promoter, whereas other genes were expressed under IPTG induction. **b.** Cyclo-CLLFVY, with pAzF at positions 2, 4, or 6, was incorporated by the pAzFRS/$tRNA^{Tyr}$ pair. Source data of **b** are provided in Source Data file.

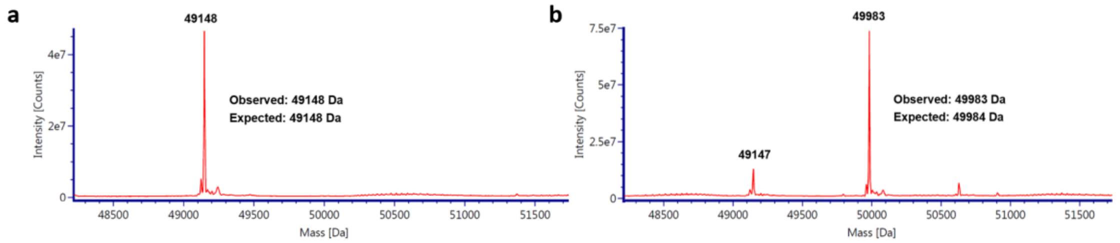

Supplementary Figure 24 High-resolution mass spectrometry determination of conjugation reaction of anti-HER2-Fab-A121pAzF and AF488-DBCO. a. High-resolution mass spectrometry of anti-HER2-Fab-A121pAzF. **b.** anti-HER2-Fab-A121pAzF was mixed with DBCO-AF488 (5.0 equiv.) and incubated at 25 °C for 7 h, the mixture was analyzed by high-resolution mass spectrometry. The peak of 49983 Da corresponds to protein conjugation.

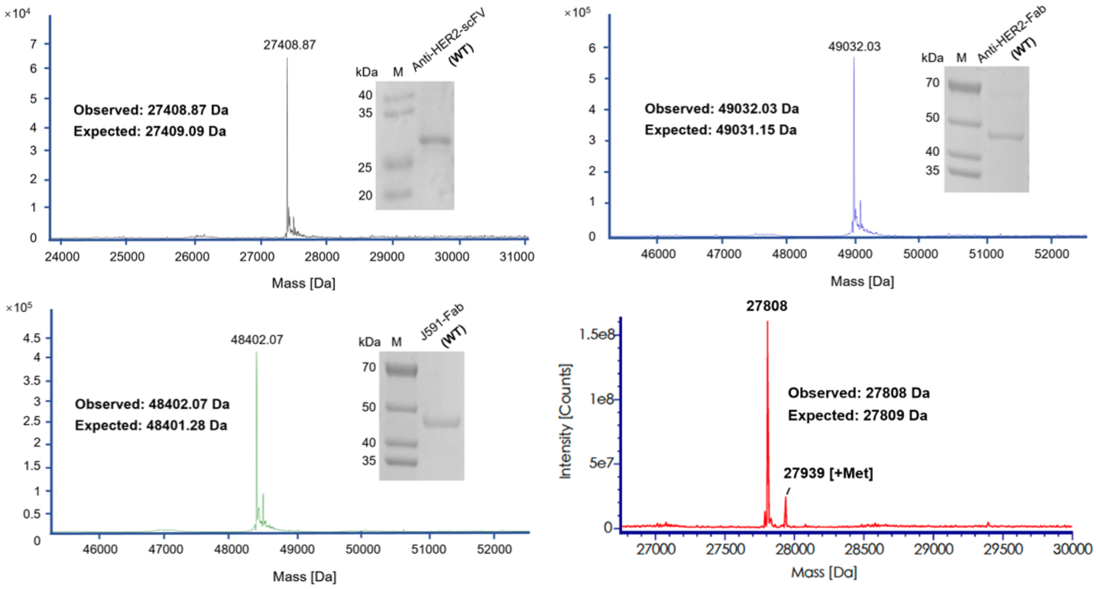

Supplementary Figure 25 High-resolution mass spectrometry of wild type sfGFP and antibody fragments. Images of uncropped gels were provided in Source Data file.

Supplementary Table 1 Expression yield of sfGFP with ncAA modification.

**Protein**	**Structure**	**Isolated yield**
sfGFP-oClF	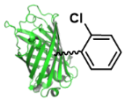	26.4 mg·L^{-1}
sfGFP-mClF	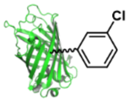	48.0 mg·L^{-1}
sfGFP-mBrF	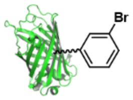	67.2 mg·L^{-1}
sfGFP-mIF	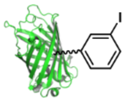	12.8 mg·L^{-1}
sfGFP-mNF	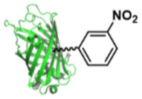	57.6 mg·L^{-1}
sfGFP-mENF	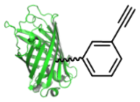	28.2 mg·L^{-1}
sfGFP-mTrF	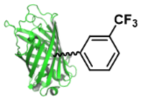	59.2 mg·L^{-1}
sfGFP-mAcF	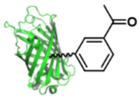	32.5 mg·L^{-1}
sfGFP-pIF	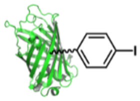	27.3 mg·L^{-1}
sfGFP-pENF	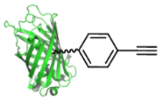	71.5 mg·L^{-1}
sfGFP-pAzF	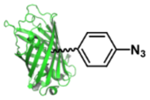	32.3 mg·L^{-1}
sfGFP-pBoF	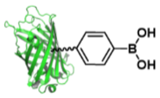	64.0 mg·L^{-1}
sfGFP-pPrF	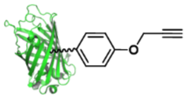	52.5 mg·L^{-1}

sfGFP-Na-Ala	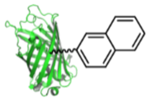	23.0 mg·L^{-1}
sfGFP-BrTh-Ala	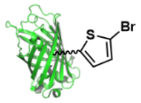	11.8 mg·L^{-1}
sfGFP-WT	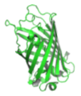	72.2 mg·L^{-1}

The sfGFP structure is derived from 6DQ0 in PDB.

Supplementary Table 2 Mass Spectrometry (MS) analysis of macrocycles.

Entry	Description	ncAAs	Chemical formula	Mass	Mass+H
1	CLLFVY	/	$C_{38}H_{54}N_6O_7S$	738.38	739.38
2	C[TAG]LFVY	pAzF	$C_{41}H_{51}N_9O_7S$	813.36	814.36
3	CLL[TAG]VY	pAzF	$C_{38}H_{53}N_9O_7S$	779.38	780.38
4	CLLFV[TAG]	pAzF	$C_{41}H_{51}N_9O_6S$	763.38	764.38
5	C[TAG]LFVY	pBoF	$C_{41}H_{53}BN_6O_9S$	816.37	817.37
6	CLL[TAG]VY	pBoF	$C_{38}H_{53}BN_6O_9S$	782.38	783.38
7	CLLFV[TAG]	pBoF	$C_{38}H_{55}BN_6O_8S$	766.39	767.39
8	C[TAG]LFVY	pENF	$C_{43}H_{52}N_6O_7S$	796.36	797.36
9	CLL[TAG]VY	pENF	$C_{40}H_{54}N_6O_7S$	762.38	763.38
10	CLLFV[TAG]	pENF	$C_{40}H_{54}N_6O_6S$	746.38	747.38
11	C[TAG]LFVY	pPrF	$C_{44}H_{54}N_6O_8S$	826.37	827.37
12	CLL[TAG]VY	pPrF	$C_{41}H_{56}N_6O_8S$	792.39	793.39
13	CLLFV[TAG]	pPrF	$C_{41}H_{56}N_6O_7S$	776.39	777.39

Supplementary Table 3

Entry	Description	Isolated yield
1	Her2-scFv-WT	3.20 $mg \cdot L^{-1}$
2	Her2-scFv-S9pAzF	1.50 $mg \cdot L^{-1}$
3	Her2-scFv-K42pAzF	1.44 $mg \cdot L^{-1}$
4	Her2-Fab-WT	2.82 $mg \cdot L^{-1}$
5	Her2-Fab-A121pAzF	1.53 $mg \cdot L^{-1}$
6	J591-Fab-WT	5.25 $mg \cdot L^{-1}$
7	J591-Fab-A121pAzF	2.93 $mg \cdot L^{-1}$

Supplementary Methods

Supplementary method 1. General information

Strains and Chemicals. Genes were synthesized, codon-optimized and cloned into pET28a or pET22b expression plasmids by Genewiz (Provided in Supplementary data 1). Primers were listed in Supplementary data 2. The competent cells *E. coli* BL21 (DE3) was purchased from Sangon Biotech, and *E. coli* MG1655 (DE3) was purchased from Zomanbio. The expression strain *E. coli* RARE (DE3) was purchased from Addgene and made into competent cells. The preparation of competent cells was relied on the super receptive cell preparation kit of Sangon Biotech. The antibiotics used in this study and the media components tryptone, yeast extract, NaCl, isopropyl *β*-D-thiogalactoside (IPTG) were also purchased from Sangon Biotech. Homologous recombinase and T4 ligase were purchased from Vazyme Biotech, and all kinds of restriction endonuclease were purchased from Takara. All commercial substrates were purchased from Bidepharm and used without further purification. Deuterated solvents were purchased from Energy Chemical. A high-pressure homogenizer and Ni-NTA Union 6FF agarose were purchased from Union Biotech.

Detection and Characterization. Whole-cell catalytic, *E. coli* fermentation transformation reaction activity and macrocyclic peptides detection were measured on a Waters UPLC-MS with a 2998 PDA detector, a Waters 3100 SQDMS detector, a sample manager and a binary solvent manager using an ACQUITY UPLC® BEH C18 column (2.1×50 mm, 1.7 μm) under a 5-95% B gradient lasting 2.5 min (solvent system composed of A H_2O (0.1% FA) and B CH_3CN (0.1% FA); flow rate, 0.8 $mL{\cdot}min^{-1}$; Column temperature, 40 °C, and UV absorbance, 210 nm). All the samples were analyzed with n =1 replicates. UV, TICs and XICs spectra were analyzed, collected and exported using Waters Empower. SepaFlash® column chromatography was performed using a Biotage Flash Purification system. NMR spectra were recorded using Bruker AVANCE III 400, 500 and 600 MHz instruments. Standard abbreviations indicating multiplicity were used as follows: singlet (s), doublet (d), doublet of doublets (dd), doublet of doublet of doublets (ddd), doublet of triplets (dt), triplet (t), triplet of doublets (td), quartet (q), and multiplet (m). Chemical shifts were reported in ppm (*δ*), coupling constants (*J*) in hertz. High-resolution mass spectra (HRMS) were recorded on Agilent G6520 Q-TOF using electrospray

positive ionization, respectively. Positive total ion scans were observed from 100 to 1000 *m/z*. Optical rotations were measured with a Rudolph Research Analytical Autopol automatic polarimeter (Hackettstown, NJ). Cell fluorescence was detected by plate reader Molecular Devices-MD M5e Proteins were analyzed by sodium dodecyl sulfate-polyacrylamide gel electrophoresis (SDS-PAGE) under denaturing conditions and high-resolution mass spectrometry with Agilent 6545 Q-TOF.construction

Construction of threonine aldolase and threonine deaminase co-expression plasmids. The plasmid pET28a-RpTD, which contains *R. pickettii* threonine deaminase (RpTD), was synthesized by Genewiz. The vector pACYCDuet-1, which carries the P15A replicative origin and a chloramphenicol (Cm) resistance marker, were used as templates. Both plasmids were double digested with NdeI and XhoI. The RpTD gene was then inserted into the multiple cloning site 2 (MCS-2) of pACYCDuet-1 using T4 ligase, resulting in the recombinant plasmid pACYC-RpTD. Four threonine aldolase genes (PpLTA from *Pseudomonas putida*, LmLTA from *Leishmania major* strain Friedlin, NmLTA from *Neptunomonas marina*, and CsLTA from *Cellulosilyticum sp.*) were codon-optimized, synthesized, and cloned into pET28a by Genewiz. These LTA genes were then amplified from the corresponding plasmids using primers LTA-F and LTA-R. To generate co-expression plasmids pACYC-(Pp/Lm/Nm/Cs)LTA-RpTD, plasmid pACYC-RpTD was linearized and the 6×His tag was removed using primers pA-Rp-line-F and pA-Rp-line-R. Each LTA gene was individually cloned into MCS-1 of the linearized pACYC-RpTD plasmid via Gibson assembly. The resulting plasmids were sequenced to confirm the correct gene insertions.

Construction of aminoacyl-tRNA synthetase/tRNA (aaRS/tRNA) expression plasmids for genetic code expansion. Various mutants of *Methanosarcina mazei* pyrrolysyl-tRNA synthetase (*Mm*PylRS), *Methanosarcina barkeri* pyrrolysyl-tRNA synthetase (*Mb*PylRS), and *Methanococcus jannaschii* tyrosyl-tRNA synthetase (*Mj*TyrRS), along with their orthogonal tRNAs, were selected for the incorporation of different noncanonical amino acids (ncAAs) synthesized *in situ*. The plasmid pCDF-*Mm*PylRS(2A)-$tRNA^{Pyl}_{CUA}$, which contains the CDF replicative origin and a kanamycin (Kan) resistance marker, was used as the template. This plasmid includes a double-mutated *Mm*PylRS gene (N346A/C348A). The *Mm*PylRS(2A) gene

was replaced by various mutants of PylRS (including pIFRS, with mutation sites L305M/Y306L/L309S/N346S/C348M in *Mm*PylRS, and PylHRS, with mutation sites L270I/Y271F/L274G/C313F/Y349F in *Mb*PylRS) via Gibson assembly. The ligated products were sequenced to confirm the correct identity of the inserts.

Additionally, the plasmid pUltra-pCNFRS-tRNA$^{Tyr}_{CUA}$, containing the CDF replicative origin and a streptomycin (Sm) resistance marker, was used as another template. This plasmid carries a mutated *Mj*TyrRS gene (Y32L/L65V/F108W/Q109M/D158G). The pCNFRS gene was replaced with various mutants of *Mj*TyrRS (including pAzFRS with mutation sites Y32T/E107N/D158P/I159L/L162Q/D286R, pBoFRS with mutation sites Y32S/L65A/H70M/D158S/L162E, and NaRS with mutation sites Y32L/D158P/I159A/L162Q/A167V) through Gibson assembly. The resulting plasmids were sequenced to confirm the identity of the mutant genes.

Construction of target protein containing ncAAs expression plasmids. The sfGFP, Anti-Her2-scFV, Anti-Her2-Fab, J591-Fab and Npu of cyclo-CLLFVY genes were codon-optimized, synthesized and cloned into pET-22b containing pBR322 replicative origin and ampicillin (Amp) resistance marker with 6×his tag in C-terminal. StII signal peptide was added in front of scFV or each chain of Fab for periplasmic translocation. Primers in Supplementary data 2 were used for reverse PCR to linearize plasmids with homologous arms on both ends. Linearized plasmids were recycled by homologous recombination and introduced Y151TAG mutation in sfGFP, S9TAG or K42TAG in Anti-Her2-scFv, A121TAG in heavy chain of Anti-Her2-Fab, A116TAG in J591-Fab and 2/4/6TAG for cyclo-CLLFVY in *Npu*. The plasmid products were sequenced to confirm identity. All constructed plasmids were stored at -20 °C.

Supplementary method 2. Synthesis and characterization of ncAA products

Whole-cell catalyst preparation. The obtained co-expression plasmids for LTAs and RpTD were chemically transformed into *E. coli* BL21 (DE3) competent cells. Single colonies, cultured overnight on agar plates, were selected and inoculated into LB medium containing 35 μg·mL^{-1} chloramphenicol (Cm), and grown overnight at 37 °C with shaking at 220 rpm. The overnight culture was then transferred to fresh LB medium containing Cm at a 1% inoculum

rate and grown at 37 °C with shaking at 220 rpm until the optical density at 600 nm (OD_{600}) reached 0.8-1.0. At this point, IPTG was added to a final concentration of 0.5 mM, and protein expression was performed by lowering the incubation temperature to 18 °C and shaking at 180 rpm for 20 hours. Following induction, the bacterial cells were harvested by centrifugation at 4 °C, 10,000 rpm for 10 minutes. The collected pellet was washed 2-3 times with PBS, then re-suspended in PBS and freeze-dried.

Optimization of whole-cell reaction conditions. The reaction mixture contained 1 mM aromatic aldehyde substrates, 5 mM Gly, 5 mM L-Glu, 5 mg mL^{-1} of whole-cell catalyst, and 10% DMSO. The reaction buffer (pH 7.5) consisted of 50 mM Na_2HPO_4 and 50 mM NaCl. The total volume was 1 mL, and the mixture was stirred at room temperature (25 °C). Samples were taken at specified time intervals. To stop the reaction, the samples were diluted 5-fold with 50% methanol (MeOH) and filtered through a membrane for UPLC-MS analysis. Next, various reaction conditions were optimized.

For optimization of the catalyst loading and substrate concentration, different substrate concentrations were tested in the 1 mL reaction system, using *E. coli* BL21 (DE3)/pACYC-PpLTA-RpTD lyophilized cells at concentrations of 5 mg mL^{-1} and 10 mg mL^{-1} as catalysts for each substrate concentration gradient. A total of twelve reaction groups were set up, with three parallel experiments for each condition.

For the optimization of Gly concentration, the reaction was carried out at different Gly concentrations, with three parallel experiments in each group. The L-Glu concentration optimization was conducted in the same manner as for Gly. In all cases, the reaction conditions remained consistent except for the variable being tested.

Additionally, PpLTA in the cascade pathway was replaced with three other LTAs, and samples were taken at 6 h and 12 h to compare the catalytic efficiency across the different LTAs.

Purification and characterization of rare aromatic ncAAs.

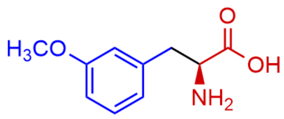

F1 (38.3%, 93.4 mg)

F1: (*S*)-3-methoxyphenylalanine

Synthesis of (*S*)-3-methoxyphenylalanine (**F1**) by lyophilized *E. coli* (PpLTA-RpTD) whole-cell catalyst. Prepare 1 L phosphate buffer ($Na_2HPO_4 \cdot 12H_2O$: 18 g, 50 mM; NaCl: 2.9 g, 50 mM; 1000 mL H_2O; pH 7.4), 6 mL 3-methoxybenzaldehyde stock solution (0.2042 g, 250 mM, dissolved by DMSO), 22 mL Gly stock solution (1.0313 g, 625 mM, dissolved by phosphate buffer), 42 mL L-Glu stock solution (2.8941 g, 468.8 mM, dissolved by phosphate buffer with 0.8 mL of 5M NaOH solution) in advance. Weigh 0.5 g lyophilized cell *E. coli* BL21 (DE3)/pACYC-PpLTA-RpTD into a round-bottom flask, then add 15 mL phosphate buffer, 10 mL Gly stock solution, 20 mL L-Glu stock solution and 5 mL 3-methoxybenzaldehyde stock solution sequentially. Stir the mixture at room temperature (25 °C) for 12 h and quench the reaction with 2 mL of 5 M HCl solution. Then centrifuge the reaction at 13000 rpm for 10 min and collect the supernatant carefully. Separate the product from the supernatant by medium-pressure liquid chromatography (preparative HPLC performed on a Biotage Isolera One using a SepaFlash® column with an UV detector) at last. The final mass of the product is 93.4 mg and the total yield is 38.3%. The product has been characterized by HR-MS and NMR spectroscopy.

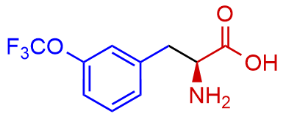

F2 (33.9%, 105.6 mg)

F2: (*S*)-3-(trifluoromethoxy)phenylalanine

Synthesis of (*S*)-3-(trifluoromethoxy)phenylalanine (**F2**) by lyophilized *E. coli* (PpLTA-RpTD) whole-cell catalyst. Prepare 1 L phosphate buffer ($Na_2HPO_4 \cdot 12H_2O$: 18 g, 50 mM; NaCl: 2.9 g, 50 mM; 1000 mL H_2O; pH 7.4), 6 mL 3-(trifluoromethoxy)benzaldehyde stock solution (0.2852 g, 250 mM, dissolved by DMSO), 22 mL Gly stock solution (1.0313 g, 625 mM, dissolved by phosphate buffer), 42 mL L-Glu stock solution (2.8941 g, 468.8 mM, dissolved by phosphate buffer with 0.8 mL of 5M NaOH solution) in advance. Weigh 0.5 g lyophilized cell

E. coli BL21 (DE3)/pACYC-PpLTA-RpTD into a round-bottom flask, then add 15 mL phosphate buffer, 10 mL Gly stock solution, 20 mL L-Glu stock solution and 5 mL 3-(trifluoromethoxy)benzaldehyde stock solution sequentially. Stir the mixture at room temperature (25 °C) for 12 h and quench the reaction with 2 mL of 5 M HCl solution. Then centrifuge the reaction at 13000 rpm for 10 min and collect the supernatant carefully. Separate the product from the supernatant by medium-pressure liquid chromatography (preparative HPLC performed on a Biotage Isolera One using a SepaFlash® column with an UV detector) at last. The final mass of the product is 105.6 mg and the total yield is 33.9%. The product has been characterized by HR-MS and NMR spectroscopy.

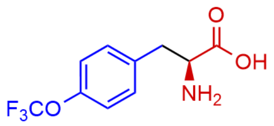

F3 (39.3%, 122.3 mg)

F3: (*S*)-4-(trifluoromethoxy)phenylalanine

Synthesis of (*S*)-4-(trifluoromethoxy)phenylalanine (**F3**) by lyophilized *E. coli* (PpLTA-RpTD) whole-cell catalyst. Prepare 1 L phosphate buffer ($Na_2HPO_4 \cdot 12H_2O$: 18 g, 50 mM; NaCl: 2.9 g, 50 mM; 1000 mL H_2O; pH 7.4), 6 mL 4-(trifluoromethoxy)benzaldehyde stock solution (0.2852 g, 250 mM, dissolved by DMSO), 22 mL Gly stock solution (1.0313 g, 625 mM, dissolved by phosphate buffer), 42 mL L-Glu stock solution (2.8941 g, 468.8 mM, dissolved by phosphate buffer with 0.8 mL of 5M NaOH solution) in advance. Weigh 0.5 g lyophilized cell *E. coli* BL21 (DE3)/pACYC-PpLTA-RpTD into a round-bottom flask, then add 15 mL phosphate buffer, 10 mL Gly stock solution, 20 mL L-Glu stock solution and 5 mL 4-(trifluoromethoxy)benzaldehyde stock solution sequentially. Stir the mixture at room temperature (25 °C) for 12 h and quench the reaction with 2 mL of 5 M HCl solution. Then centrifuge the reaction at 13000 rpm for 10 min and collect the supernatant carefully. Separate the product from the supernatant by medium-pressure liquid chromatography (preparative HPLC performed on a Biotage Isolera One using a SepaFlash® column with an UV detector) at last. The final mass of the product is 122.3 mg and the total yield is 39.3%. The product has been characterized by HR-MS and NMR spectroscopy.

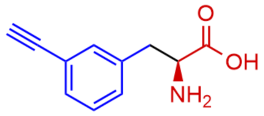

F4 (65.6%, 155.0 mg)

F4: (*S*)-3-ethynylphenylalanine

Synthesis of (*S*)-3-ethynylphenylalanine (**F4**) by lyophilized *E. coli* (PpLTA-RpTD) whole-cell catalyst. Prepare 1 L phosphate buffer ($Na_2HPO_4 \cdot 12H_2O$: 18 g, 50 mM; NaCl: 2.9 g, 50 mM; 1000 mL H_2O; pH 7.4), 6 mL 3-ethynylbenzaldehyde stock solution (0.1952 g, 250 mM, dissolved by DMSO), 22 mL Gly stock solution (1.0313 g, 625 mM, dissolved by phosphate buffer), 42 mL L-Glu stock solution (2.8941 g, 468.8 mM, dissolved by phosphate buffer with 0.8 mL of 5M NaOH solution) in advance. Weigh 0.5 g lyophilized cell *E. coli* BL21 (DE3)/pACYC-PpLTA-RpTD into a round-bottom flask, then add 15 mL phosphate buffer, 10 mL Gly stock solution, 20 mL L-Glu stock solution and 5 mL 3-ethynylbenzaldehyde stock solution sequentially. Stir the mixture at room temperature (25 °C) for 12 h and quench the reaction with 2 mL of 5 M HCl solution. Then centrifuge the reaction at 13000 rpm for 10 min and collect the supernatant carefully. Separate the product from the supernatant by medium-pressure liquid chromatography (preparative HPLC performed on a Biotage Isolera One using a SepaFlash® column with an UV detector) at last. The final mass of the product is 155.0 mg and the total yield is 65.6%. The product has been characterized by HR-MS and NMR spectroscopy.

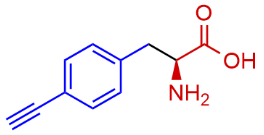

F5 (38.1%, 90.0mg)

F5: (*S*)-4-ethynylphenylalanine

Synthesis of (*S*)-4-ethynylphenylalanine (**F5**) by lyophilized *E. coli* (PpLTA-RpTD) whole-cell catalyst. Prepare 1 L phosphate buffer ($Na_2HPO_4 \cdot 12H_2O$: 18 g, 50 mM; NaCl: 2.9 g, 50 mM; 1000 mL H_2O; pH 7.4), 6 mL 4-ethynylbenzaldehyde stock solution (0.1952 g, 250 mM, dissolved by DMSO), 22 mL Gly stock solution (1.0313 g, 625 mM, dissolved by phosphate buffer), 42 mL L-Glu stock solution (2.8941 g, 468.8 mM, dissolved by phosphate buffer with 0.8 mL of 5M NaOH solution) in advance. Weigh 0.5 g lyophilized cell *E. coli* BL21

(DE3)/pACYC-PpLTA-RpTD into a round-bottom flask, then add 15 mL phosphate buffer, 10 mL Gly stock solution, 20 mL L-Glu stock solution and 5 mL 4-ethynylbenzaldehyde stock solution sequentially. Stir the mixture at room temperature (25 °C) for 12 h and quench the reaction with 2 mL of 5 M HCl solution. Then centrifuge the reaction at 13000 rpm for 10 min and collect the supernatant carefully. Separate the product from the supernatant by medium-pressure liquid chromatography (preparative HPLC performed on a Biotage Isolera One using a SepaFlash® column with an UV detector) at last. The final mass of the product is 90.0 mg and the total yield is 38.1%. The product has been characterized by HR-MS and NMR spectroscopy.

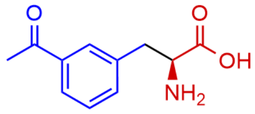

F6 (70.4%, 182.1mg)

F6: (*S*)-3-acetylphenylalanine

Synthesis of (*S*)-3-acetylphenylalanine (**F6**) by lyophilized *E. coli* (PpLTA-RpTD) whole-cell catalyst. Prepare 1 L phosphate buffer ($Na_2HPO_4 \cdot 12H_2O$: 18 g, 50 mM; NaCl: 2.9 g, 50 mM; 1000 mL H_2O; pH 7.4), 6 mL 3-acetylbenzaldehyde stock solution (0.2222 g, 250 mM, dissolved by DMSO), 22 mL Gly stock solution (1.0313 g, 625 mM, dissolved by phosphate buffer), 42 mL L-Glu stock solution (2.8941 g, 468.8 mM, dissolved by phosphate buffer with 0.8 mL of 5M NaOH solution) in advance. Weigh 0.5 g lyophilized cell *E. coli* BL21 (DE3)/pACYC-PpLTA-RpTD into a round-bottom flask, then add 15 mL phosphate buffer, 10 mL Gly stock solution, 20 mL L-Glu stock solution and 5 mL 3-acetylbenzaldehyde stock solution sequentially. Stir the mixture at room temperature (25 °C) for 12 h and quench the reaction with 2 mL of 5 M HCl solution. Then centrifuge the reaction at 13000 rpm for 10 min and collect the supernatant carefully. Separate the product from the supernatant by medium-pressure liquid chromatography (preparative HPLC performed on a Biotage Isolera One using a SepaFlash® column with an UV detector) at last. The final mass of the product is 182.1 mg and the total yield is 70.4%. The product has been characterized by HR-MS and NMR spectroscopy.

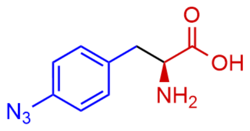

F7 (50.9%, 65.5 mg)

F7: (*S*)-4-azidophenylalanine

Synthesis of (*S*)-4-azidophenylalanine (**F7**) by lyophilized *E. coli* (PpLTA-RpTD) whole-cell catalyst. Prepare 1 L phosphate buffer ($Na_2HPO_4 \cdot 12H_2O$: 18 g, 50 mM; NaCl: 2.9 g, 50 mM; 1000 mL H_2O; pH 7.4), 2.6 mL 4-azidobenzaldehyde stock solution (0.0956 g, 250 mM, dissolved by DMSO), 22 mL Gly stock solution (1.0313 g, 625 mM, dissolved by phosphate buffer), 42 mL L-Glu stock solution (2.8941 g, 468.8 mM, dissolved by phosphate buffer with 0.8 mL of 5M NaOH solution) in advance. Weigh 0.5 g lyophilized cell *E. coli* BL21 (DE3)/pACYC-PpLTA-RpTD into a round-bottom flask, then add 7.5 mL phosphate buffer, 5 mL Gly stock solution, 10 mL L-Glu stock solution and 2.5 mL 4-azidobenzaldehyde stock solution sequentially. Stir the mixture at room temperature (25 °C) for 12 h and quench the reaction with 1 mL of 5 M HCl solution. Then centrifuge the reaction at 13000 rpm for 10 min and collect the supernatant carefully. Separate the product from the supernatant by medium-pressure liquid chromatography (preparative HPLC performed on a Biotage Isolera One using a SepaFlash® column with an UV detector) at last. The final mass of the product is 65.5 mg and the total yield is 50.9%. The product has been characterized by HR-MS and NMR spectroscopy.

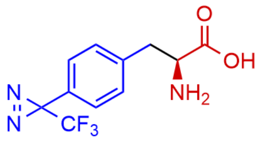

F8 (67.5%, 92.2 mg)
(5 mM substrate)

F8: (*S*)-4-(3-(trifluoromethyl)-3*H*-diazirin)phenylalanine

Synthesis of (*S*)-4-(3-(trifluoromethyl)-3*H*-diazirin)phenylalanine (**F8**) by lyophilized *E. coli* (PpLTA-RpTD) whole-cell catalyst. Prepare 1 L phosphate buffer ($Na_2HPO_4 \cdot 12H_2O$: 18 g, 50 mM; NaCl: 2.9 g, 50 mM; 1000 mL H_2O; pH 7.4), 2.1 mL 4-(3-(trifluoromethyl)-3H-diazirin)benzaldehyde stock solution (0.1124 g, 250 mM, dissolved by DMSO), 22 mL Gly stock solution (1.0313 g, 625 mM, dissolved by phosphate buffer), 42 mL L-Glu stock solution

(2.8941 g, 468.8 mM, dissolved by phosphate buffer with 0.8 mL of 5M NaOH solution) in advance. Weigh 1 g lyophilized cell *E. coli* BL21 (DE3)/pACYC-PpLTA-RpTD into a round-bottom flask, then add 86 mL phosphate buffer, 4 mL Gly stock solution, 8 mL L-Glu stock solution and 2 mL 4-(3-(trifluoromethyl)-3H-diazirin)benzaldehyde stock solution sequentially. Stir the mixture at room temperature (25 °C) for 12 h and quench the reaction with 4 mL of 5 M HCl solution. Then centrifuge the reaction at 13000 rpm for 10 min and collect the supernatant carefully. Separate the product from the supernatant by medium-pressure liquid chromatography (preparative HPLC performed on a Biotage Isolera One using a SepaFlash® column with an UV detector) at last. The final mass of the product is 92.2 mg and the total yield is 67.5%. The product has been characterized by HR-MS and NMR spectroscopy.

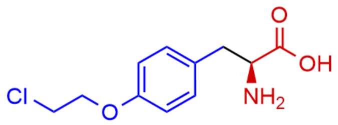

F9 (28.8%, 87.4 mg)

F9: (*S*)-4-(2-chloroethoxy)phenylalanine

Synthesis of (*S*)-4-(2-chloroethoxy)phenylalanine (**F9**) by lyophilized *E. coli* (PpLTA-RpTD) whole-cell catalyst. Prepare 1 L phosphate buffer ($Na_2HPO_4 \cdot 12H_2O$: 18 g, 50 mM; NaCl: 2.9 g, 50 mM; 1000 mL H_2O; pH 7.4), 5.5 mL 4-(2-chloroethoxy)benzaldehyde stock solution (0.2539 g, 250 mM, dissolved by DMSO), 22 mL Gly stock solution (1.0313 g, 625 mM, dissolved by phosphate buffer), 42 mL L-Glu stock solution (2.8941 g, 468.8 mM, dissolved by phosphate buffer with 0.8 mL of 5M NaOH solution) in advance. Weigh 0.5 g lyophilized cell *E. coli* BL21 (DE3)/pACYC-PpLTA-RpTD into a round-bottom flask, then add 15 mL phosphate buffer, 10 mL Gly stock solution, 20 mL L-Glu stock solution and 5 mL 4-(2-chloroethoxy)benzaldehyde stock solution sequentially. Stir the mixture at room temperature (25 °C) for 12 h and quench the reaction with 2 mL of 5 M HCl solution. Then centrifuge the reaction at 13000 rpm for 10 min and collect the supernatant carefully. Separate the product from the supernatant by medium-pressure liquid chromatography (preparative HPLC performed on a Biotage Isolera One using a SepaFlash® column with an UV detector) at last. The final mass of the product is 87.4 mg and the total yield is 28.8%. The product has been characterized by HR-MS and NMR spectroscopy.

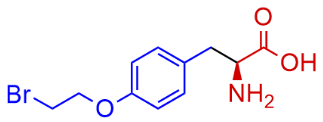

F10 (19.1%, 20.5 mg)

F10: (*S*)-4-(2-bromoethoxy)phenylalanine

Synthesis of (*S*)-4-(2-bromoethoxy)phenylalanine (**F10**) by lyophilized *E. coli* (PpLTA-RpTD) whole-cell catalyst. Prepare 1 L phosphate buffer ($Na_2HPO_4 \cdot 12H_2O$: 18 g, 50 mM; NaCl: 2.9 g, 50 mM; 1000 mL H_2O; pH 7.4), 1.6 mL 4-(2-bromoethoxy)benzaldehyde stock solution (0.0916 g, 250 mM, dissolved by DMSO), 22 mL Gly stock solution (1.0313 g, 625 mM, dissolved by phosphate buffer), 42 mL L-Glu stock solution (2.8941 g, 468.8 mM, dissolved by phosphate buffer with 0.8 mL of 5M NaOH solution) in advance. Weigh 0.15 g lyophilized cell *E. coli* BL21 (DE3)/pACYC-PpLTA-RpTD into a round-bottom flask, then add 4.5 mL phosphate buffer, 3 mL Gly stock solution, 6 mL L-Glu stock solution and 1.5 mL 4-(2-bromoethoxy)benzaldehyde stock solution sequentially. Stir the mixture at room temperature (25 °C) for 12 h and quench the reaction with 1 mL of 5 M HCl solution. Then centrifuge the reaction at 13000 rpm for 10 min and collect the supernatant carefully. Separate the product from the supernatant by medium-pressure liquid chromatography (preparative HPLC performed on a Biotage Isolera One using a SepaFlash® column with an UV detector) at last. The final mass of the product is 20.5 mg and the total yield is 19.1%. The product has been characterized by HR-MS and NMR spectroscopy.

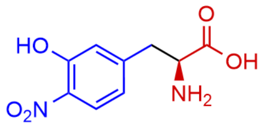

F11 (31.7%, 89.5 mg)

F11: (*S*)-3-hydroxy-4-nitrophenylalanine

Synthesis of (*S*)-3-hydroxy-4-nitrophenylalanine (**F11**) by lyophilized *E. coli* (PpLTA-RpTD) whole-cell catalyst. Prepare 1 L phosphate buffer ($Na_2HPO_4 \cdot 12H_2O$: 18 g, 50 mM; NaCl: 2.9 g, 50 mM; 1000 mL H_2O; pH 7.4), 5.5 mL 3-hydroxy-4-nitrobenzaldehyde stock solution (0.2298 g, 250 mM, dissolved by DMSO), 22 mL Gly stock solution (1.0313 g, 625 mM, dissolved by phosphate buffer), 42 mL L-Glu stock solution (2.8941 g, 468.8 mM, dissolved by phosphate buffer with 0.8 mL of 5M NaOH solution) in advance. Weigh 0.5 g lyophilized cell

E. coli BL21 (DE3)/pACYC-PpLTA-RpTD into a round-bottom flask, then add 15 mL phosphate buffer, 10 mL Gly stock solution, 20 mL L-Glu stock solution and 5 mL 3-hydroxy-4-nitrobenzaldehyde stock solution sequentially. Stir the mixture at room temperature (25 °C) for 12 h and quench the reaction with 2 mL of 5 M HCl solution. Then centrifuge the reaction at 13000 rpm for 10 min and collect the supernatant carefully. Separate the product from the supernatant by medium-pressure liquid chromatography (preparative HPLC performed on a Biotage Isolera One using a SepaFlash® column with an UV detector) at last. The final mass of the product is 89.5 mg and the total yield is 31.7%. The product has been characterized by HR-MS and NMR spectroscopy.

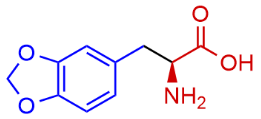

F12 (58.9%, 123.1 mg)

F12: (*S*)-3-(benzo[*d*][1,3]dioxol)alanine

Synthesis of (*S*)-3-(benzo[*d*][1,3]dioxol)alanine (**F12**) by lyophilized *E. coli* (PpLTA-RpTD) whole-cell catalyst. Prepare 1 L phosphate buffer ($Na_2HPO_4 \cdot 12H_2O$: 18 g, 50 mM; NaCl: 2.9 g, 50 mM; 1000 mL H_2O; pH 7.4), 4.2 mL benzo[*d*][1,3]dioxole-5-carbaldehyde stock solution (0.1600 g, 250 mM, dissolved by DMSO), 22 mL Gly stock solution (1.0313 g, 625 mM, dissolved by phosphate buffer), 42 mL L-Glu stock solution (2.8941 g, 468.8 mM, dissolved by phosphate buffer with 0.8 mL of 5M NaOH solution) in advance. Weigh 0.4 g lyophilized cell *E. coli* BL21 (DE3)/pACYC-PpLTA-RpTD into a round-bottom flask, then add 12 mL phosphate buffer, 8 mL Gly stock solution, 16 mL L-Glu stock solution and 4 mL benzo[*d*][1,3]dioxole-5-carbaldehyde stock solution sequentially. Stir the mixture at room temperature (25 °C) for 12 h and quench the reaction with 2 mL of 5 M HCl solution. Then centrifuge the reaction at 13000 rpm for 10 min and collect the supernatant carefully. Separate the product from the supernatant by medium-pressure liquid chromatography (preparative HPLC performed on a Biotage Isolera One using a SepaFlash® column with an UV detector) at last. The final mass of the product is 123.1 mg and the total yield is 58.9%. The product has been characterized by HR-MS and NMR spectroscopy.

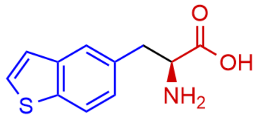

F13 (37.4%, 82.7 mg)

F13: (*S*)-3-(benzo[*b*]thiophen-5-yl)alanine

Synthesis of (*S*)-2-amino-3-(benzo[*b*]thiophen-5-yl)propanoic acid (**F13**) by lyophilized *E. coli* (PpLTA-RpTD) whole-cell catalyst. Prepare 1 L phosphate buffer ($Na_2HPO_4{\cdot}12H_2O$: 18 g, 50 mM; NaCl: 2.9 g, 50 mM; 1000 mL H_2O; pH 7.4), 4.1 mL benzo[*b*]thiophene-5-carbaldehyde stock solution (0.1670 g, 250 mM, dissolved by DMSO), 22 mL Gly stock solution (1.0313 g, 625 mM, dissolved by phosphate buffer), 42 mL L-Glu stock solution (2.8941 g, 468.8 mM, dissolved by phosphate buffer with 0.8 mL of 5M NaOH solution) in advance. Weigh 0.4 g lyophilized cell *E. coli* BL21 (DE3)/pACYC-PpLTA-RpTD into a round-bottom flask, then add 12 mL phosphate buffer, 8 mL Gly stock solution, 16 mL L-Glu stock solution and 4 mL benzo[*b*]thiophene-5-carbaldehyde stock solution sequentially. Stir the mixture at room temperature (25 °C) for 12 h and quench the reaction with 2 mL of 5 M HCl solution. Then centrifuge the reaction at 13000 rpm for 10 min and collect the supernatant carefully. Separate the product from the supernatant by medium-pressure liquid chromatography (preparative HPLC performed on a Biotage Isolera One using a SepaFlash® column with an UV detector) at last. The final mass of the product is 82.7 mg and the total yield is 37.4%. The product has been characterized by HR-MS and NMR spectroscopy.

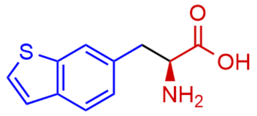

F14 (26.4%, 73.0 mg)

F14: (*S*)-3-(benzo[*b*]thiophen-6-yl)alanine

Synthesis of (*S*)-3-(benzo[*b*]thiophen-6-yl)alanine (**F14**) by lyophilized *E. coli* (PpLTA-RpTD) whole-cell catalyst. Prepare 1 L phosphate buffer ($Na_2HPO_4{\cdot}12H_2O$: 18 g, 50 mM; NaCl: 2.9 g, 50 mM; 1000 mL H_2O; pH 7.4), 5.1 mL benzo[*b*]thiophene-6-carbaldehyde stock solution (0.2086 g, 250 mM, dissolved by DMSO), 22 mL Gly stock solution (1.0313 g, 625 mM, dissolved by phosphate buffer), 42 mL L-Glu stock solution (2.8941 g, 468.8 mM, dissolved by phosphate buffer with 0.8 mL of 5M NaOH solution) in advance. Weigh 0.5 g lyophilized cell

E. coli BL21 (DE3)/pACYC-PpLTA-RpTD into a round-bottom flask, then add 15 mL phosphate buffer, 10 mL Gly stock solution, 20 mL L-Glu stock solution and 5 mL benzo[*b*]thiophene-6-carbaldehyde stock solution sequentially. Stir the mixture at room temperature (25 °C) for 12 h and quench the reaction with 2 mL of 5 M HCl solution. Then centrifuge the reaction at 13000 rpm for 10 min and collect the supernatant carefully. Separate the product from the supernatant by medium-pressure liquid chromatography (preparative HPLC performed on a Biotage Isolera One using a SepaFlash® column with an UV detector) at last. The final mass of the product is 73.0 mg and the total yield is 26.4%. The product has been characterized by HR-MS and NMR spectroscopy.

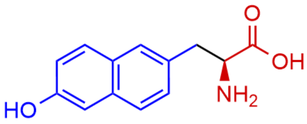

F15 (5.5%, 15.9 mg)

F15: (*S*)-3-(6-hydroxynaphthalen)alanine

Synthesis of (*S*)-3-(6-hydroxynaphthalen)alanine (**F15**) by lyophilized *E. coli* (PpLTA-RpTD) whole-cell catalyst. Prepare 1 L phosphate buffer ($Na_2HPO_4 \cdot 12H_2O$: 18 g, 50 mM; NaCl: 2.9 g, 50 mM; 1000 mL H_2O; pH 7.4), 5.5 mL 6-hydroxynaphthalene-2-carbaldehyde stock solution (0.2368 g, 250 mM, dissolved by DMSO), 22 mL Gly stock solution (1.0313 g, 625 mM, dissolved by phosphate buffer), 42 mL L-Glu stock solution (2.8941 g, 468.8 mM, dissolved by phosphate buffer with 0.8 mL of 5M NaOH solution) in advance. Weigh 0.5 g lyophilized cell *E. coli* BL21 (DE3)/pACYC-PpLTA-RpTD into a round-bottom flask, then add 15 mL phosphate buffer, 10 mL Gly stock solution, 20 mL L-Glu stock solution and 5 mL 6-hydroxynaphthalene-2-carbaldehyde stock solution sequentially. Stir the mixture at room temperature (25 °C) for 12 h and quench the reaction with 2 mL of 5 M HCl solution. Then centrifuge the reaction at 13000 rpm for 10 min and collect the supernatant carefully. Separate the product from the supernatant by medium-pressure liquid chromatography (preparative HPLC performed on a Biotage Isolera One using a SepaFlash® column with an UV detector) at last. The final mass of the product is 15.9 mg and the total yield is 5.5%. The product has been characterized by HR-MS and NMR spectroscopy.

Characterization of ncAA products

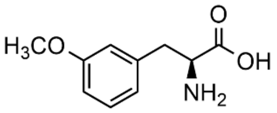

F1

(*S*)-3-methoxyphenylalanine (**F1**): 38.3% yield, $[\alpha]^{20}_{D}$ = -22 (*c* = 0.05 in H_2O), ^1H NMR (400 MHz, D_2O + NaOH) δ 7.36 (t, *J* = 7.9 Hz, 1H), 6.95 (m, 3H), 4.00 (t, *J* = 6.7 Hz, 1H), 3.84 (s, 3H), 3.27 (dd, *J* = 14.5, 4.9 Hz, 1H), 3.10 (dd, *J* = 14.5, 8.2 Hz, 1H) ppm. ^{13}C NMR (151 MHz, D_2O + NaOH) δ 173.7, 159.2, 136.8, 130.3, 122.0, 114.8, 113.2, 55.8, 55.3, 36.3 ppm. HRMS (ESI) (*m/z*) for $[M+H]^+$ $C_{10}H_{13}ClNO_3$ calculated 196.0968, observed 196.0971.

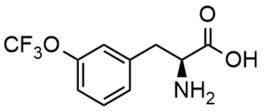

F2

(*S*)-3-(trifluoromethoxy)phenylalanine (**F2**): 33.9% yield, $[\alpha]^{20}_{D}$ = -4 (*c* = 0.05 in H_2O), ^1H NMR (400 MHz, D_2O + NaOH) δ 7.47 (t, *J* = 7.8 Hz, 1H), 7.28 (m, 3H), 3.90 (dd, *J* = 7.7, 5.3 Hz, 1H), 3.25 (dd, *J* = 14.4, 5.3 Hz, 1H), 3.10 (dd, *J* = 14.3, 7.7 Hz, 1H) ppm. ^{13}C NMR (126 MHz, D_2O + NaOH) δ 174.8, 148.6, 137.6, 129.9, 127.5, 121.3, 119.8 ($^1J_{C\text{-}F}$ = 255.8 Hz), 119.4, 55.60, 36.44. ^{19}F NMR (471 MHz, D_2O + NaOH) δ -57.7 ppm. HRMS (ESI) (*m/z*) for $[M+H]^+$ $C_{11}H_{11}F_3NO_5$ calculated 250.0686, observed 250.0685.

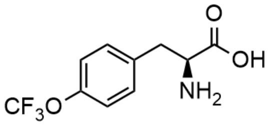

F3

(*S*)-4-(trifluoromethoxy)phenylalanine (**F3**): 39.3% yield, $[\alpha]^{20}_{D}$ = -6 (*c* = 0.05 in H_2O), ^1H NMR (400 MHz, D_2O + NaOH) δ 7.35 (d, *J* = 8.6 Hz, 2H), 7.30 (d, *J* = 8.5 Hz, 2H), 3.66 (t, *J* = 6.4 Hz, 1H), 3.10 (dd, *J* = 14.0, 5.3 Hz, 1H), 2.97 (dd, *J* = 13.9, 7.4 Hz, 1H) ppm. ^{13}C NMR (126 MHz, D_2O + NaOH) δ 178.7, 147.4, 135.7, 130.3, 130.3, 120.7, 120.7,119.8 ($^1J_{C\text{-}F}$ = 255.8 Hz), 56.4, 38.0 ppm. ^{19}F NMR (471 MHz, D_2O + NaOH) δ -57.9 ppm. HRMS (ESI) (*m/z*) for $[M+H]^+$ $C_{11}H_{11}F_3NO_5$ calculated 250.0686, observed 250.0689.

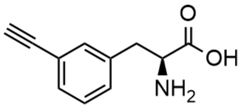

F4

(*S*)-3-ethynylphenylalanine (**F4**): 65.6% yield, $[\alpha]^{20}_{D}$ = -4 (*c* = 0.05 in H_2O), ^1H NMR (400 MHz, methanol-d_4) δ 7.46 (d, *J* = 1.6 Hz, 1H), 7.39 (qd, *J* = 4.3, 3.8, 1.4 Hz, 1H), 7.34 (m, 2H), 3.78 (dd, *J* = 8.7, 4.5 Hz, 1H), 3.50 (s, 1H), 3.29 (dd, *J* = 14.6, 5.2 Hz, 1H), 3.01 (dd, *J* = 14.6, 8.7 Hz, 1H) ppm. ^{13}C NMR (151 MHz, methanol-d_4) δ 173.5, 137.8, 134.0, 132.0, 130.9, 130.1, 124.4, 84.2, 79.0, 57.3, 37.9 ppm. HRMS (ESI) (*m/z*) for $[M+H]^+$ $C_{11}H_{11}F_3NO_5$ calculated 190.0863, observed 190.0864.

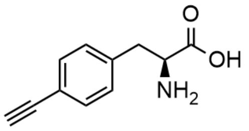

F5

(*S*)-4-ethynylphenylalanine (**F5**): 38.1% yield, $[\alpha]^{20}_{D}$ = -10 (*c* = 0.05 in H_2O), ^{1}H NMR (400 MHz, methanol-d_4) δ 7.40 (d, *J* = 7.8 Hz, 2H), 7.27 (d, *J* = 7.8 Hz, 2H), 3.61 (dd, *J* = 8.3, 4.7 Hz, 1H), 3.43 (s, 1H), 3.19 (dd, *J* = 14.0, 4.7 Hz, 1H), 2.91 (dd, *J* = 14.0, 8.2 Hz, 1H) ppm. ^{13}C NMR (151 MHz, methanol-d_4) δ 177.6, 139.7, 133.2, 133.2, 130.6, 130.6, 122.2, 84.3, 78.5, 58.1, 40.4 ppm. HRMS (ESI) (*m/z*) for $[M+H]^+$ $C_{11}H_{11}F_3NO_5$ calculated 190.0863, observed 190.0862.

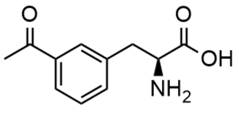

F6

(*S*)-3-acetylphenylalanine (**F6**): 70.4% yield, $[\alpha]^{20}_{D}$ = -23 (*c* = 0.05 in H_2O), ^{1}H NMR (400 MHz, D_2O) δ 7.95 (dt, *J* = 7.5, 1.6 Hz, 1H), 7.89 (d, *J* = 2.2 Hz, 1H), 7.57 (m, 2H), 4.04 (dd, *J* = 7.6, 5.5 Hz, 1H), 3.34 (dd, *J* = 14.4, 5.6 Hz, 1H), 3.22 (dd, *J* = 14.6, 7.6 Hz, 1H), 2.67 (s, 3H) ppm. ^{13}C NMR (126 MHz, D_2O) δ 203.3, 173.0 136.5, 135.2, 134.3, 129.0, 128.7, 127.4, 55.3, 35.6, 25.8 ppm. HRMS (ESI) (*m/z*) for $[M+H]^+$ $C_{11}H_{11}F_3NO_5$ calculated 208.0968, observed 208.0968.

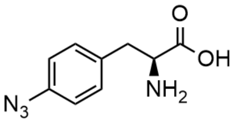

F7

(*S*)-4-azidophenylalanine (**F7**): 50.9% yield, $[\alpha]^{20}_{D}$ = -10 (*c* = 0.05 in H_2O), ^{1}H NMR (400 MHz, D_2O + NaOH) δ 7.29 (d, *J* = 8.0 Hz, 2H), 7.08 (d, *J* = 8.0 Hz, 2H), 3.64 (t, *J* = 6.5 Hz, 1H), 3.05 (dd, *J* = 14.0, 5.6 Hz, 1H), 2.93 (dd, *J* = 14.0, 7.3 Hz, 1H) ppm. ^{13}C NMR (126 MHz, D_2O + NaOH) δ 179.4, 138.4, 133.8, 130.7, 130.7, 119.0, 119.0, 56.9, 38.6 ppm. HRMS (ESI) (*m/z*) for $[M+H]^+$ $C_{11}H_{11}F_3NO_5$ calculated 207.0877, observed 207.0877.

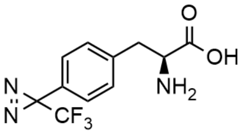

F8

(*S*)-4-(3-(trifluoromethyl)-3*H*-diazirin)phenylalanine (**F8**): 67.5% yield, $[\alpha]^{20}_{D}$ = -9 (*c* = 0.05 in H_2O), ^{1}H NMR (400 MHz, D_2O + NaOH) δ 7.34 (d, *J* = 8.0 Hz, 2H), 7.26 (d, *J* = 8.0 Hz, 2H), 3.52 (t, *J* = 6.5 Hz, 1H), 3.02 (dd, *J* = 13.7, 5.6 Hz, 1H), 2.88 (dd, *J* = 13.6, 7.3 Hz, 1H) ppm. ^{13}C NMR (126 MHz, D_2O + NaOH) δ 181.2, 139.7, 129.4, 129.4, 126.4, 126.1, 126.1, 121.6 ($^{1}J_{C\text{-}F}$ = 274.7 Hz), 56.7, 39.7, 27.9 ($^{2}J_{C\text{-}F}$ = 40.3 Hz) ppm. ^{19}F NMR (471 MHz, D_2O + NaOH) δ -65.6 ppm. HRMS (ESI) (*m/z*) for $[M+H]^+$ $C_{11}H_{11}F_3NO_5$ calculated 274.0798, observed 274.0796.

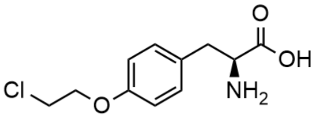

F9

(*S*)-4-(2-chloroethoxy)phenylalanine (**F9**): 28.8% yield, $[\alpha]^{20}_{D}$ = -12 (*c* = 0.05 in H_2O), ^{1}H NMR

(400 MHz, D_2O + NaOH) δ 7.25 (d, J = 8.1 Hz, 2H), 7.02 (d, J = 8.1 Hz, 2H), 4.36 (t, J = 5.1 Hz, 2H), 3.92 (t, J = 4.8 Hz, 2H), 3.73 (t, J = 6.6 Hz, 1H), 3.10 (dd, J = 14.3, 5.2 Hz, 1H), 2.96 (dd, J = 14.3, 7.5 Hz, 1H) ppm. ^{13}C NMR (126 MHz, D_2O + NaOH) δ 177.3, 156.2, 130.2, 130.2, 129.3, 114.9, 114.9, 68.3, 56.2, 42.3, 36.9 ppm. HRMS (ESI) (*m/z*) for $[M+H]^+$ $C_{11}H_{11}F_3NO_5$ calculated 244.0735, observed 244.0733.

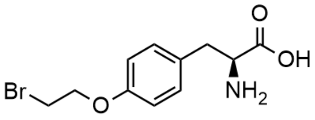

F10

(*S*)-4-(2-bromoethoxy)phenylalanine (**F10**): 19.1% yield, $[\alpha]^{20}_D$ = -13 (c = 0.05 in H_2O), ^1H NMR (400 MHz, D_2O + NaOH) δ 7.23 (d, J = 8.1 Hz, 2H), 7.01 (d, J = 8.2 Hz, 2H), 4.42 (t, J = 5.4 Hz, 2H), 3.76 (t, J = 5.4 Hz, 2H), 3.54 (t, J = 6.4 Hz, 1H), 2.98 (dd, J = 13.9, 5.6 Hz, 1H), 2.86 (dd, J = 13.9, 7.2 Hz, 1H) ppm. ^{13}C NMR (126 MHz, D_2O + NaOH) δ 181.0, 156.3, 131.1, 130.6, 115.3, 68.6, 57.16, 39.1, 30.5 ppm. HRMS (ESI) (*m/z*) for $[M+H]^+$ $C_{11}H_{11}F_3NO_5$ calculated 288.0230, observed 288.0230.

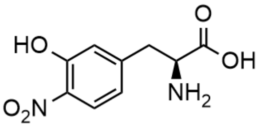

F11

(*S*)-3-hydroxy-4-nitrophenylalanine (**F11**): 31.7% yield, $[\alpha]^{20}_D$ = -14 (c = 0.05 in H_2O), ^1H NMR (400 MHz, D_2O + NaOH) δ 7.91 (d, J = 8.7 Hz, 1H), 6.73 (s, 1H), 6.49 (d, J = 8.7 Hz, 1H), 3.98 (dd, J = 8.8, 4.8 Hz, 1H), 3.21 (dd, J = 14.4, 4.7 Hz, 1H), 2.97 (dd, J = 14.4, 8.9 Hz, 1H) ppm. ^{13}C NMR (151 MHz, D_2O) δ 173.8, 163.7, 144.1, 135.9, 126.9, 124.9, 115.0, 55.3, 36.4 ppm. HRMS (ESI) (*m/z*) for $[M+H]^+$ $C_{11}H_{11}F_3NO_5$ calculated 227.0662, observed 227.0661.

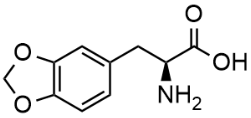

F12

(*S*)-3-(benzo[*d*][1,3]dioxol)alanine (**F12**): 58.9% yield, $[\alpha]^{20}_D$ = -8 (c = 0.05 in H_2O), ^1H NMR (400 MHz, D_2O + NaOH) δ 6.86 (d, J = 7.9 Hz, 1H), 6.82 (s, 1H), 6.76 (d, J = 8.1 Hz, 1H), 5.96 (s, 2H), 3.60 (t, J = 6.5 Hz, 1H), 3.00 (dd, J = 14.0, 5.5 Hz, 1H), 2.87 (dd, J = 14.0, 7.4 Hz, 1H) ppm. ^{13}C NMR (126 MHz, D_2O + NaOH) δ 179.5, 147.2, 145.9, 130.9, 122.6, 109.6, 108.5, 100.9, 57.0, 38.9 ppm. HRMS (ESI) (*m/z*) for $[M+H]^+$ $C_{11}H_{11}F_3NO_5$ calculated 210.0761, observed 210.0763.

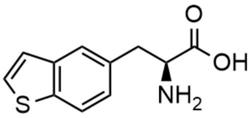

F13

(*S*)-3-(benzo[*b*]thiophen-5-yl)alanine (**F13**): 37.4% yield, $[\alpha]^{20}_D$ = -10 (c = 0.05 in H_2O), ^1H

NMR (400 MHz, D_2O + NaOH) δ 7.86 (m, 2H), 7.58 (d, J = 5.5 Hz, 1H), 7.42 (d, J = 5.5 Hz, 1H), 7.31 (d, J = 8.2 Hz, 1H), 3.59 (t, J = 6.5 Hz, 1H), 3.14 (dd, J = 13.7, 5.5 Hz, 1H), 2.99 (dd, J = 13.7, 7.4 Hz, 1H) ppm. ^{13}C NMR (126 MHz, D_2O + NaOH) δ 181.7, 139.7, 138.2, 134.3, 126.7, 126.0, 123.7, 123.5, 122.9, 57.4, 40.4 ppm. HRMS (ESI) (*m/z*) for $[M+H]^+$ $C_{11}H_{11}F_3NO_5$ calculated 222.0583, observed 222.0580.

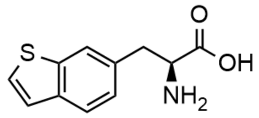

F14

(*S*)-3-(benzo[*b*]thiophen-6-yl)alanine (**F14**): 26.4% yield, $[\alpha]^{20}_D$ = -11 (c = 0.05 in H_2O), ^1H NMR (400 MHz, D_2O + NaOH) δ 7.93 (d, J = 8.3 Hz, 1H), 7.77 (s, 1H), 7.61 (d, J = 5.5 Hz, 1H), 7.42 (d, J = 5.5 Hz, 1H), 7.29 (d, J = 8.3 Hz, 1H), 3.56 (t, J = 6.5 Hz, 1H), 3.12 (dd, J = 13.6, 5.6 Hz, 1H), 2.97 (dd, J = 13.6, 7.3 Hz, 1H) ppm. ^{13}C NMR (126 MHz, D_2O + NaOH) δ 182.3, 139.8, 137.7, 134.4, 127.39, 125.9, 124.1, 123.8, 122.5, 57.5, 40.6 ppm. HRMS (ESI) (*m/z*) for $[M+H]^+$ $C_{11}H_{11}F_3NO_5$ calculated 222.0583, observed 222.0584.

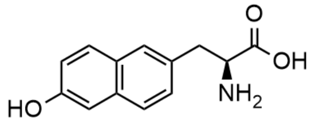

F15

(*S*)-3-(6-hydroxynaphthalen)alanine (**F15**): 5.5% yield, $[\alpha]^{20}_D$ = -34 (c = 0.05 in H_2O), ^1H NMR (400 MHz, D_2O + NaOH) δ 7.67 (d, J = 8.9 Hz, 1H), 7.57 (d, J = 8.9 Hz, 2H), 7.24 (d, J = 8.0 Hz, 1H), 7.00 (d, J = 8.8 Hz, 1H), 6.89 (s, 1H), 3.55 (t, J = 5.7 Hz, 1H), 3.09 (dd, J = 13.7, 5.0 Hz, 1H), 2.89 (dd, J = 13.6, 7.9 Hz, 1H) ppm. ^{13}C NMR (126 MHz, D_2O + NaOH) δ 181.9, 163.4, 134.3, 129.8, 128.3, 127.1, 127.0, 125.4, 124.9, 123.4, 109.3, 56.8, 40.1 ppm. HRMS (ESI) (*m/z*) for $[M+H]^+$ $C_{11}H_{11}F_3NO_5$ calculated 232.0968, observed 232.0969.

Supplementary method 3. Substrate scope of aromatic ncAAs biosynthesis in culture.

Four different LTAs and RpTD co-expression plasmids were chemically transformed into *E. coli* RARE (DE3) competent cells. A total of 500 μL of LB medium containing 35 $\mu g \cdot mL^{-1}$ chloramphenicol (Cm) was added to each well of a 96-deep well plate, and 10 μL of overnight cultured bacterial solution was inoculated into the plate. The plate was incubated on an orbital shaker at 37 °C and 800 rpm for 2 hours, or until the optical density at 600 nm (OD_{600}) reached 0.8-1.0. Meanwhile, 150 μL of 1 M IPTG was added to 25 mL of LB medium containing 35 $\mu g \cdot mL^{-1}$ Cm to prepare an IPTG matric solution. From this, 50 μL was added to each well of the 96-well plate. The culture temperature was then lowered to 30 °C. Next, 15 μL of 2 M Gly and 12 μL of 1 mM PLP were added to each well. 12 μL of aromatic aldehyde substrate, which

was dissolved in DMSO at 50 mM concentration, was added to the corresponding wells, with three parallel replicates for each substrate.

The culture was further incubated at 30 °C, 800 rpm for 12 hours. After the reaction, the samples were diluted 5-fold with 50% methanol (MeOH) and filtered through a membrane for UPLC-MS analysis. The analysis was performed in SIM mode to detect the molecular weight of the product via ESI $[M+H]^+$. The peak areas for each product were used to calculate the concentration, and the conversion rate was determined using the standard curve of the corresponding product.

Supplementary method 4. Expression, fluorescence measurement and purification of sfGFP

Expression and Fluorescence Measurement of sfGFP. Plasmid pET22b-sfGFP(Y151TAG) was transformed into *E. coli* RARE (DE3), and the strain was prepared as competent cells. The co-expression plasmid pACYC-CsLTA-RpTD, along with plasmids for expressing various aaRS/tRNA pairs, were co-transformed into *E. coli* RARE (DE3)/pET22b-sfGFP(Y151TAG) competent cells. For co-transformation with pCDF as the aaRS/tRNA vector, the cells were plated on agar containing 35 $\mu g \cdot mL^{-1}$ chloramphenicol (Cm), 100 $\mu g \cdot mL^{-1}$ ampicillin (Amp), and 50 $\mu g \cdot mL^{-1}$ kanamycin (Kan). For co-transformation with pUltra as the aaRS/tRNA vector, the cells were plated on agar containing 35 $\mu g \cdot mL^{-1}$ Cm, 100 $\mu g \cdot mL^{-1}$ Amp, and 100 $\mu g \cdot mL^{-1}$ streptomycin (Sm). After incubation, the three-plasmid co-expression strains were obtained.

Single colonies were selected and inoculated into LB medium containing the appropriate antibiotics. After overnight culture, the cells were transferred to fresh LB medium at a 1% inoculum, supplemented with the corresponding antibiotics, and cultured at 37 °C, 220 rpm until the OD_{600} reached 0.8-1.0. To induce expression, 1 mM IPTG was added and the temperature was reduced to 30 °C. For the reaction, chemicals were added to the medium at final concentration of 1 mM aromatic aldehyde substrates, 50 mM Gly, and 20 μM PLP, and the culture was continued at 30 °C, 230 rpm for 24 hours.

After the expression of sfGFP, 1 mL of the *E. coli* culture was taken, and the bacterial concentration was measured using a UV spectrophotometer. The supernatant was removed by centrifugation, and the bacterial pellet was washed twice with PBS (pH 7.5). The cells were

then resuspended in 1 mL PBS (pH 7.5). The fluorescence intensity (Ex: 485 nm; Em: 528 nm) of the resuspended cells was measured using a plate reader with 200 μL of the suspension. The ratio of fluorescence intensity to bacterial concentration (RFU/OD_{600}) was used to quantify the sfGFP expression level of the recombinant strains.

A negative control group, cultured without aromatic aldehyde substrates but supplemented only with Gly and PLP, and a positive control group, supplemented with 1 mM aromatic ncAAs, were used for comparison.

Purification of sfGFP. A single colony of the tri-plasmid strain was cultured overnight in LB medium containing the three antibiotics. The culture was then transferred to 500 mL of LB medium containing the corresponding antibiotics at a 1% inoculum rate. Strain culture and sfGFP expression were performed as described above.

Afterward, the bacteria were harvested by centrifugation at 4 °C, 10,000 rpm for 10 minutes. The cell pellet was resuspended in lysis buffer (50 mM Tris, pH 8.0, 250 mM NaCl) and lysed using a high-pressure homogenizer. The lysate was centrifuged at 4 °C, 18,000 rpm for 30 minutes. The supernatant was then loaded three times onto a Ni-NTA agarose purification column, pre-equilibrated with lysis buffer. The column was washed with 10 column volumes of wash buffer (50 mM Tris, pH 8.0, 250 mM NaCl, 25 mM imidazole) and then eluted with 5 column volumes of elution buffer (50 mM Tris, pH 8.0, 250 mM NaCl, 250 mM imidazole) to obtain the target sfGFP. The protein solution, containing a high concentration of imidazole, was dialyzed against dialysis buffer (50 mM Na_2HPO_4, pH 7.5, 50 mM NaCl). After dialysis, the protein solution was quickly frozen in liquid nitrogen and stored at -80 °C. The identity of the purified protein was confirmed by SDS-PAGE and mass spectrometry (MS).

Supplementary method 5. Macrocyclic peptide biosynthesis and analysis.

Plasmid pET22b-Npu with the TAG mutant, plasmid pACYC-CsLTA-RpTD or pCDF-CsLTA-RpTD, and plasmids for expressing various aaRS/tRNA pairs were co-transformed into *E. coli* RARE (DE3) competent cells. The transformed cells were plated on agar containing three antibiotics (35 μg·mL^{-1} Cm, 100 μg mL^{-1} Amp, and 100 μg mL^{-1} Sm) to select engineered strains capable of site-specific incorporation of biosynthetic ncAAs into the desired macrocycle via the *Npu* intein. Single colonies were selected and inoculated into LB medium containing

the three antibiotics, and the culture was grown overnight. The culture was then transferred at a 1% inoculum rate to a larger volume of medium. When the OD_{600} reached 0.8-1.0, the culture temperature was reduced to 30 °C. IPTG was added to a final concentration of 1 mM, and L-arabinose was added to a final concentration of 0.2%.

For the positive control group, ncAAs were added at a final concentration of 1 mM. For the experimental groups, the reaction mixture were added to the medium to final concentration at 1 mM aromatic aldehyde substrates, 50 mM Gly, and 20 μM PLP. All cultures were then incubated at 30 °C, shaking at 230 rpm for 24 hours.

After incubation, the cells were harvested by centrifugation, and the pellet was resuspended in 1% acetonitrile. The suspension was sonicated for 20-30 minutes. Following a centrifugation step at 13,000 rpm for 5 minutes, the supernatant was filtered and analyzed by LC-MS using single ion recording (SIR) in positive mode, the measure method detail was in General information.

Supplementary method 6. Expression and purification of pAzF-containing antibody fragments.

To increase the expression of target protein, a commercial plasmid pEVOL-pAzFRS-$tRNA^{Tyr}_{CUA}$ with double-copy pAzFRS gene was selected for pAzF insertion, containing P15A replicative origin and Cm resistance marker. CsLTA and RpTD co-expression gene cassette was cloned into pCDFDuet-1 vector containing CDF replicative origin and Sm resistance marker by Gibson assembly. Different antibody fragment expression plasmids with TAG mutation attached to pET22b were co-transformed with pCDF-CsLTA-RpTD and pEVOL-pAzFRS-$tRNA^{Tyr}_{CUA}$ into *E. coli* RARE (DE3). Three resistant plate screening (35 μg·mL^{-1} Cm, 100 μg·mL^{-1} Amp and 100 μg·mL^{-1} Sm) was performed to obtain engineered strains capable of site-specific insertion of biosynthetic pAzF into recombinant proteins. Single colonies were selected to LB medium containing three antibiotics, cultured overnight and inoculated at 1% rate to expanded culture. When the OD_{600} reached to 0.8-1.0, IPTG with a final concentration of 1 mM and L-arabinose with a final concentration of 0.2% were added to the medium and temperature dropped to 30 °C. The reaction mixture at final concentration of 1 mM *p*-azidebenzaldehyde, 50 mM Gly and 20 μM PLP was added into medium and cell was continued for 24 h at 30 °C, 230 rpm. After

harvested by centrifugation, proteins were purified on Ni-NTA unionrose 6FF purification column following the protocol of sfGFP purification.

Supplementary method 7. Protein conjugation

Fab-A121pAzF (1 mg/mL) in PBS (pH 7.4) was mixed with DBCO-AF488 (5.0 equiv.) and incubated at 25 °C for 7h. Upon completion, the excess dye was removed by 10 kDa Amicon Ultra centrifugal filter (Millipore), and the protein concentration was determined by measuring the absorbance at 280 nm and BCA Protein Assay (Pierce™).

Supplementary method 8. Confocal imaging

SK-Br-3 were seeded in 20 mm glass bottom dish (NEST) and allowed to grow to ~70% confluency in 20% FBS-DMEM with 1% Pen-Strep at 37 °C, 5% CO_2. MDA-MB-468 cells were seeded in 20 mm glass bottom dish and allowed to grow to ~70% confluency in 10% FBS-L-15 with 1% Pen-Strep at 37 °C, 100% air. SK-Br-3 and MDA-MB-468 cells were washed with PBS (1 mLx1) and blocked with PBS/2% FBS (1 mL) for 1 h at 37 °C. Cells were washed with PBS (1 mLx1), and then incubated with Fab-A121pAzF-AF488 conjugate (3 μM) in PBS at 37 °C for 1 h and Hoechst 33342 (2 μg/mL) at 37 °C for 10 min, respectively. Cells were washed with PBS (1 mLx3) and observed under ZEISS LSM 900 confocal fluorescence microscope (DAPI channel and AF488 channel).

NMR spectra

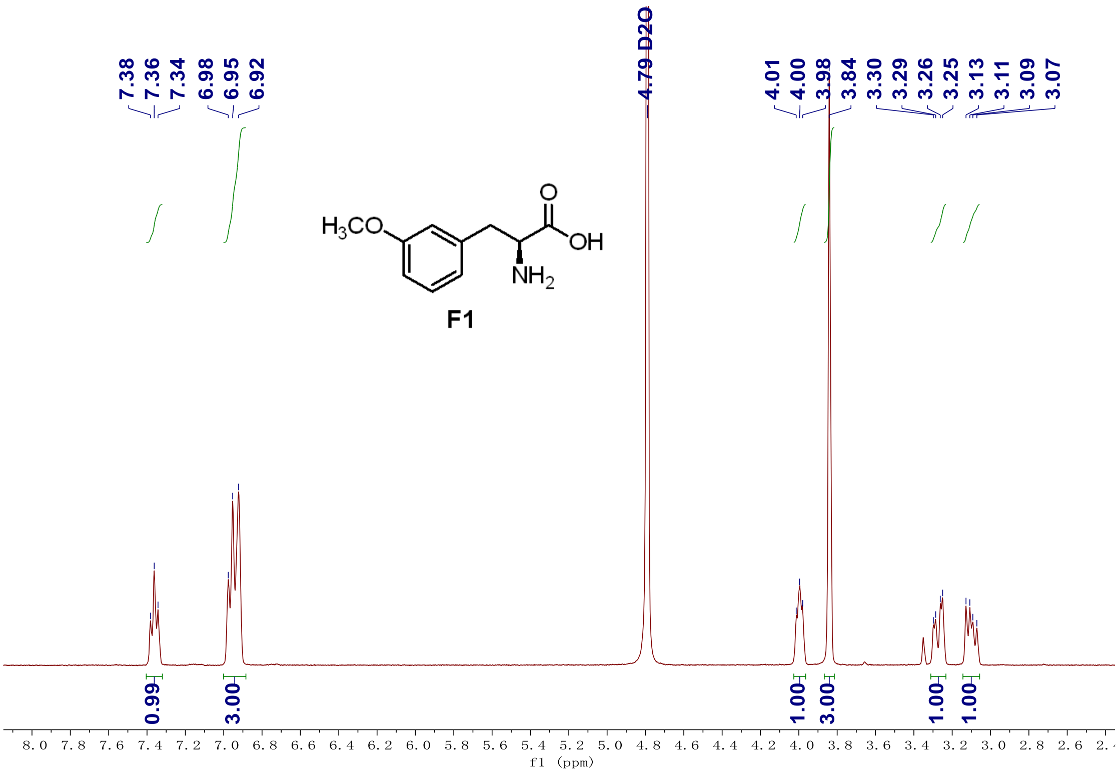

Supplementary Figure 26 ^{1}H NMR spectrum of **F1** in D_2O with NaOH.

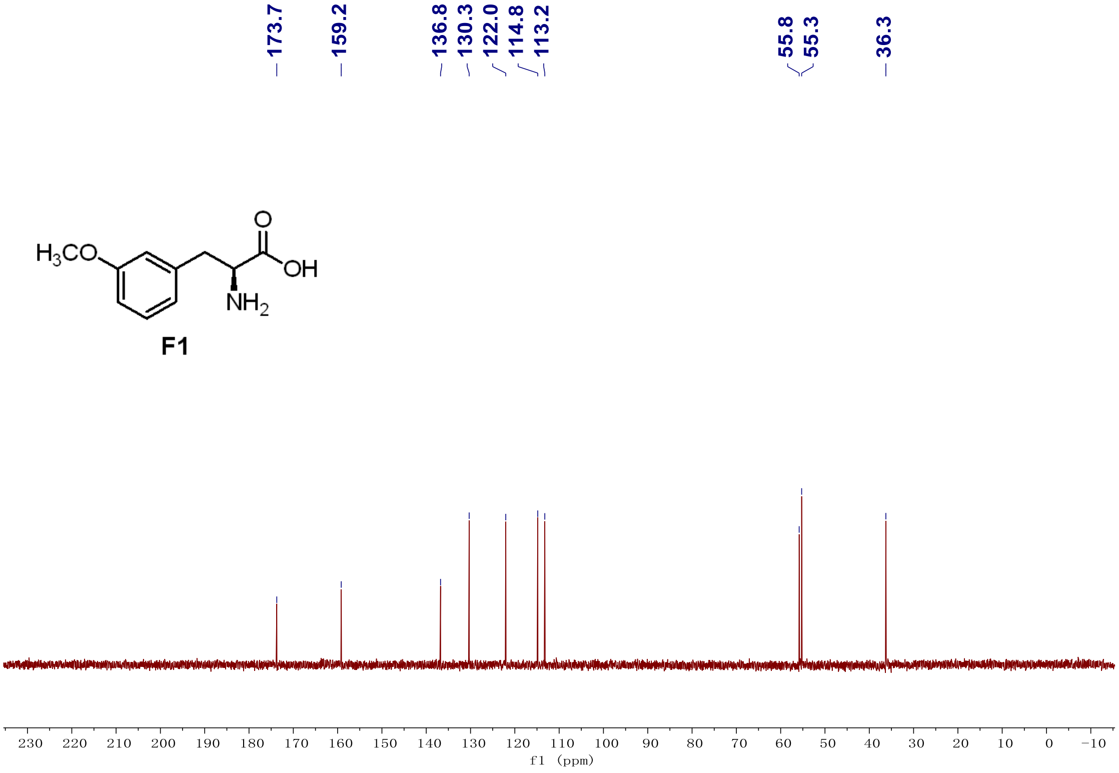

Supplementary Figure 27 ^{13}C NMR spectrum of **F1** in D_2O with NaOH.

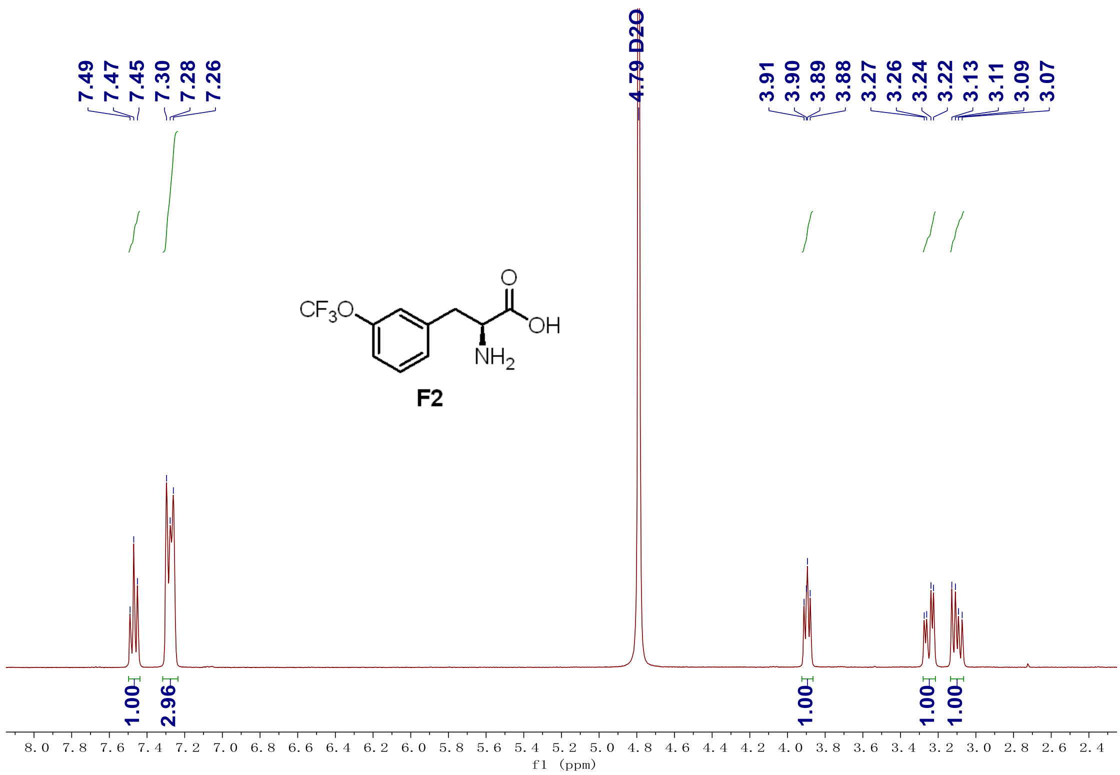

Supplementary Figure 28 ^{1}H NMR spectrum of **F2** in D_2O with NaOH.

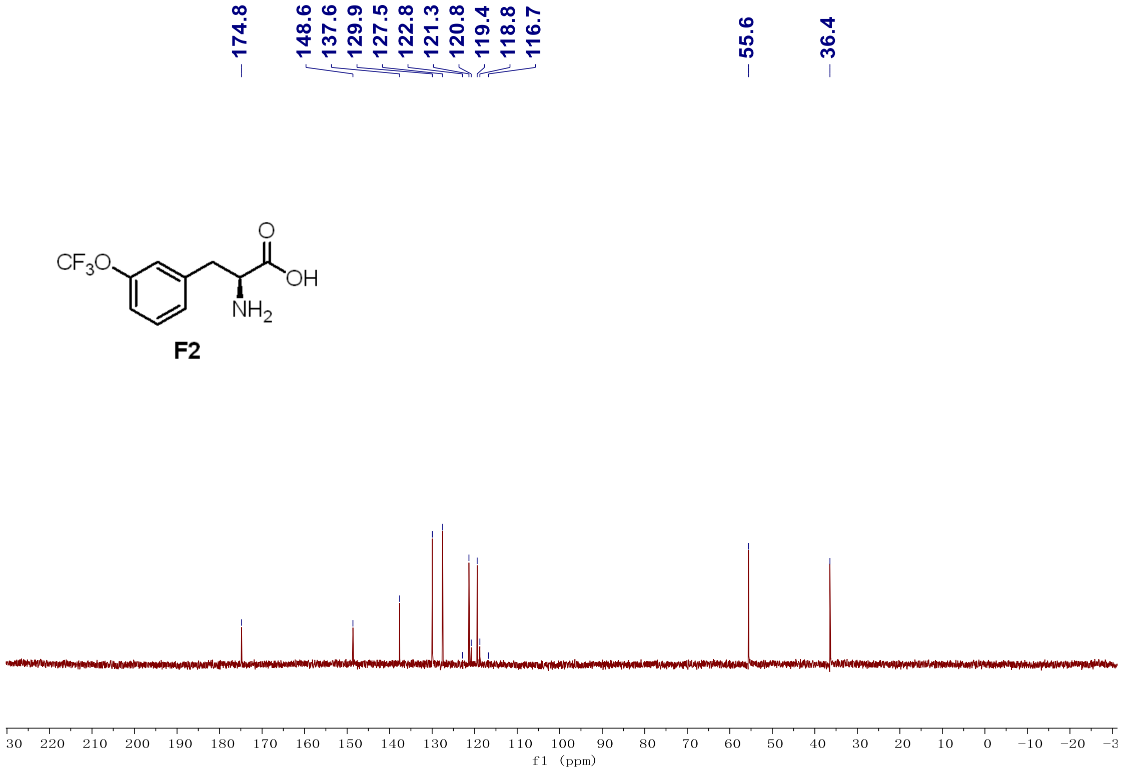

Supplementary Figure 29 ^{13}C NMR spectrum of **F2** in D_2O with NaOH.

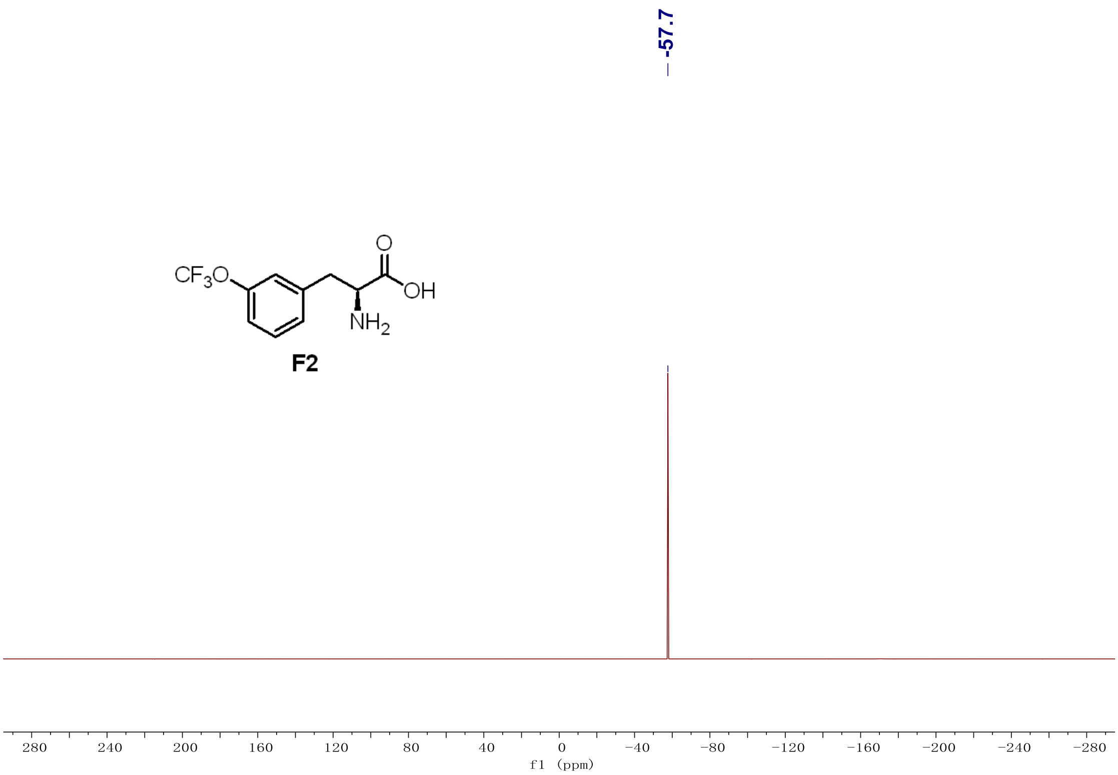

Supplementary Figure 30 ^{19}F NMR spectrum of **F2** in D_2O with NaOH.

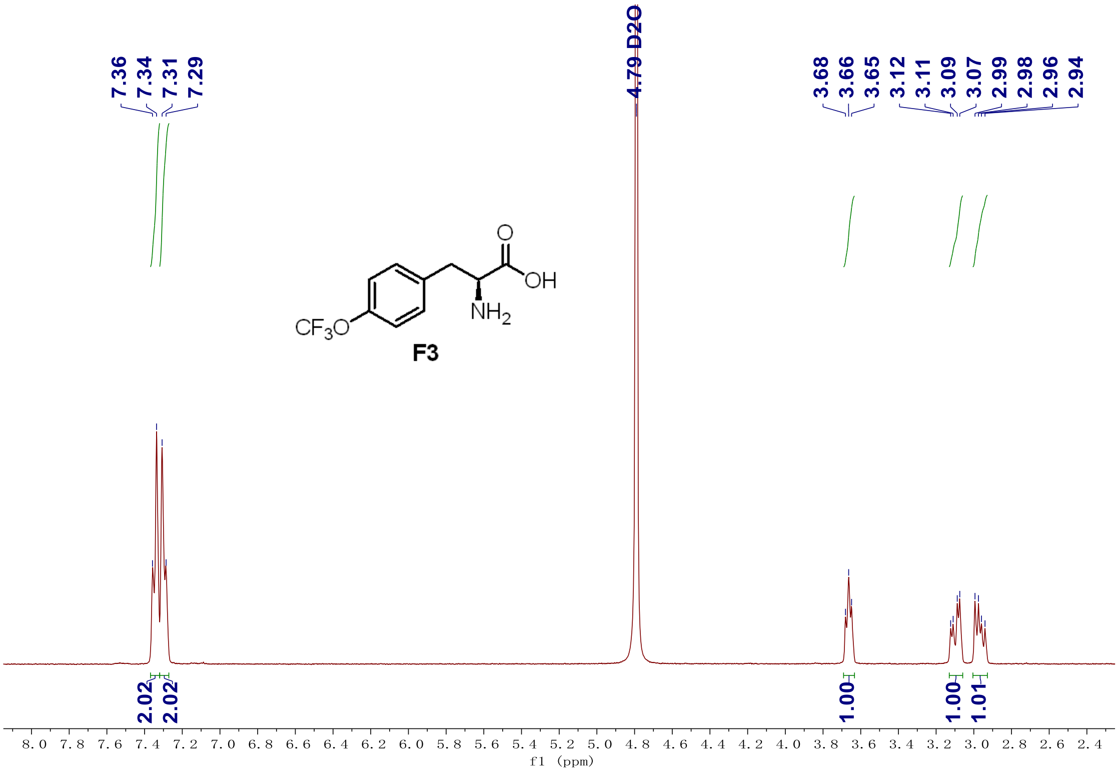

Supplementary Figure 31 ^{1}H NMR spectrum of **F3** in D_2O with NaOH.

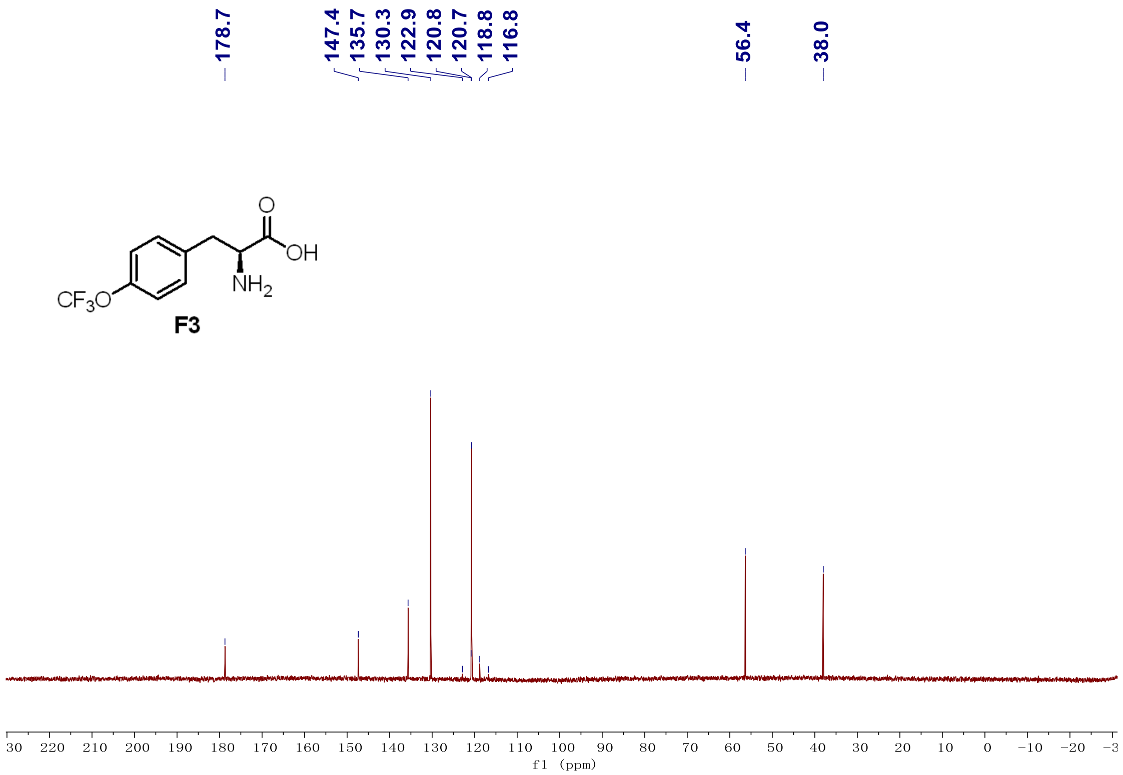

Supplementary Figure 32 ^{13}C NMR spectrum of **F3** in D_2O with NaOH.

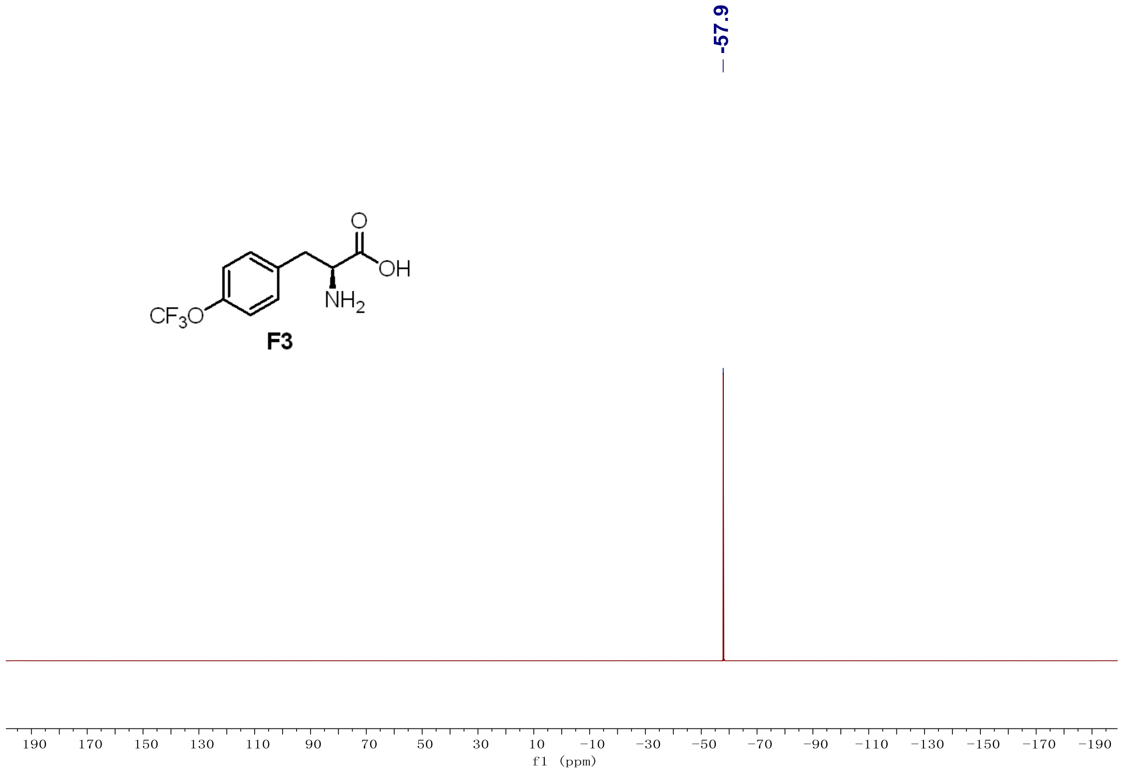

Supplementary Figure 33 ^{19}F NMR spectrum of **F3** in D_2O with NaOH.

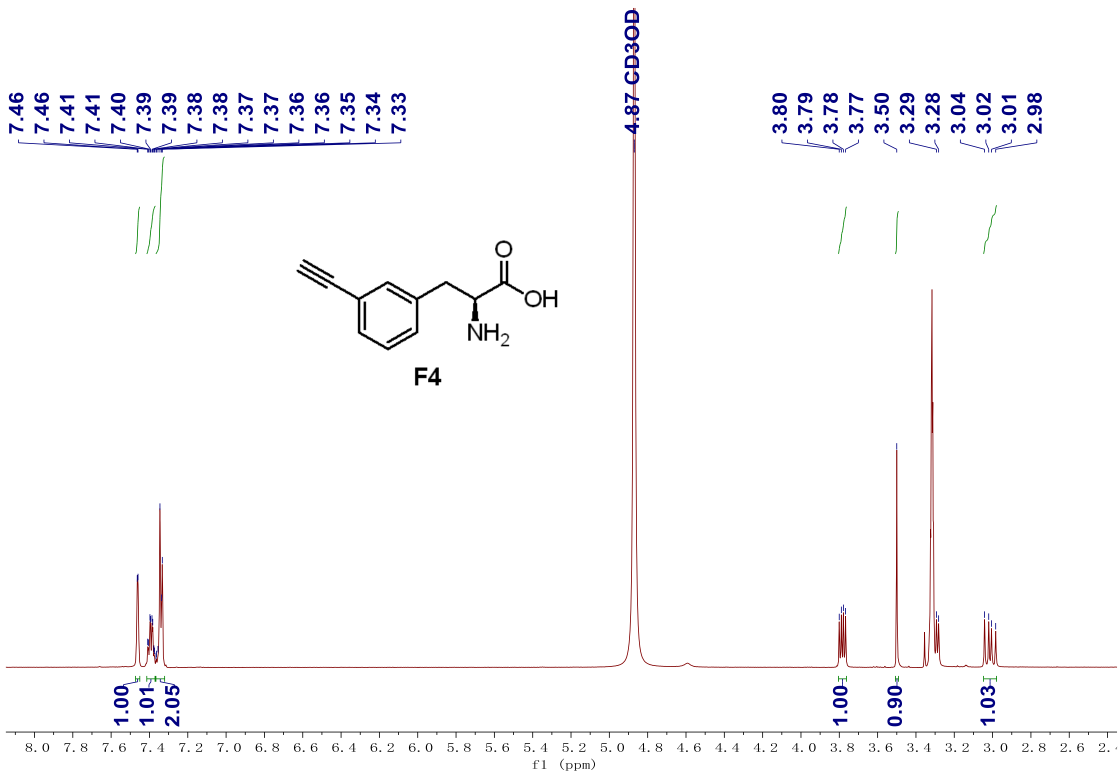

Supplementary Figure 34 ^{1}H NMR spectrum of **F4** in methanol-d_4.

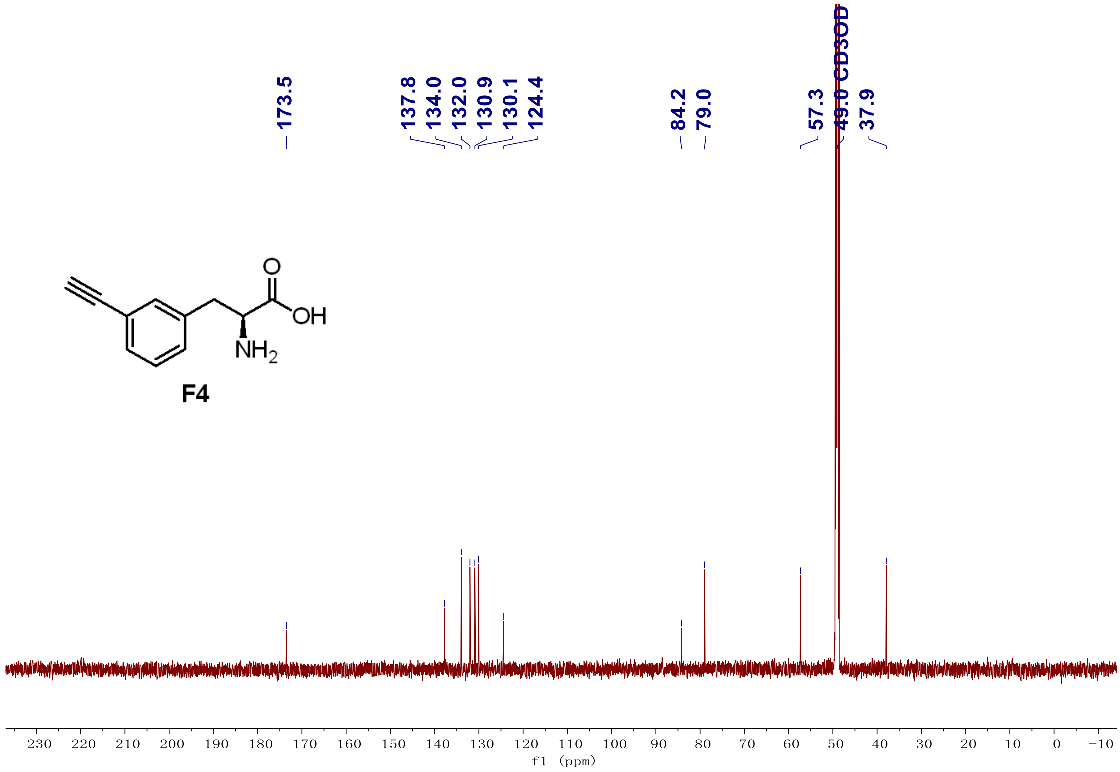

Supplementary Figure 35 ^{13}C NMR spectrum of **F4** in methanol-d_4.

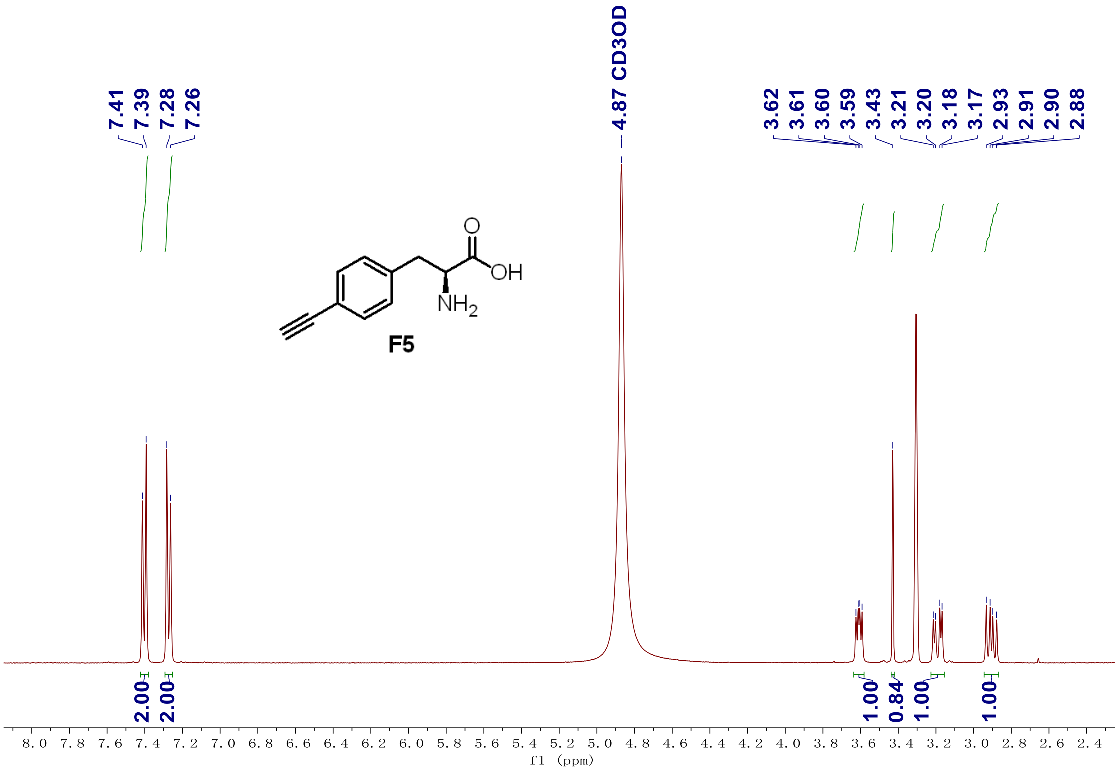

Supplementary Figure 36 ^{1}H NMR spectrum of **F5** in methanol-d_4.

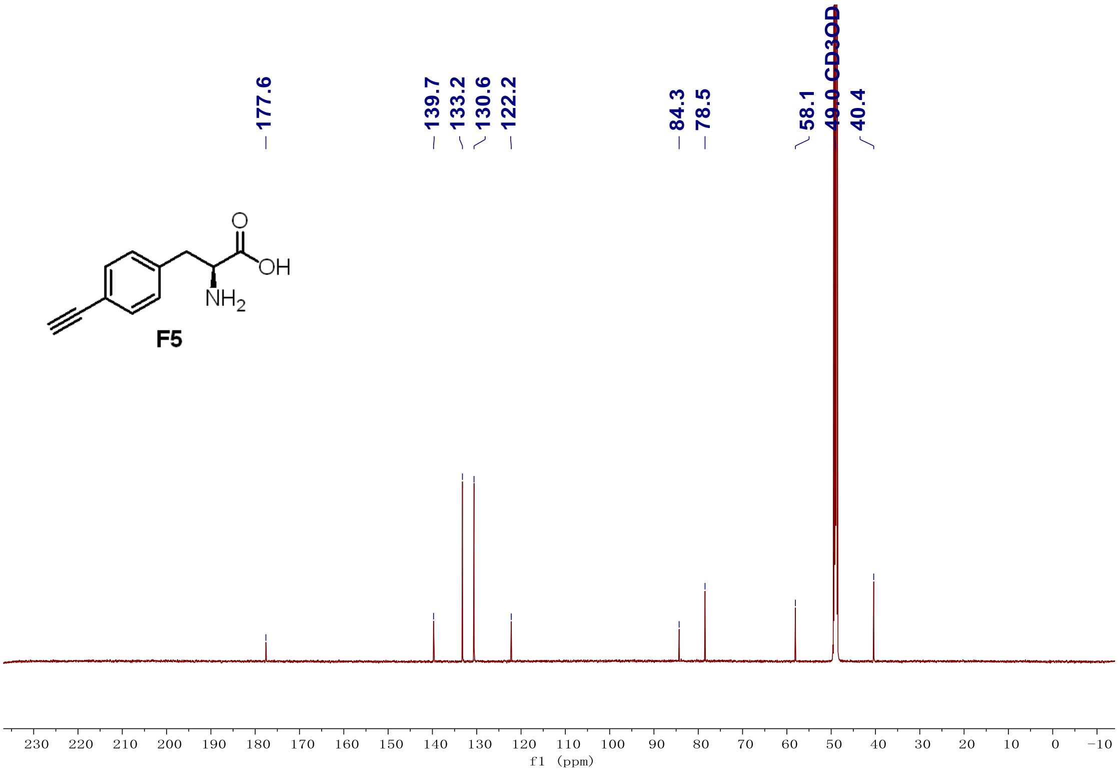

Supplementary Figure 37 ^{13}C NMR spectrum of **F5** in methanol-d_4.

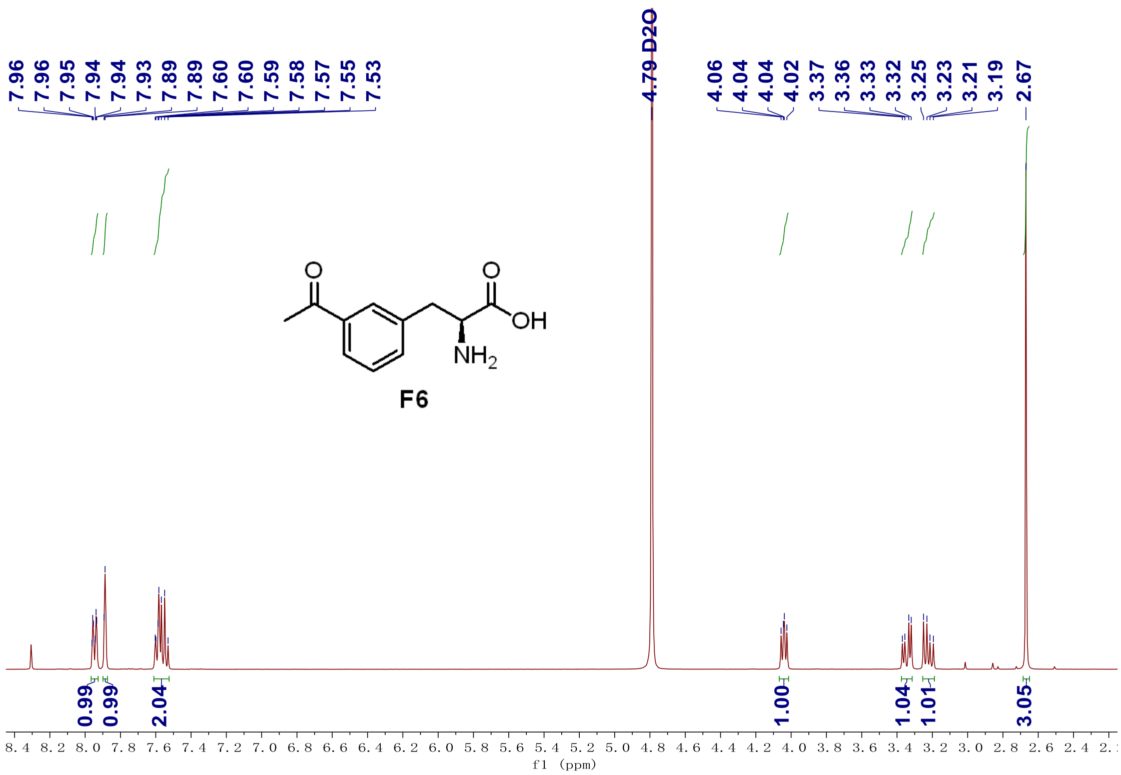

Supplementary Figure 38 ^{1}H NMR spectrum of **F6** in D_2O.

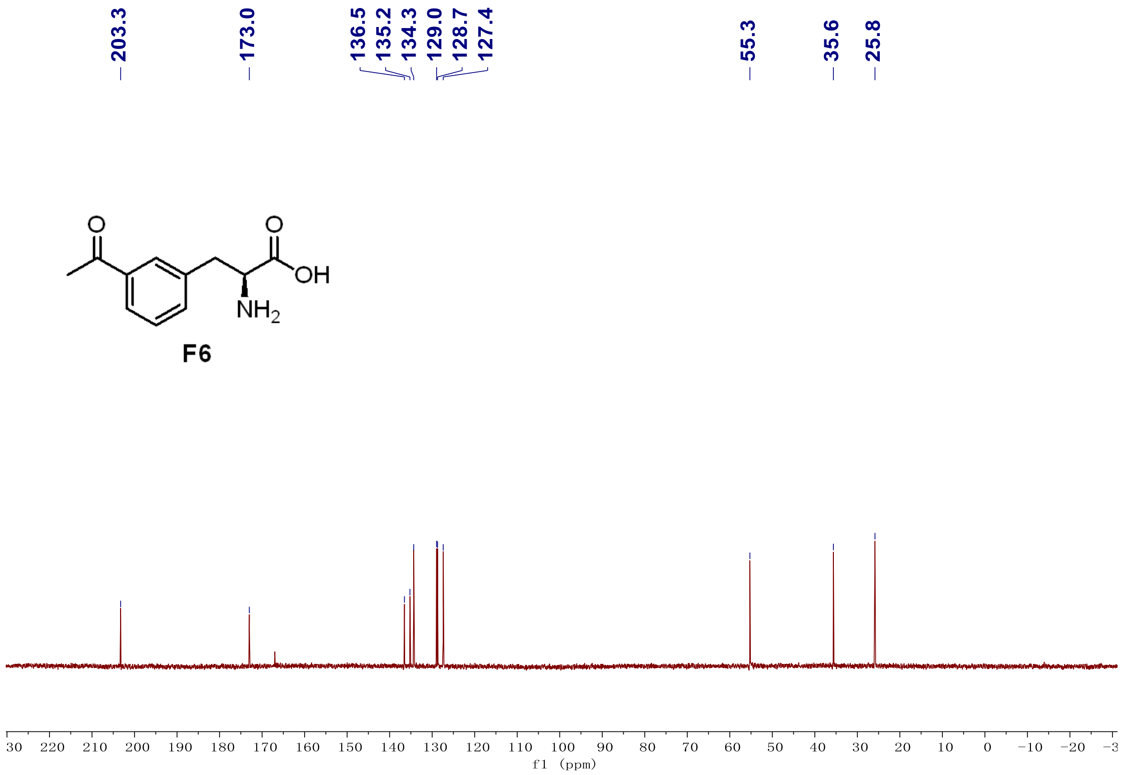

Supplementary Figure 39 ^{13}C NMR spectrum of **F6** in D_2O.

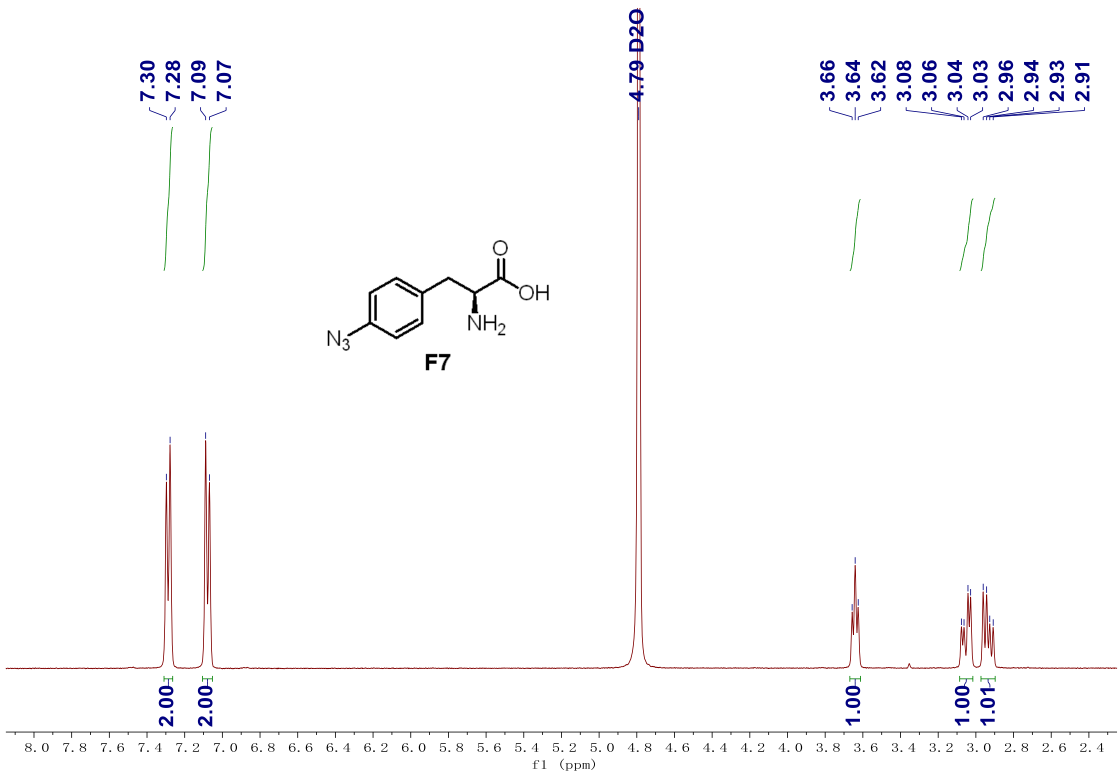

Supplementary Figure 40 ^{1}H NMR spectrum of **F7** in D_2O with NaOH.

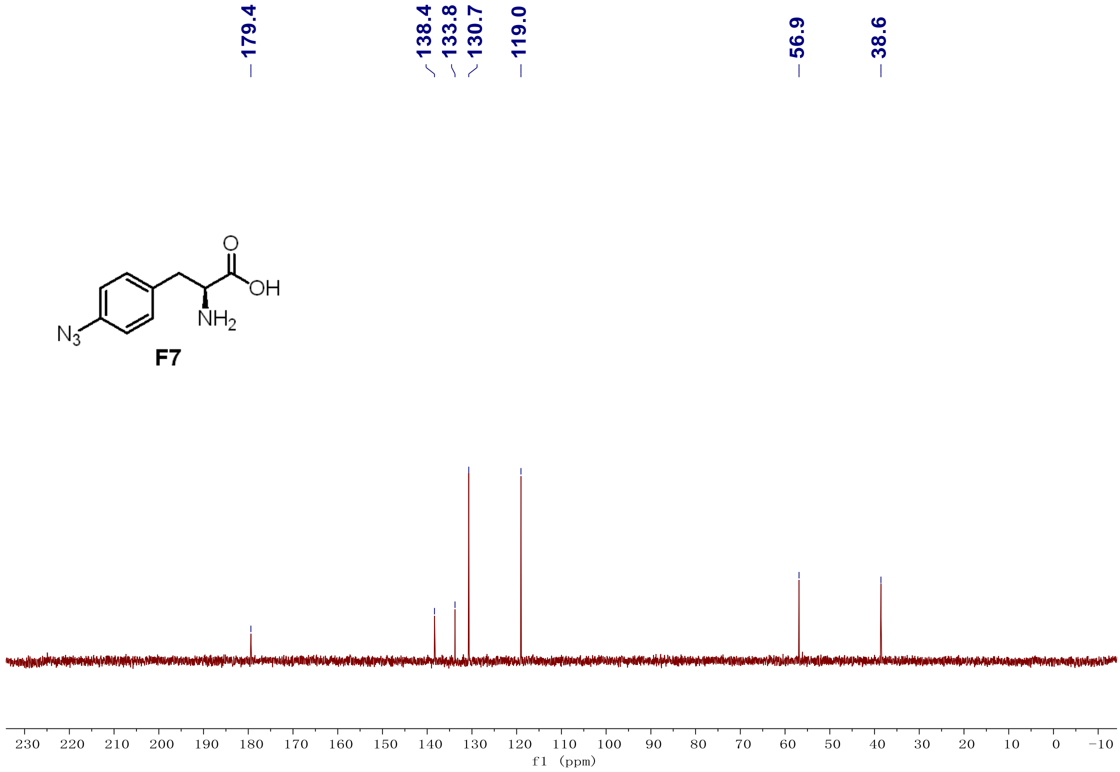

Supplementary Figure 41 ^{13}C NMR spectrum of **F7** in D_2O with NaOH.

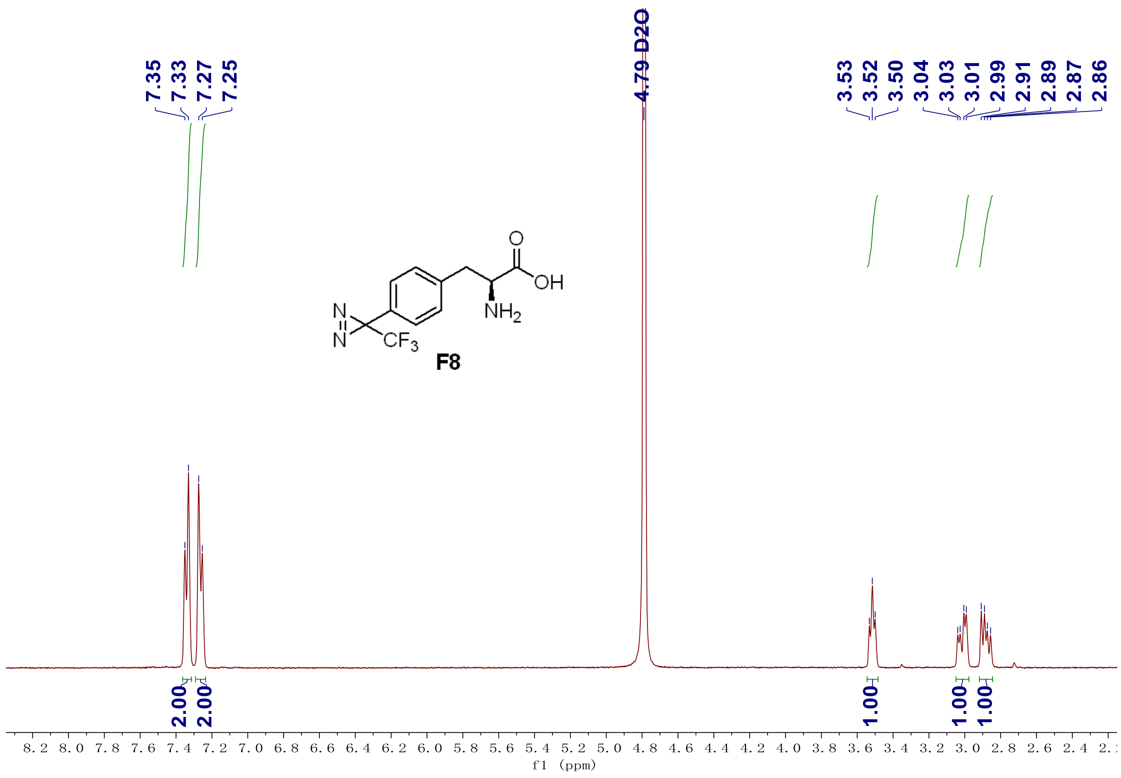

Supplementary Figure 42 ^{1}H NMR spectrum of **F8** in D_2O with NaOH.

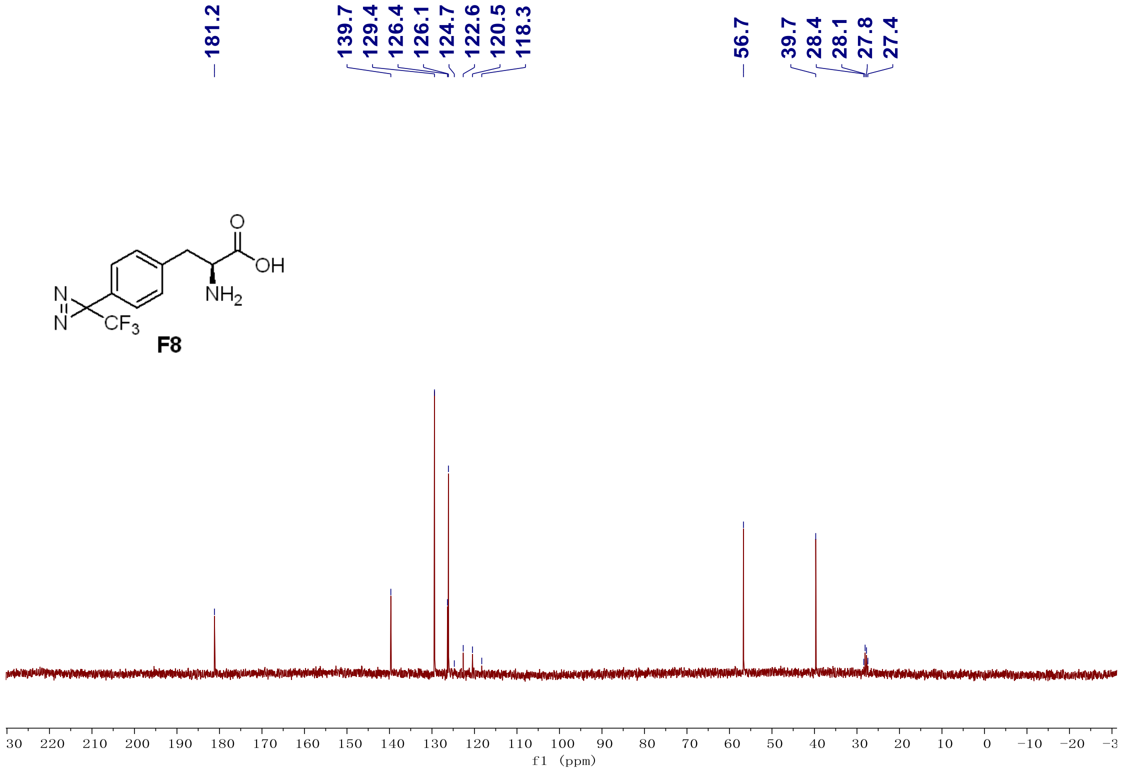

Supplementary Figure 43 ^{13}C NMR spectrum of **F8** in D_2O with NaOH.

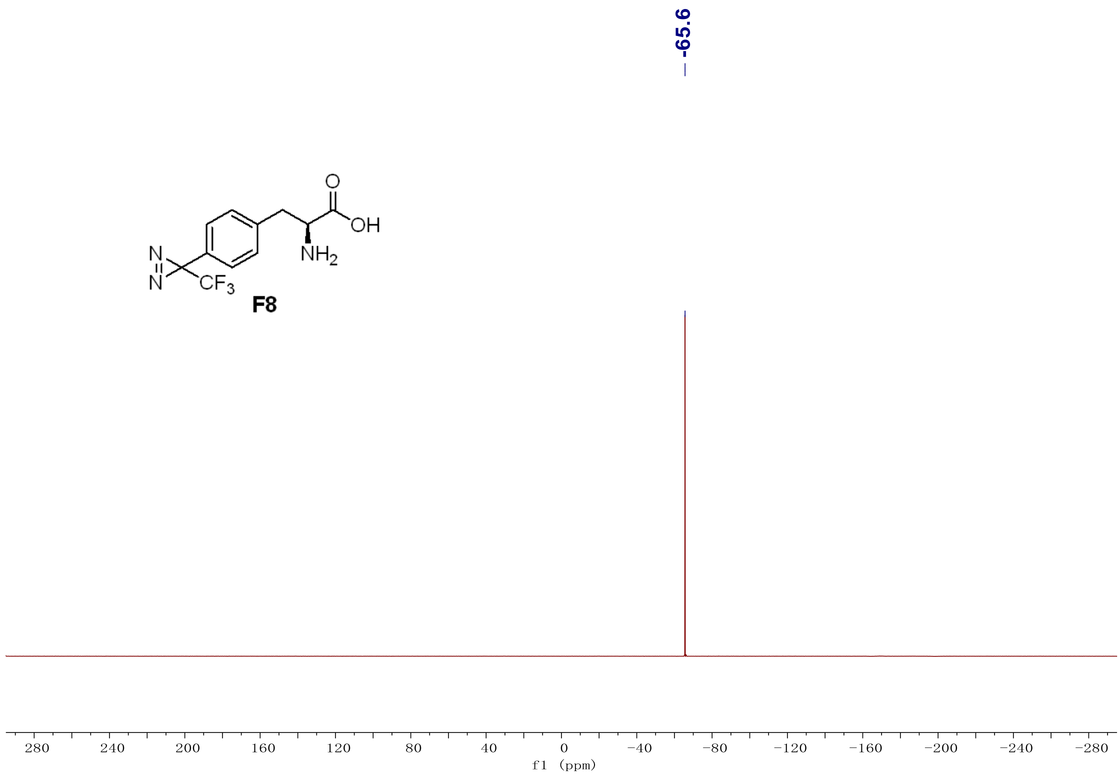

Supplementary Figure 44 ^{19}F NMR spectrum of **F8** in D_2O with NaOH.

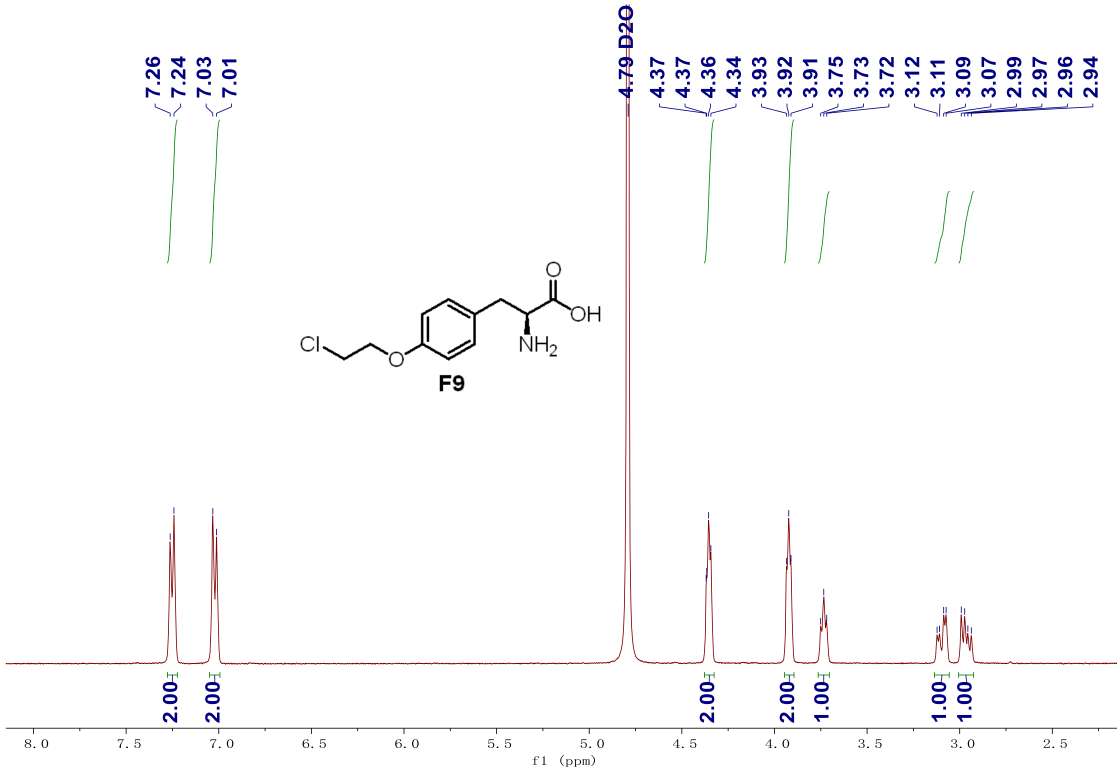

Supplementary Figure 45 ^{1}H NMR spectrum of **F9** in D_2O with NaOH.

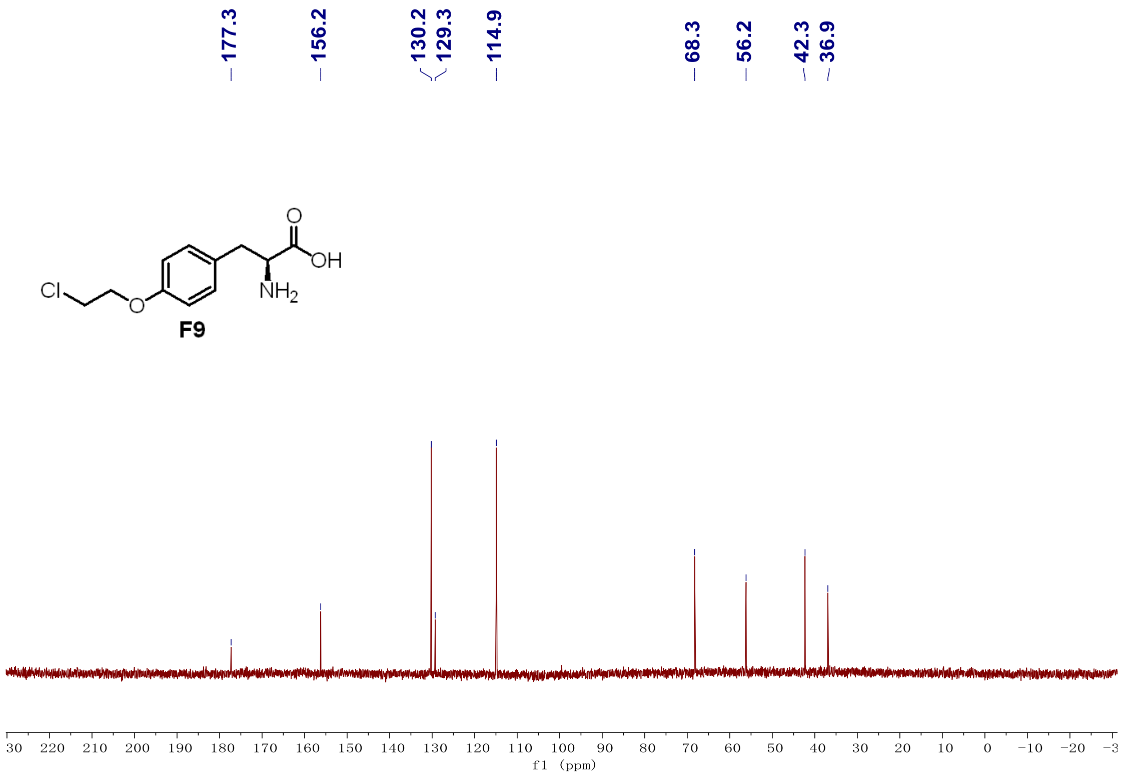

Supplementary Figure 46 ^{13}C NMR spectrum of **F9** in D_2O with NaOH.

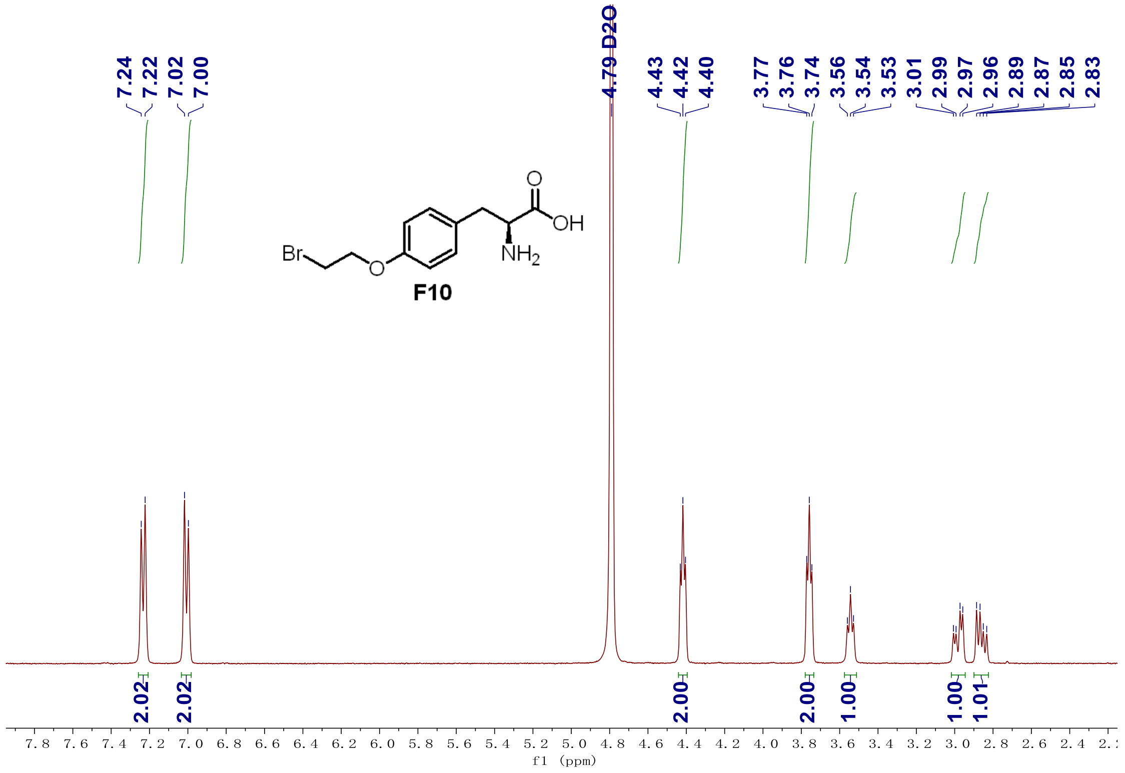

Supplementary Figure 47 ^{1}H NMR spectrum of **F10** in D_2O with NaOH.

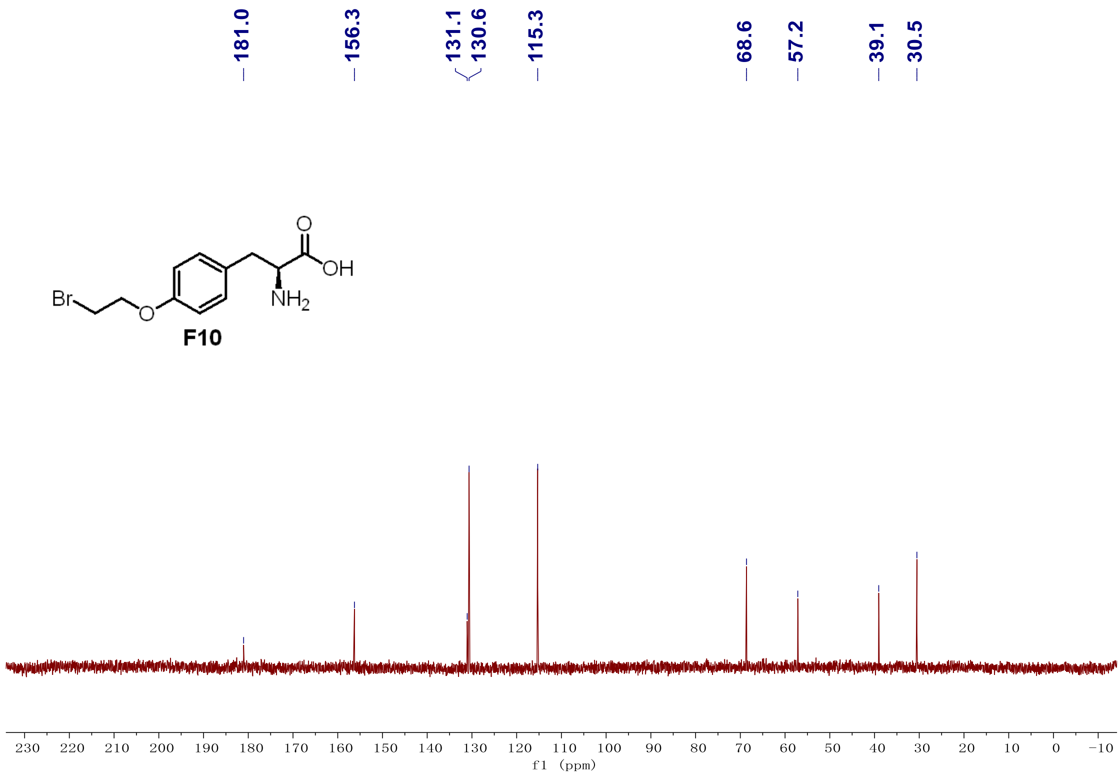

Supplementary Figure 48 ^{13}C NMR spectrum of **F10** in D_2O with NaOH.

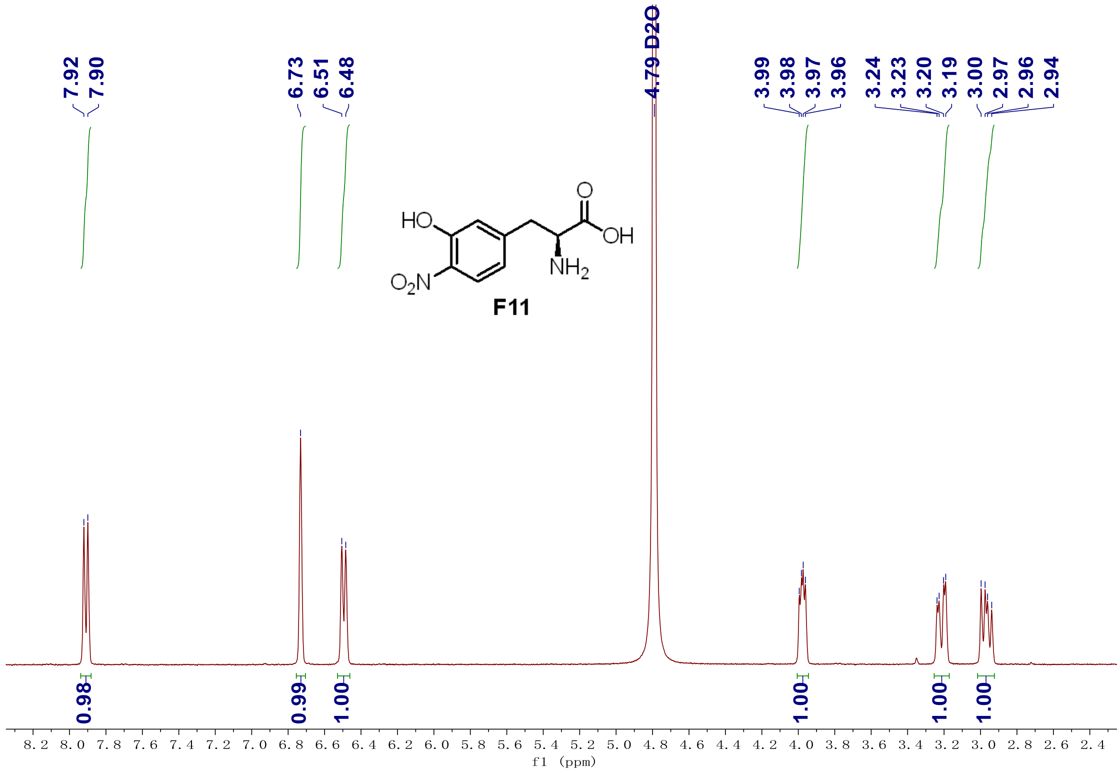

Supplementary Figure 49 ^{1}H NMR spectrum of **F11** in D_2O with NaOH.

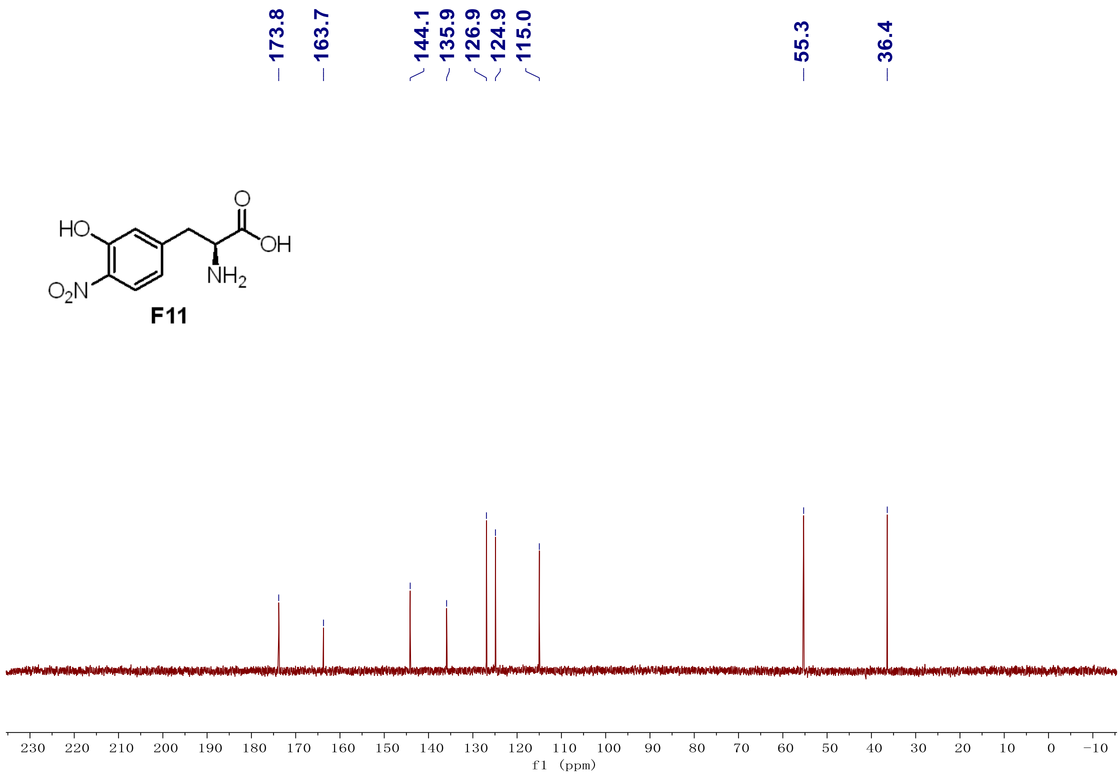

Supplementary Figure 50 ^{13}C NMR spectrum of **F11** in D_2O with NaOH.

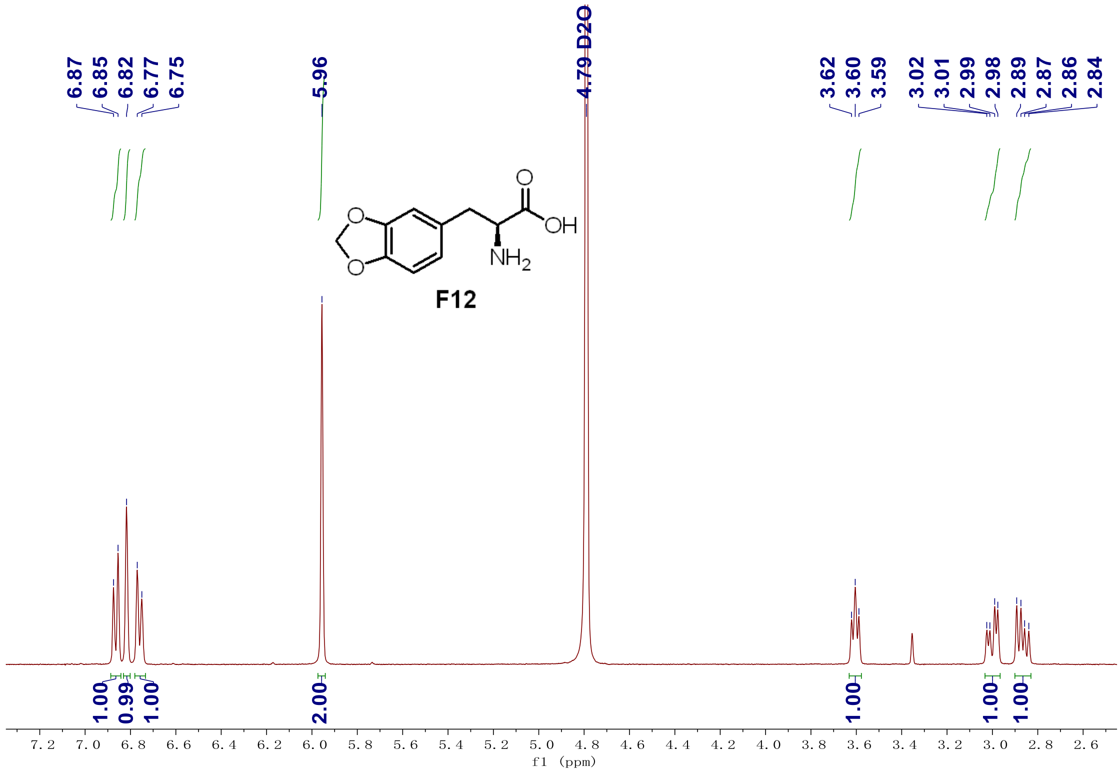

Supplementary Figure 51 ^{1}H NMR spectrum of **F12** in D_2O with NaOH.

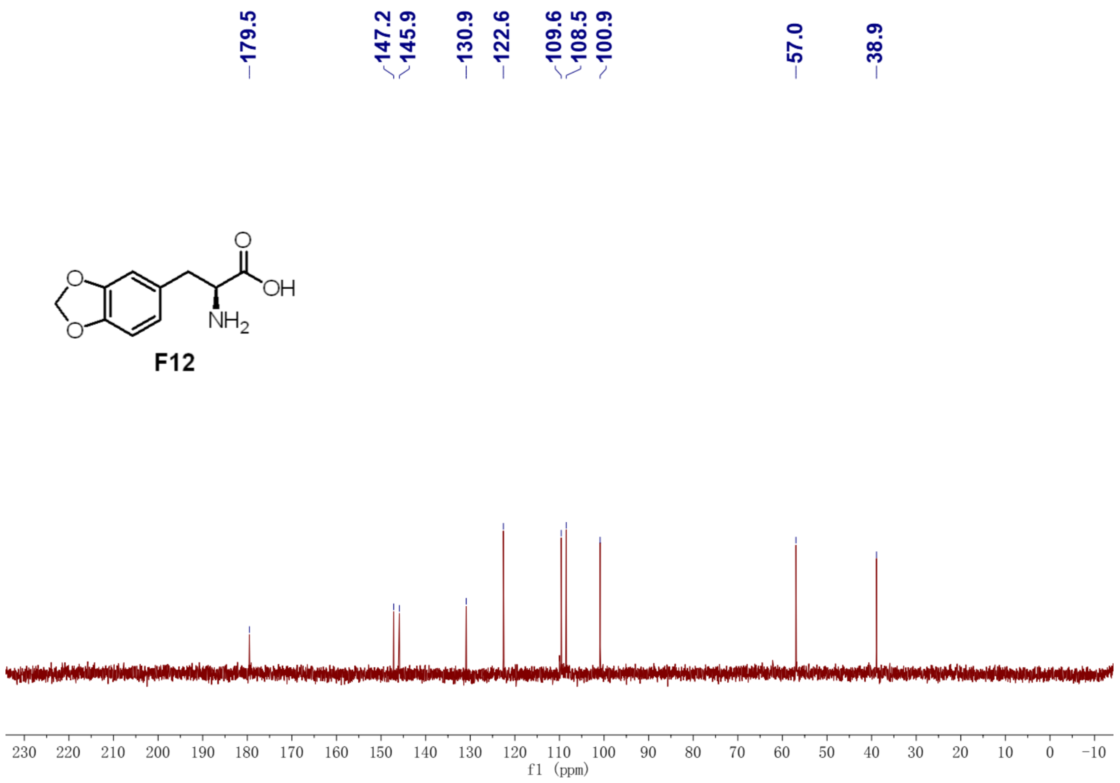

Supplementary Figure 52 ^{13}C NMR spectrum of **F12** in D_2O with NaOH.

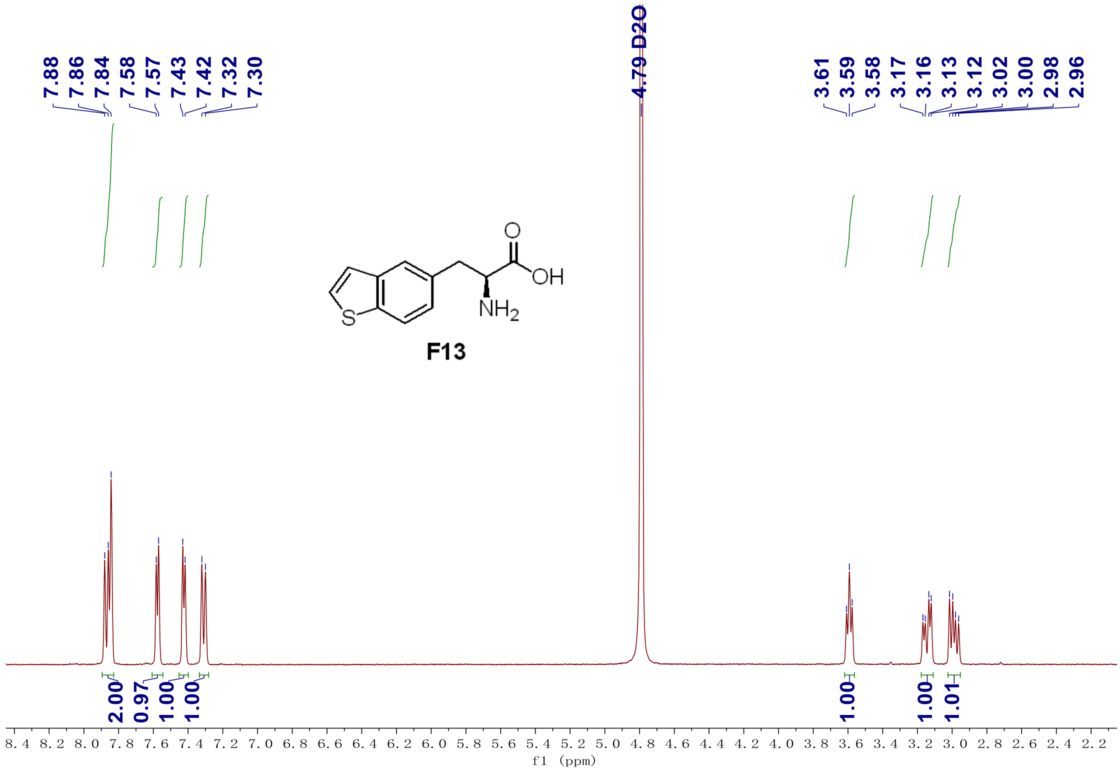

Supplementary Figure 53 ^{1}H NMR spectrum of **F13** in D_2O with NaOH.

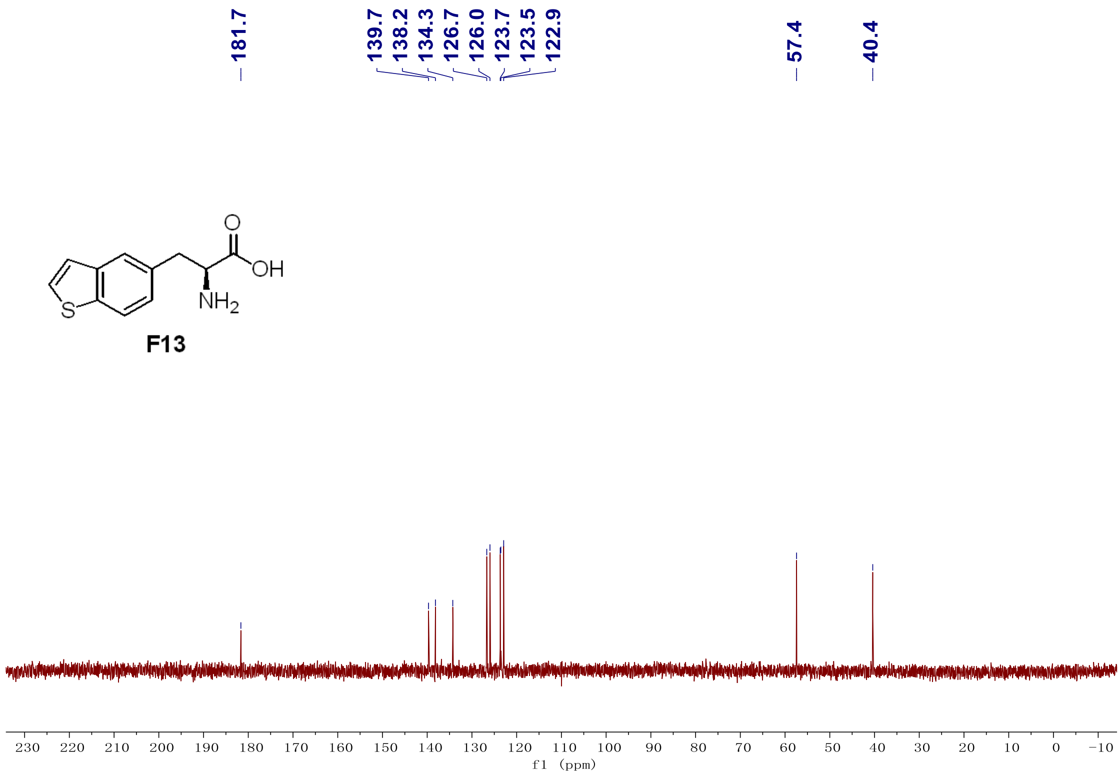

Supplementary Figure 54 ^{13}C NMR spectrum of **F13** in D_2O with NaOH.

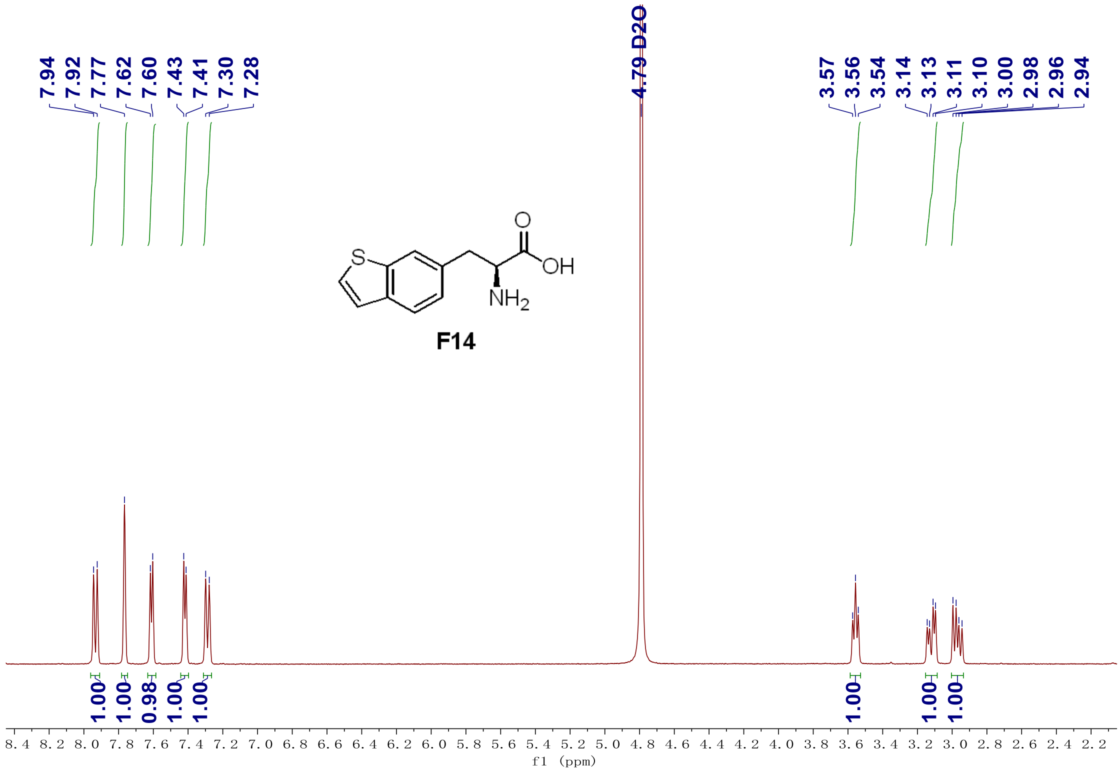

Supplementary Figure 55 ^{1}H NMR spectrum of **F14** in D_2O with NaOH.

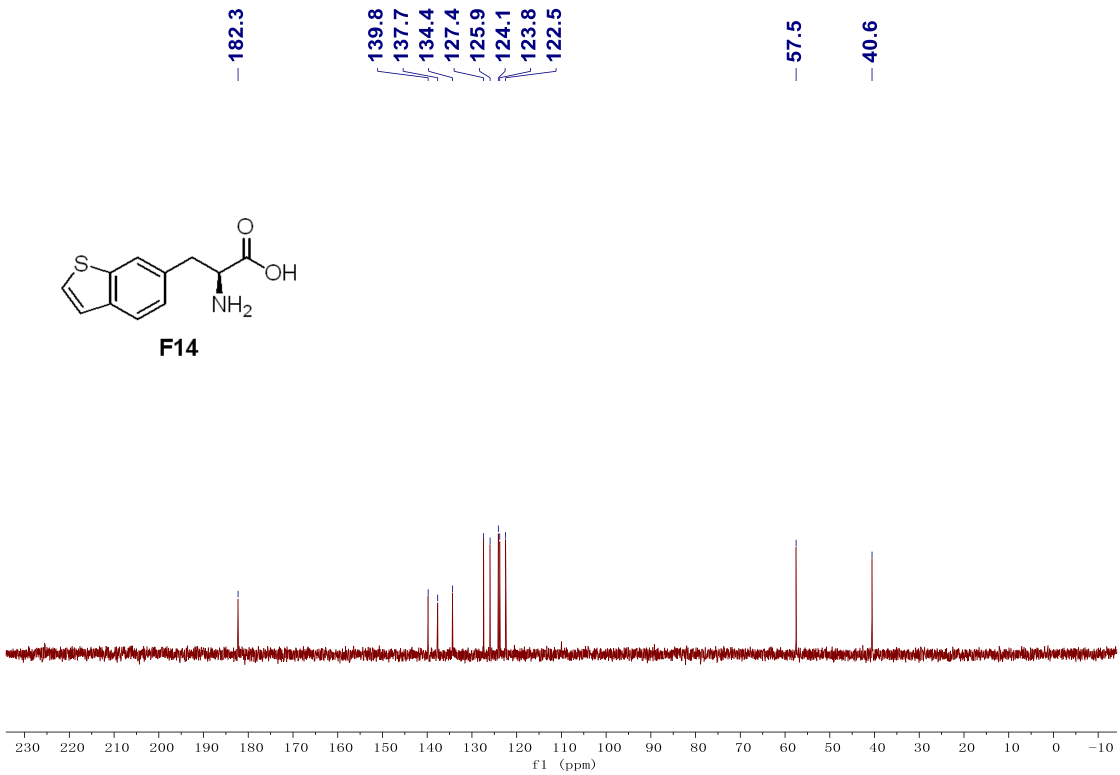

Supplementary Figure 56 ^{13}C NMR spectrum of **F14** in D_2O with NaOH.

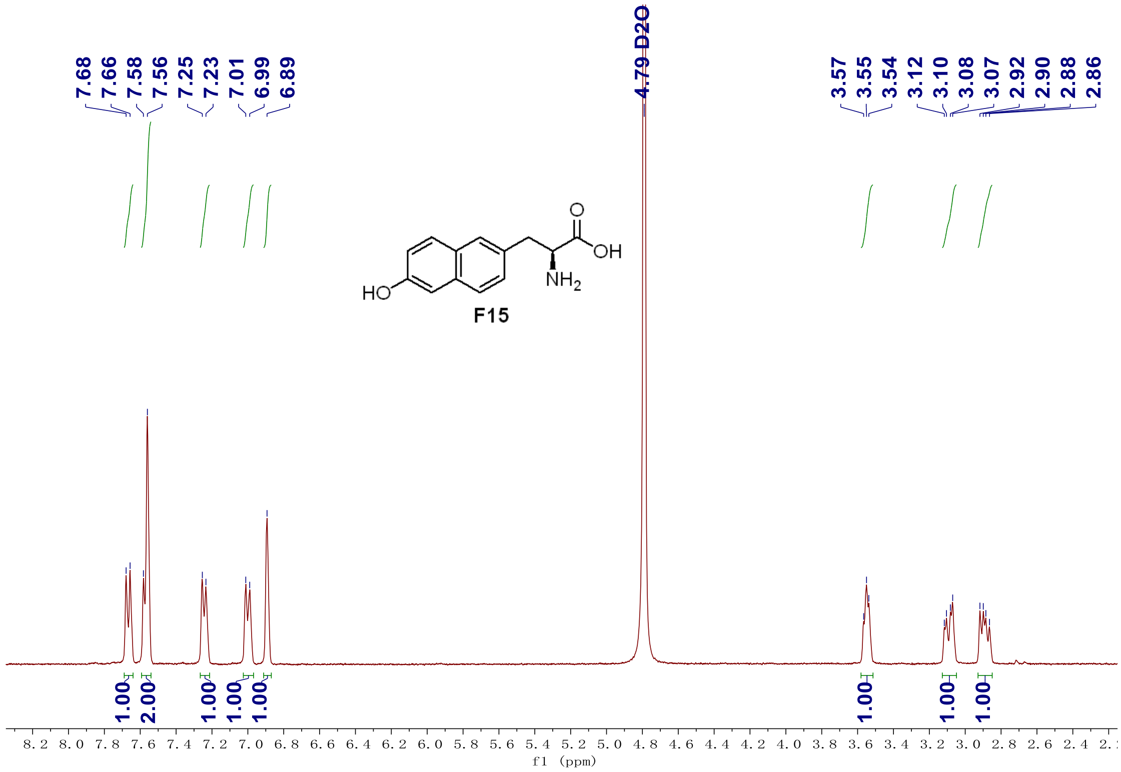

Supplementary Figure 57 ^{1}H NMR spectrum of **F15** in D_2O with NaOH.

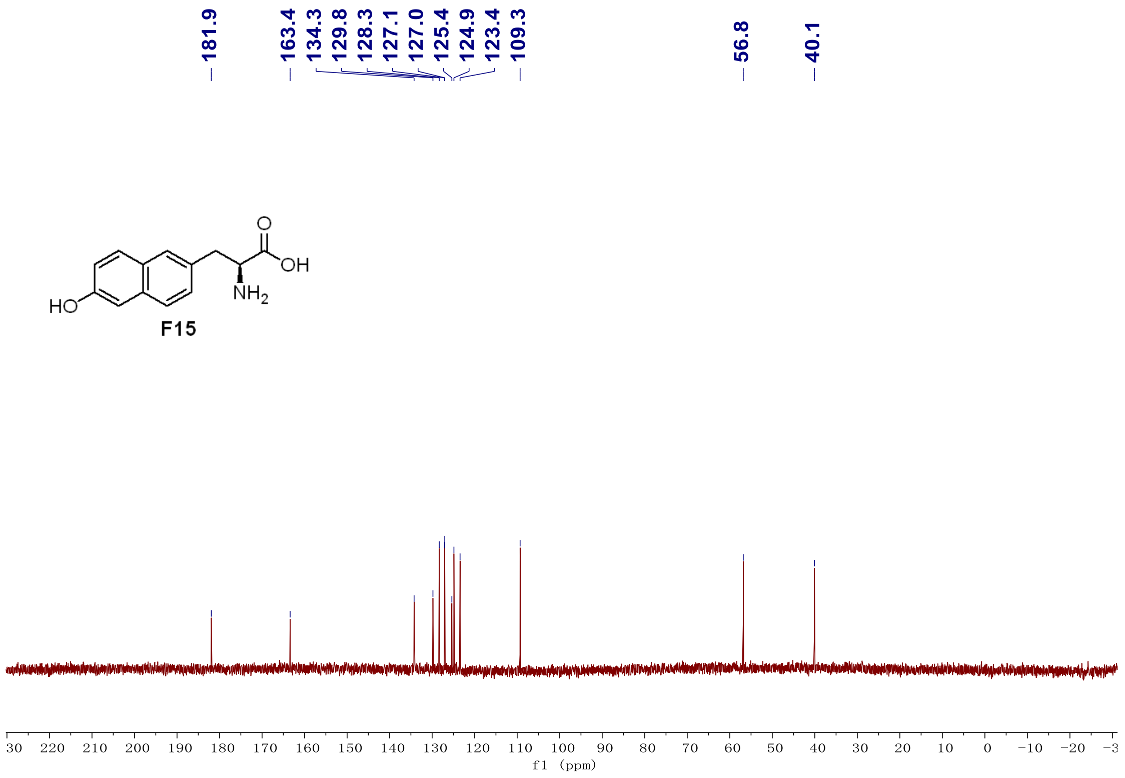

Supplementary Figure 58 ^{13}C NMR spectrum of **F15** in D_2O with NaOH.

High resolution mass spectra

Qualitative Analysis Report

**Data Filename**	ESIH202401254.d	**Sample Name**	G5-F1
**Sample ID**		**Position**	P1-B5
**Instrument Name**	Agilent 6520 Q-TOF	**Acq Method**	20160322_MS_ESIH_POS_1min.m
**Acquired Time**	3/11/2024 2:16:23 PM	**DA Method**	ESI-HR-20231114.m
**Comment**	ESIH by fangsu		

User Spectra

Fragmentor Voltage	Collision Energy	Ionization Mode
170	0	ESI

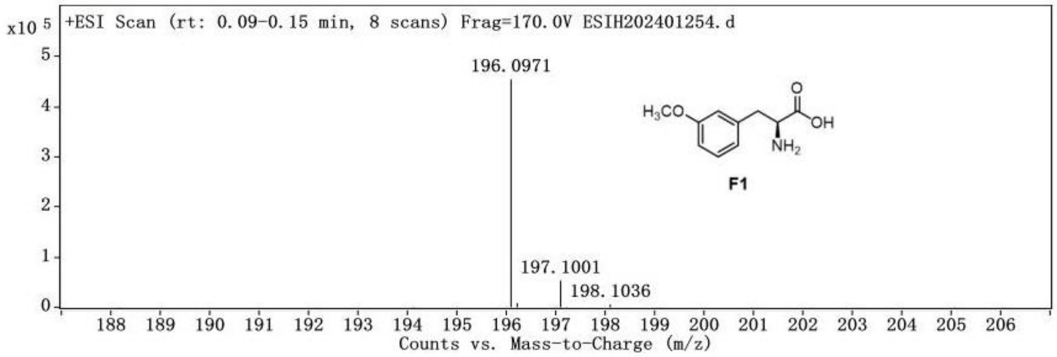

Formula Calculator Results

m/z	Calc m/z	Diff (mDa)	Diff (ppm)	Ion Formula	Ion
196.0971	196.0968	-0.29	-1.46	C10 H14 N O3	(M+H)+

--- End Of Report ---

Supplementary Figure 59 ESI-HR MS for **F1**.

Qualitative Analysis Report

**Data Filename**	ESIH202400501.d	**Sample Name**	G5-ZWS-3OCF3
**Sample ID**		**Position**	P1-C9
**Instrument Name**	Agilent 6520 Q-TOF	**Acq Method**	20160322_MS_ESIH_POS_1min.m
**Acquired Time**	1/29/2024 4:18:24 PM	**DA Method**	ESI-HR-20231114.m
**Comment**	ESIH by fangsu		

User Spectra

Fragmentor Voltage	Collision Energy	Ionization Mode
170	0	ESI

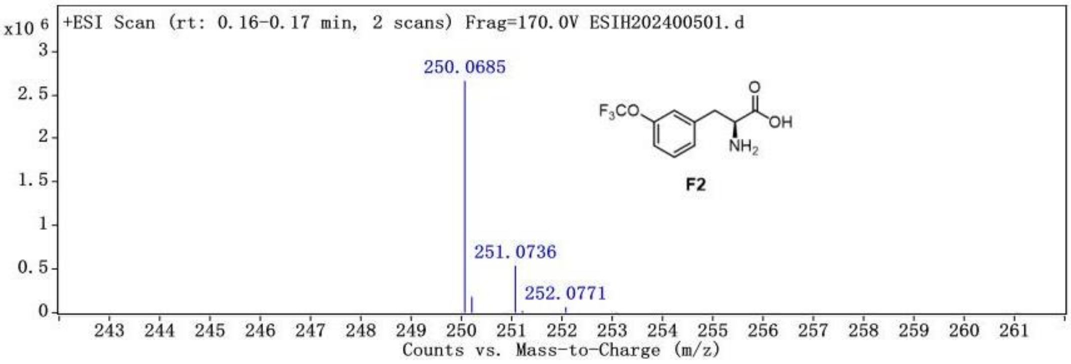

Formula Calculator Results

m/z	Calc m/z	Diff (mDa)	Diff (ppm)	Ion Formula	Ion
250.0685	250.0686	0.07	0.28	C10 H11 F3 N O3	(M+H)+

--- End Of Report ---

Supplementary Figure 60 ESI-HR MS for **F2**.

Qualitative Analysis Report

**Data Filename**	ESIH202401255.d	**Sample Name**	G5-F3
**Sample ID**		**Position**	P1-B6
**Instrument Name**	Agilent 6520 Q-TOF	**Acq Method**	20160322_MS_ESIH_POS_1min.m
**Acquired Time**	3/11/2024 2:17:40 PM	**DA Method**	ESI-HR-20231114.m
**Comment**	ESIH by fangsu		

User Spectra

Fragmentor Voltage	Collision Energy	Ionization Mode
170	0	ESI

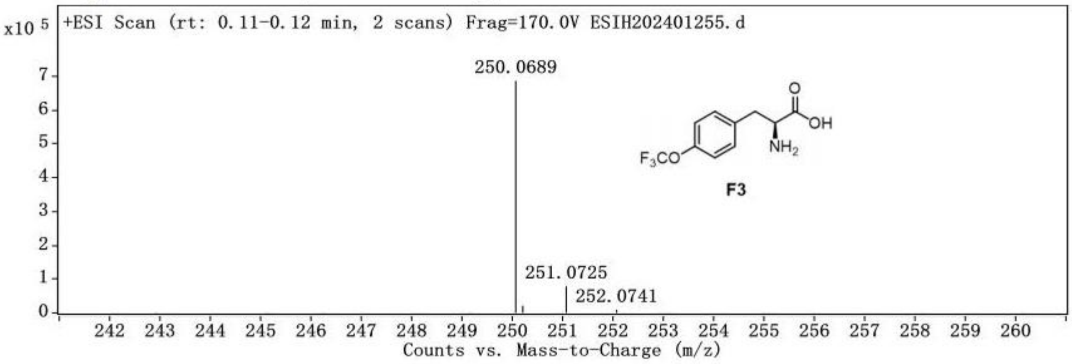

Formula Calculator Results

m/z	Calc m/z	Diff (mDa)	Diff (ppm)	Ion Formula	Ion
250.0689	250.0686	-0.36	-1.42	C10 H11 F3 N O3	(M+H)+

--- End Of Report ---

Supplementary Figure 61 ESI-HR MS for **F3**.

Qualitative Analysis Report

**Data Filename**	ESIH202400499.d	**Sample Name**	G5-ZWS-3Q
**Sample ID**		**Position**	P1-C7
**Instrument Name**	Agilent 6520 Q-TOF	**Acq Method**	20160322_MS_ESIH_POS_1min.m
**Acquired Time**	1/29/2024 4:15:51 PM	**DA Method**	ESI-HR-20231114.m
**Comment**	ESIH by fangsu		

User Spectra

Fragmentor Voltage	Collision Energy	Ionization Mode
170	0	ESI

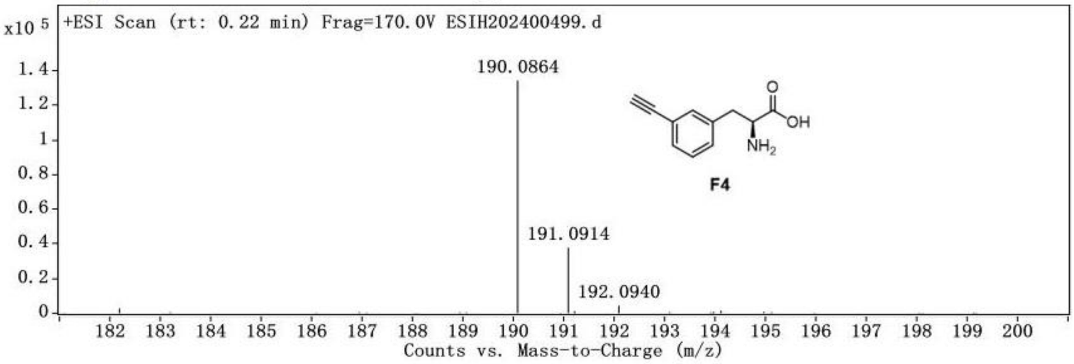

Formula Calculator Results

m/z	Calc m/z	Diff (mDa)	Diff (ppm)	Ion Formula	Ion
190.0864	190.0863	-0.17	-0.89	C11 H12 N O2	(M+H)+

--- End Of Report ---

Supplementary Figure 62 ESI-HR MS for **F4**.

Qualitative Analysis Report

**Data Filename**	ESIH202401256.d	**Sample Name**	G5-F5
**Sample ID**		**Position**	P1-B7
**Instrument Name**	Agilent 6520 Q-TOF	**Acq Method**	20160322_MS_ESIH_POS_1min.m
**Acquired Time**	3/11/2024 2:18:56 PM	**DA Method**	ESI-HR-20231114.m
**Comment**	ESIH by fangsu		

User Spectra

Fragmentor Voltage	Collision Energy	Ionization Mode
170	0	ESI

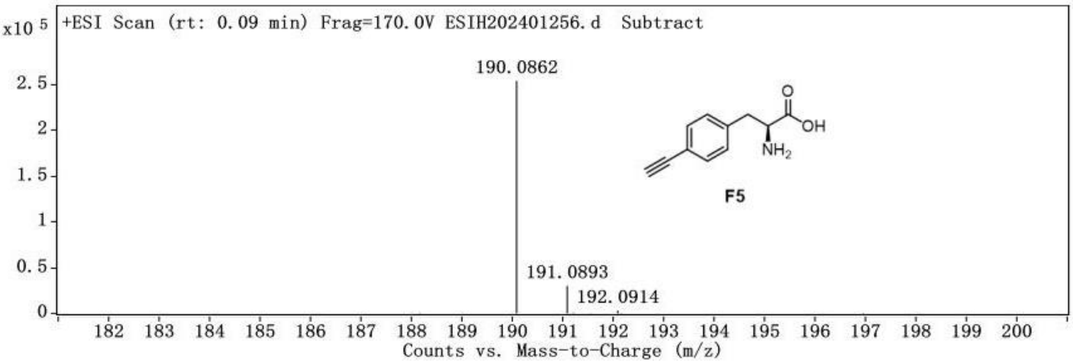

Formula Calculator Results

m/z	Calc m/z	Diff (mDa)	Diff (ppm)	Ion Formula	Ion
190.0862	190.0863	0.02	0.1	C11 H12 N O2	(M+H)+

--- End Of Report ---

Supplementary Figure 63 ESI-HR MS for **F5**.

Qualitative Analysis Report

**Data Filename**	ESIH202401257.d	**Sample Name**	G5-F6
**Sample ID**		**Position**	P1-B8
**Instrument Name**	Agilent 6520 Q-TOF	**Acq Method**	20160322_MS_ESIH_POS_1min.m
**Acquired Time**	3/11/2024 2:20:15 PM	**DA Method**	ESI-HR-20231114.m
**Comment**	ESIH by fangsu		

User Spectra

Fragmentor Voltage	Collision Energy	Ionization Mode
170	0	ESI

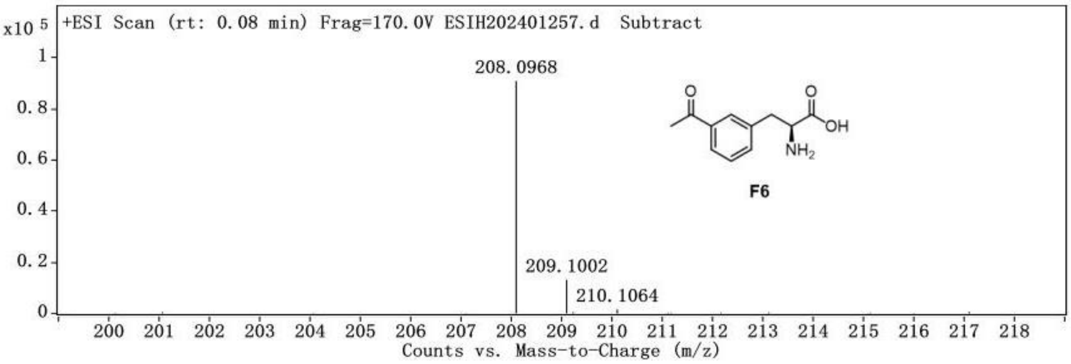

Formula Calculator Results

m/z	Calc m/z	Diff (mDa)	Diff (ppm)	Ion Formula	Ion
208.0968	208.0968	0.02	0.1	C11 H14 N O3	(M+H)+

--- End Of Report ---

Supplementary Figure 64 ESI-HR MS for **F6**.

Qualitative Analysis Report

**Data Filename**	ESIH202401258.d	**Sample Name**	G5-F7
**Sample ID**		**Position**	P1-B9
**Instrument Name**	Agilent 6520 Q-TOF	**Acq Method**	20160322_MS_ESIH_POS_1min.m
**Acquired Time**	3/11/2024 2:21:30 PM	**DA Method**	ESI-HR-20231114.m
**Comment**	ESIH by fangsu		

User Spectra

Fragmentor Voltage	Collision Energy	Ionization Mode
170	0	ESI

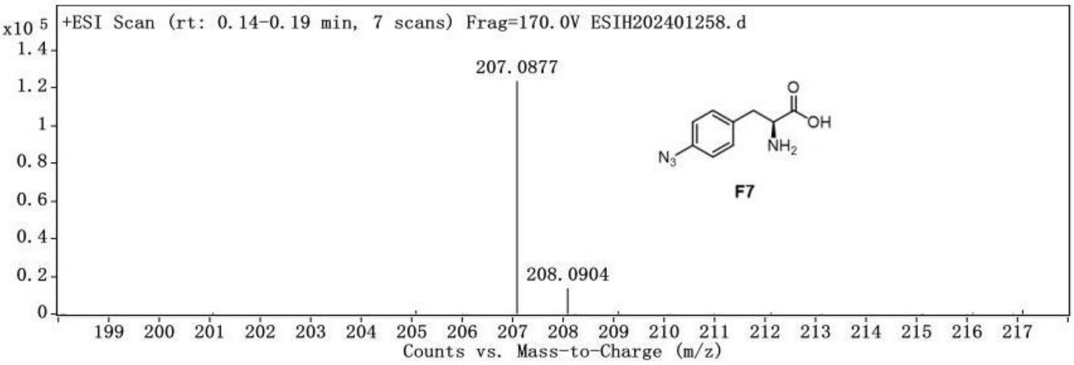

Formula Calculator Results

m/z	Calc m/z	Diff (mDa)	Diff (ppm)	Ion Formula	Ion
207.0877	207.0877	-0.08	-0.39	C9 H11 N4 O2	(M+H)+

--- End Of Report ---

Supplementary Figure 65 ESI-HR MS for **F7**.

Qualitative Analysis Report

**Data Filename**	ESIH202401259.d	**Sample Name**	G5-F8
**Sample ID**		**Position**	P1-C1
**Instrument Name**	Agilent 6520 Q-TOF	**Acq Method**	20160322_MS_ESIH_POS_1min.m
**Acquired Time**	3/11/2024 2:22:52 PM	**DA Method**	ESI-HR-20231114.m
**Comment**	ESIH by fangsu		

User Spectra

Fragmentor Voltage	Collision Energy	Ionization Mode
170	0	ESI

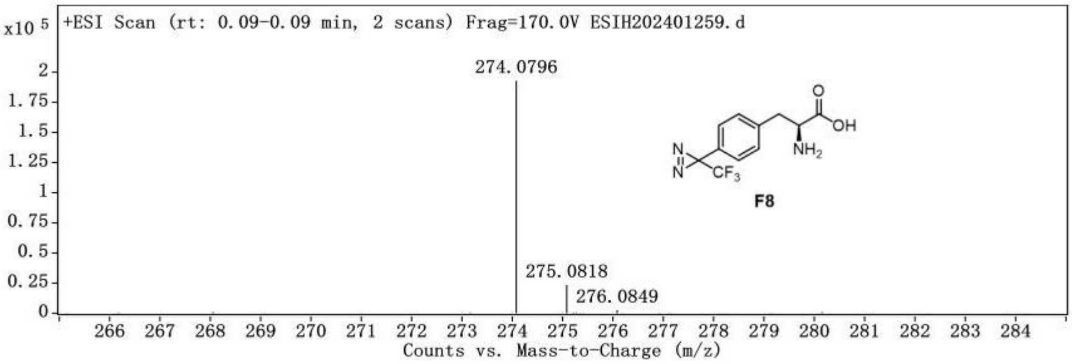

Formula Calculator Results

m/z	Calc m/z	Diff (mDa)	Diff (ppm)	Ion Formula	Ion
274.0796	274.0798	0.15	0.53	C11 H11 F3 N3 O2	(M+H)+

--- End Of Report ---

Supplementary Figure 66 ESI-HR MS for **F8**.

Qualitative Analysis Report

**Data Filename**	ESIH202401260.d	**Sample Name**	G5-F9
**Sample ID**		**Position**	P1-C2
**Instrument Name**	Agilent 6520 Q-TOF	**Acq Method**	20160322_MS_ESIH_POS_1min.m
**Acquired Time**	3/11/2024 2:24:07 PM	**DA Method**	ESI-HR-20231114.m
**Comment**	ESIH by fangsu		

User Spectra

Fragmentor Voltage	Collision Energy	Ionization Mode
170	0	ESI

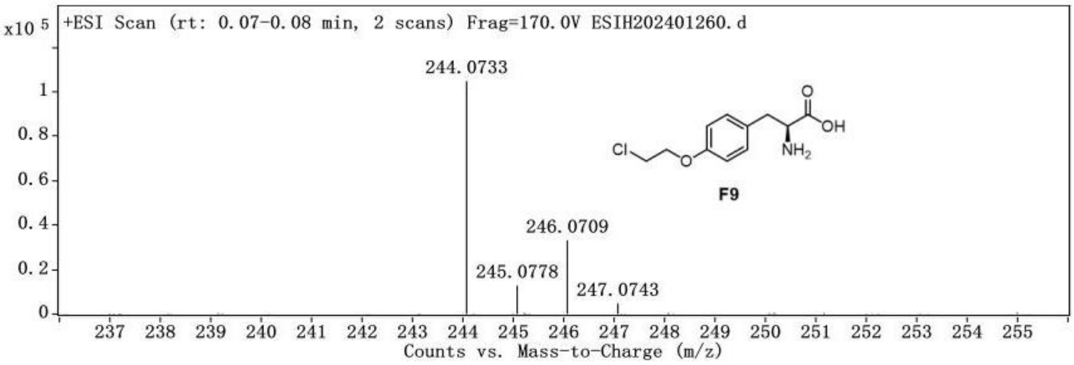

Formula Calculator Results

m/z	Calc m/z	Diff (mDa)	Diff (ppm)	Ion Formula	Ion
244.0733	244.0735	0.19	0.76	C11 H15 Cl N O3	(M+H)+

--- End Of Report ---

Supplementary Figure 67 ESI-HR MS for **F9**.

Qualitative Analysis Report

**Data Filename**	ESIH202401261.d	**Sample Name**	G5-F10
**Sample ID**		**Position**	P1-C3
**Instrument Name**	Agilent 6520 Q-TOF	**Acq Method**	20160322_MS_ESIH_POS_1min.m
**Acquired Time**	3/11/2024 2:25:23 PM	**DA Method**	ESI-HR-20231114.m
**Comment**	ESIH by fangsu		

User Spectra

Fragmentor Voltage	Collision Energy	Ionization Mode
170	0	ESI

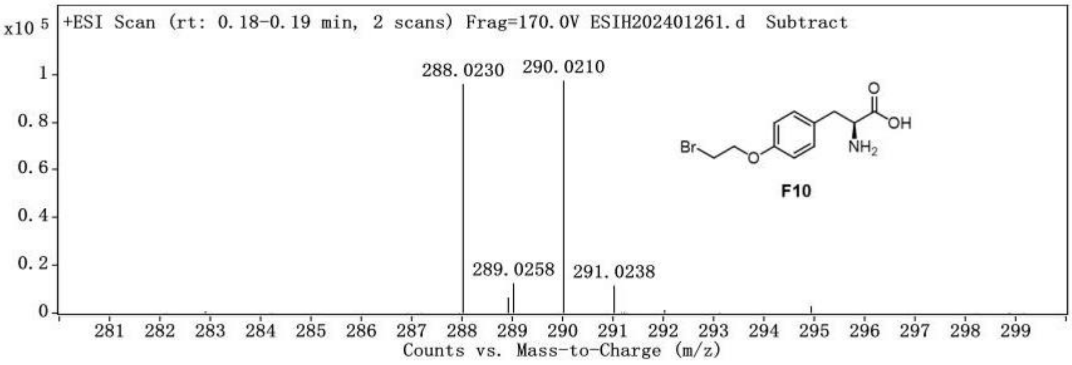

Formula Calculator Results

m/z	Calc m/z	Diff (mDa)	Diff (ppm)	Ion Formula	Ion
288.023	288.023	0.01	0.03	C11 H15 Br N O3	(M+H)+

--- End Of Report ---

Supplementary Figure 68 ESI-HR MS for **F10**.

Qualitative Analysis Report

**Data Filename**	ESIH202401262.d	**Sample Name**	G5-F11
**Sample ID**		**Position**	P1-C4
**Instrument Name**	Agilent 6520 Q-TOF	**Acq Method**	20160322_MS_ESIH_POS_1min.m
**Acquired Time**	3/11/2024 2:26:39 PM	**DA Method**	ESI-HR-20231114.m
**Comment**	ESIH by fangsu		

User Spectra

Fragmentor Voltage	Collision Energy	Ionization Mode
170	0	ESI

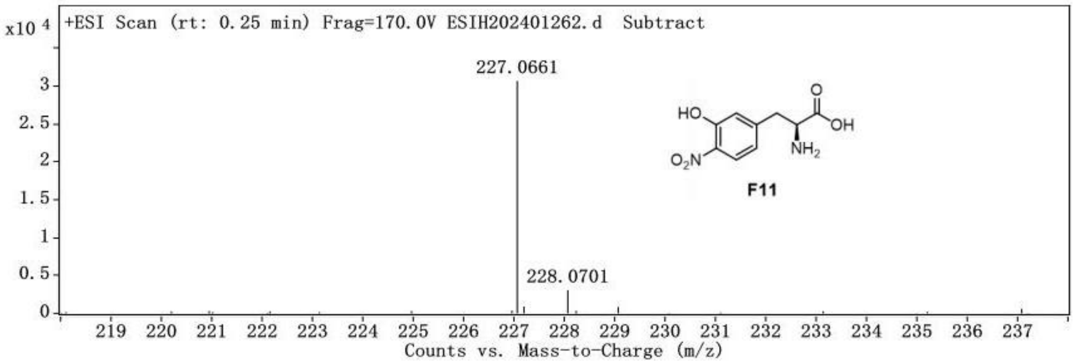

Formula Calculator Results

m/z	Calc m/z	Diff (mDa)	Diff (ppm)	Ion Formula	Ion
227.0661	227.0662	0.15	0.68	C9 H11 N2 O5	(M+H)+

--- End Of Report ---

Supplementary Figure 69 ESI-HR MS for **F11**.

Qualitative Analysis Report

**Data Filename**	ESIH202401263.d	**Sample Name**	G5-F12
**Sample ID**		**Position**	P1-C5
**Instrument Name**	Agilent 6520 Q-TOF	**Acq Method**	20160322_MS_ESIH_POS_1min.m
**Acquired Time**	3/11/2024 2:27:54 PM	**DA Method**	ESI-HR-20231114.m
**Comment**	ESIH by fangsu		

User Spectra

Fragmentor Voltage	Collision Energy	Ionization Mode
170	0	ESI

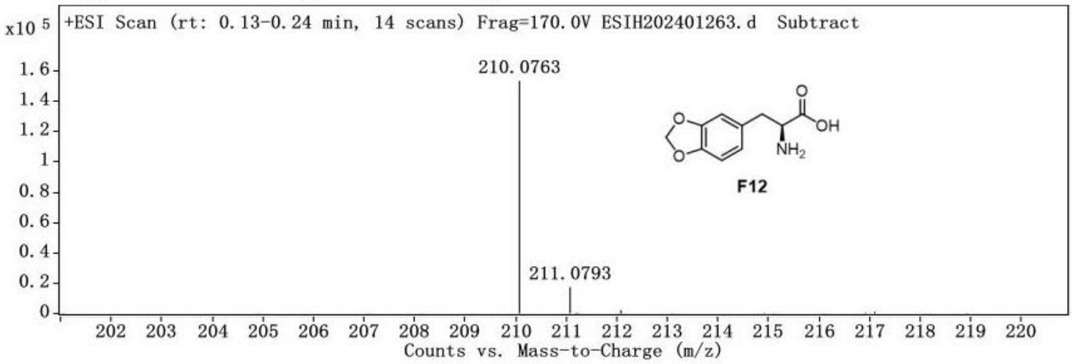

Formula Calculator Results

m/z	Calc m/z	Diff (mDa)	Diff (ppm)	Ion Formula	Ion
210.0763	210.0761	-0.21	-1	C10 H12 N O4	(M+H)+

--- End Of Report ---

Supplementary Figure 70 ESI-HR MS for **F12**.

Qualitative Analysis Report

**Data Filename**	ESIH202401264.d	**Sample Name**	G5-F13
**Sample ID**		**Position**	P1-C6
**Instrument Name**	Agilent 6520 Q-TOF	**Acq Method**	20160322_MS_ESIH_POS_1min.m
**Acquired Time**	3/11/2024 2:29:10 PM	**DA Method**	ESI-HR-20231114.m
**Comment**	ESIH by fangsu		

User Spectra

Fragmentor Voltage	Collision Energy	Ionization Mode
170	0	ESI

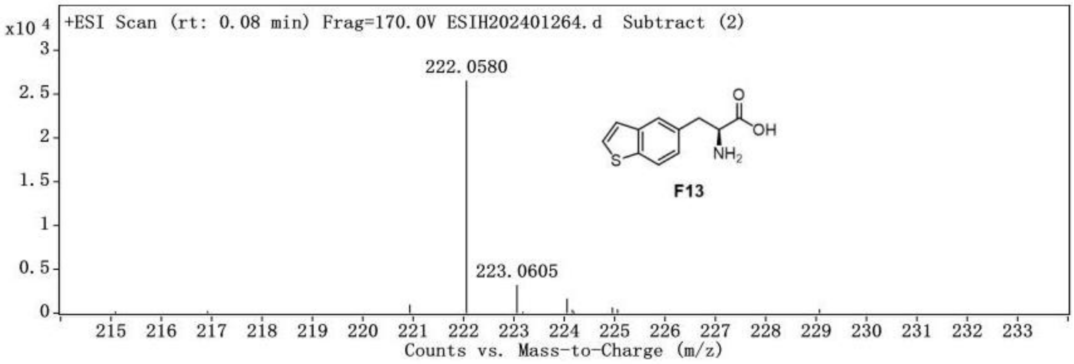

Formula Calculator Results

m/z	Calc m/z	Diff (mDa)	Diff (ppm)	Ion Formula	Ion
222.058	222.0583	0.32	1.42	C11 H12 N O2 S	(M+H)+

--- End Of Report ---

Supplementary Figure 71 ESI-HR MS for **F13**.

Qualitative Analysis Report

**Data Filename**	ESIH202401265.d	**Sample Name**	G5-F14
**Sample ID**		**Position**	P1-C7
**Instrument Name**	Agilent 6520 Q-TOF	**Acq Method**	20160322_MS_ESIH_POS_1min.m
**Acquired Time**	3/11/2024 2:30:27 PM	**DA Method**	ESI-HR-20231114.m
**Comment**	ESIH by fangsu		

User Spectra

Fragmentor Voltage	Collision Energy	Ionization Mode
170	0	ESI

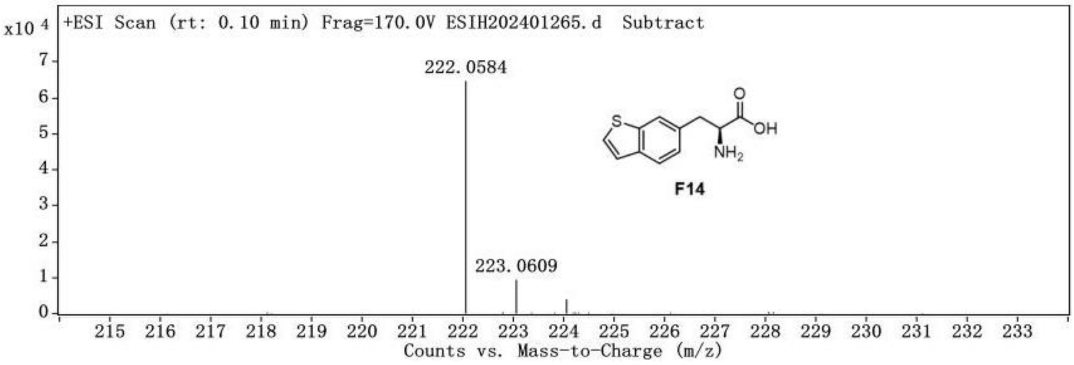

Formula Calculator Results

m/z	Calc m/z	Diff (mDa)	Diff (ppm)	Ion Formula	Ion
222.0584	222.0583	-0.06	-0.26	C11 H12 N O2 S	(M+H)+

--- End Of Report ---

Supplementary Figure 72 ESI-HR MS for **F14**.

Qualitative Analysis Report

**Data Filename**	ESIH202401266.d	**Sample Name**	G5-F15
**Sample ID**		**Position**	P1-C8
**Instrument Name**	Agilent 6520 Q-TOF	**Acq Method**	20160322_MS_ESIH_POS_1min.m
**Acquired Time**	3/11/2024 2:31:44 PM	**DA Method**	ESI-HR-20231114.m
**Comment**	ESIH by fangsu		

User Spectra

Fragmentor Voltage	Collision Energy	Ionization Mode
170	0	ESI

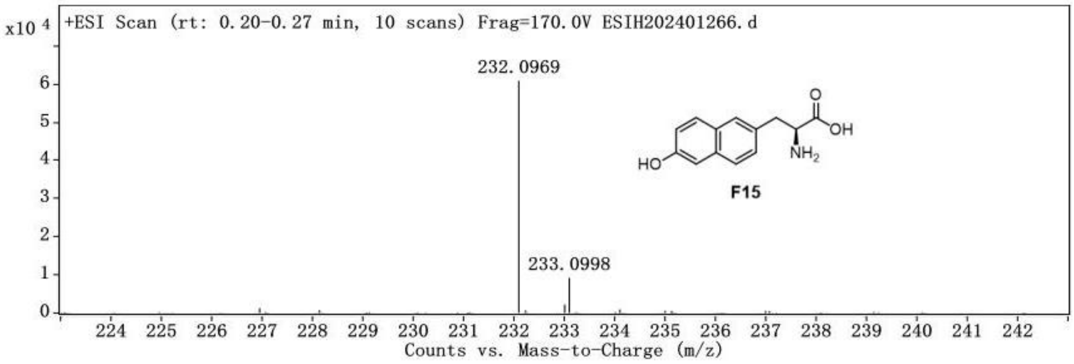

Formula Calculator Results

m/z	Calc m/z	Diff (mDa)	Diff (ppm)	Ion Formula	Ion
232.0969	232.0968	-0.05	-0.21	C13 H14 N O3	(M+H)+

--- End Of Report ---

Supplementary Figure 73 ESI-HR MS for **F15**.

Peptide macrocycles mass spectra

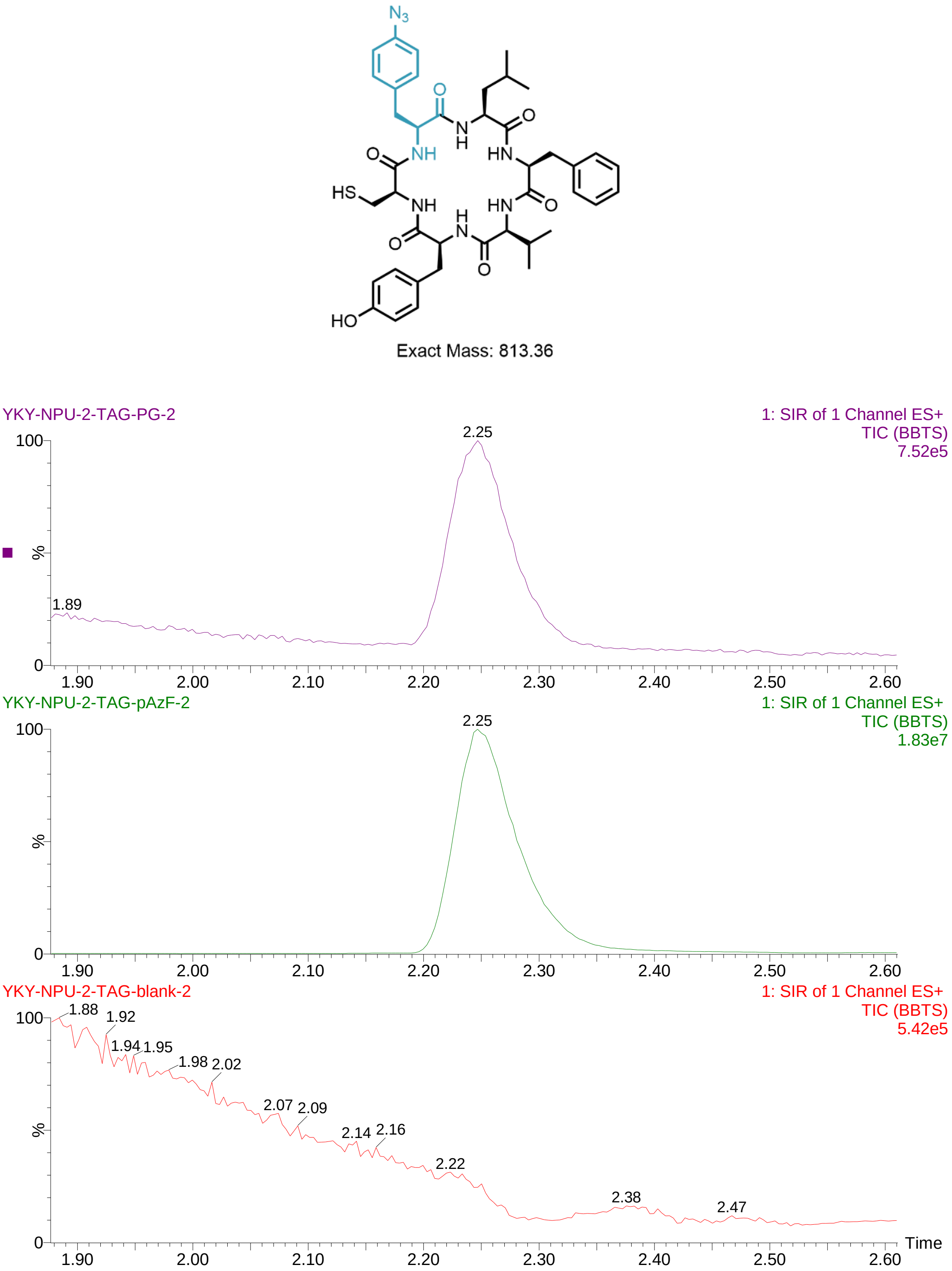

Supplementary Figure 74 Mass Spectrometry (MS) Analysis of cyclo-C[TAG]LFVY containing *p*-azidephenylalanine.

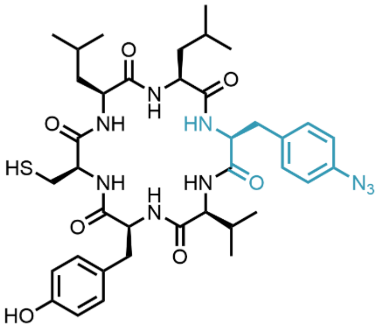

Exact Mass: 779.38

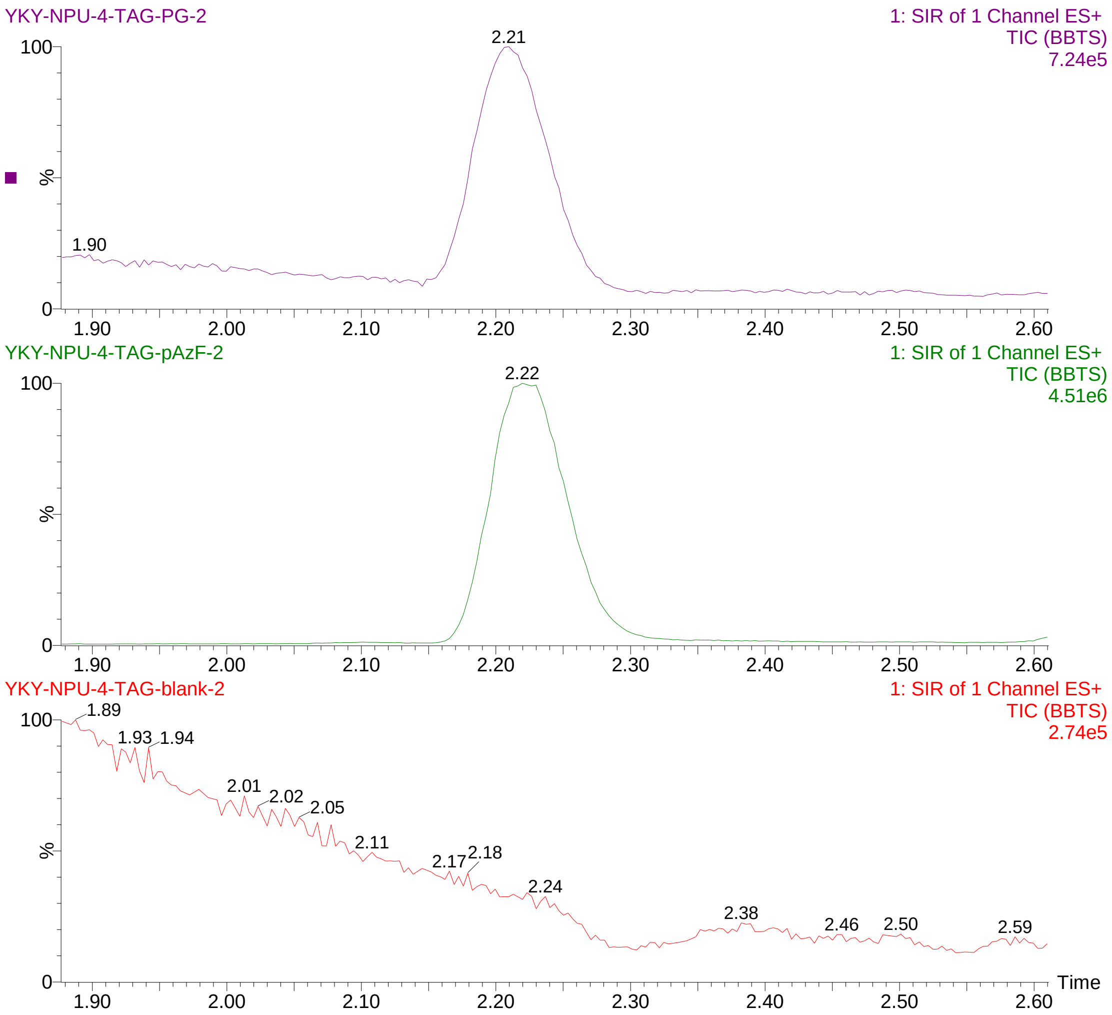

Supplementary Figure 75 Mass Spectrometry (MS) Analysis of cyclo-CLL[TAG]VY containing *p*-azidephenylalanine.

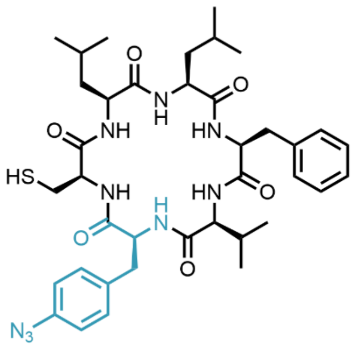

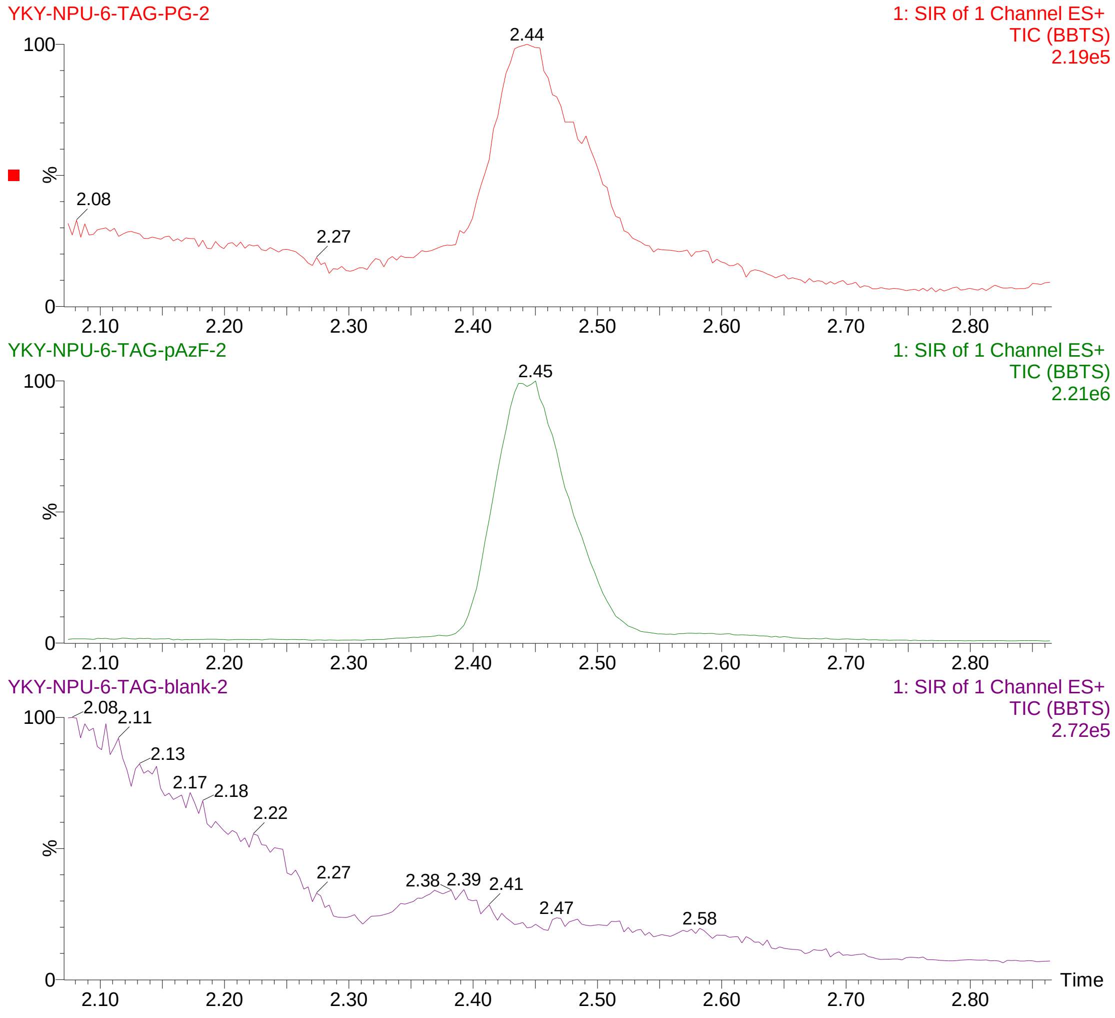

Supplementary Figure 76 Mass Spectrometry (MS) Analysis of cyclo-CLLFV[TAG] containing *p*-azidephenylalanine.

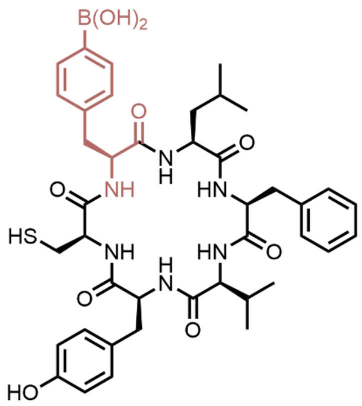

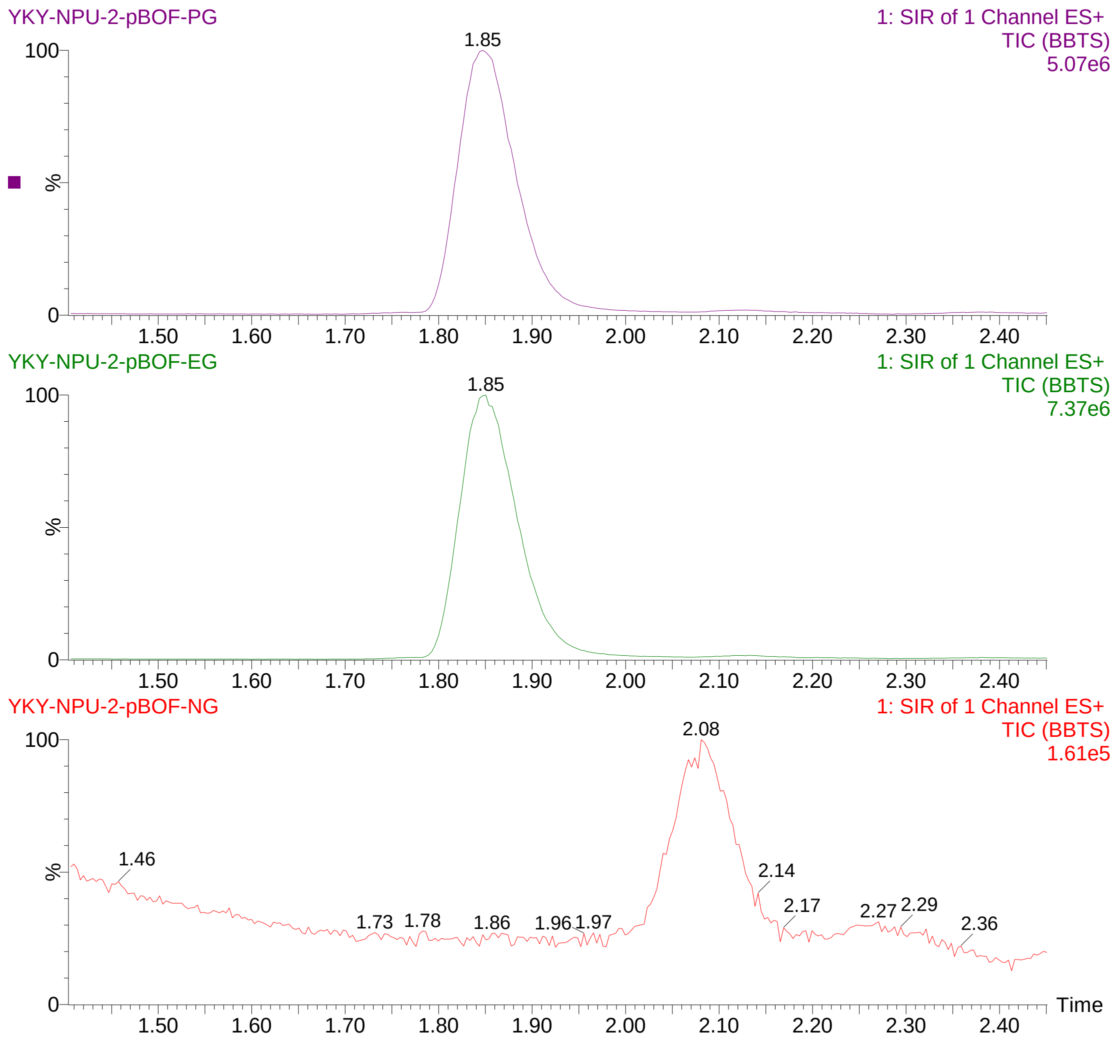

Supplementary Figure 77 Mass Spectrometry (MS) Analysis of cyclo-C[TAG]LFVY containing *p*-boronophenylalanine.

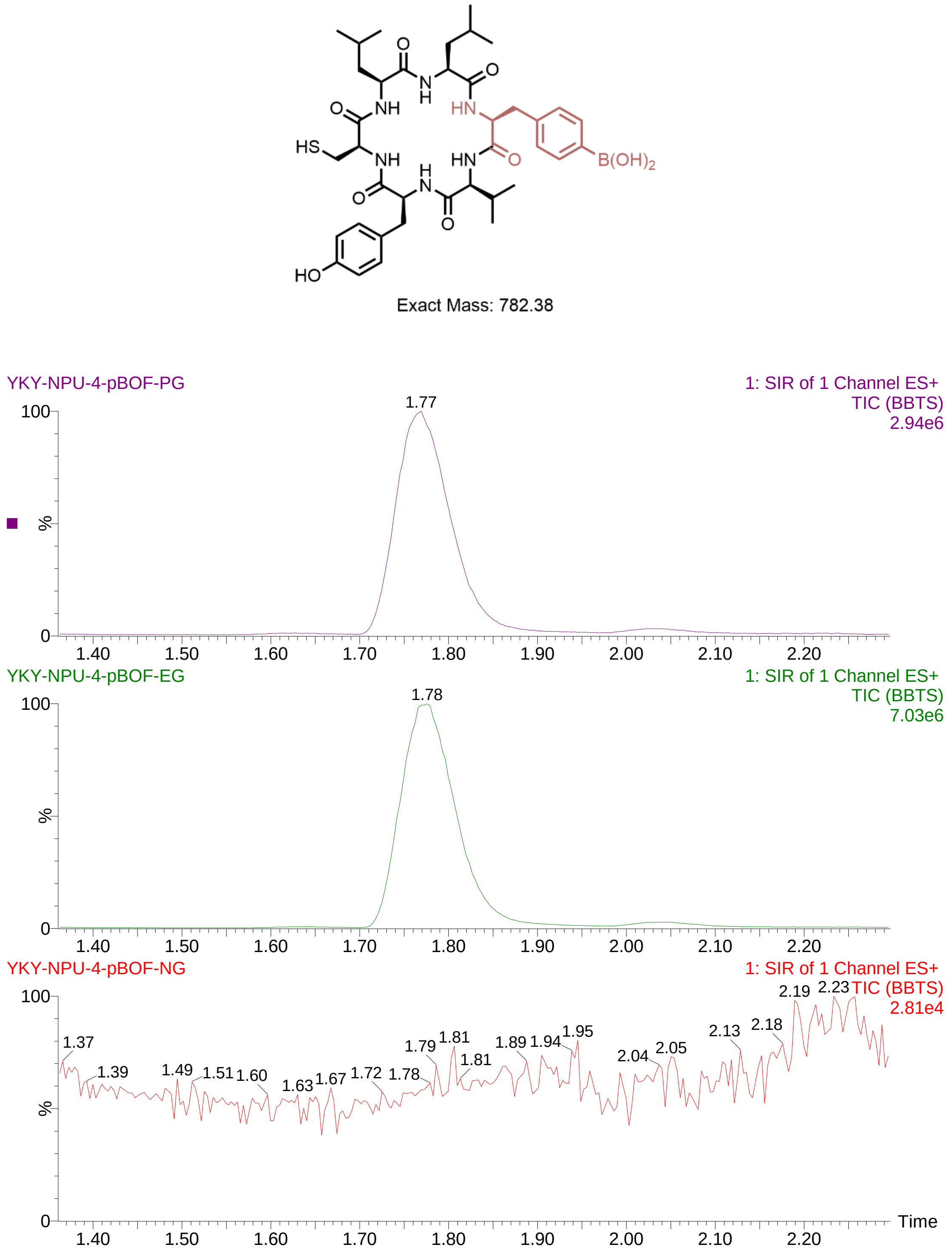

Supplementary Figure 78 Mass Spectrometry (MS) Analysis of cyclo-CLL[TAG]VY containing *p*-boronophenylalanine.

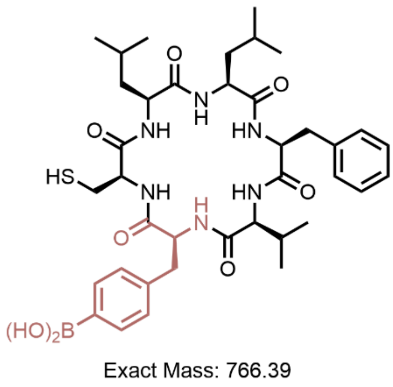

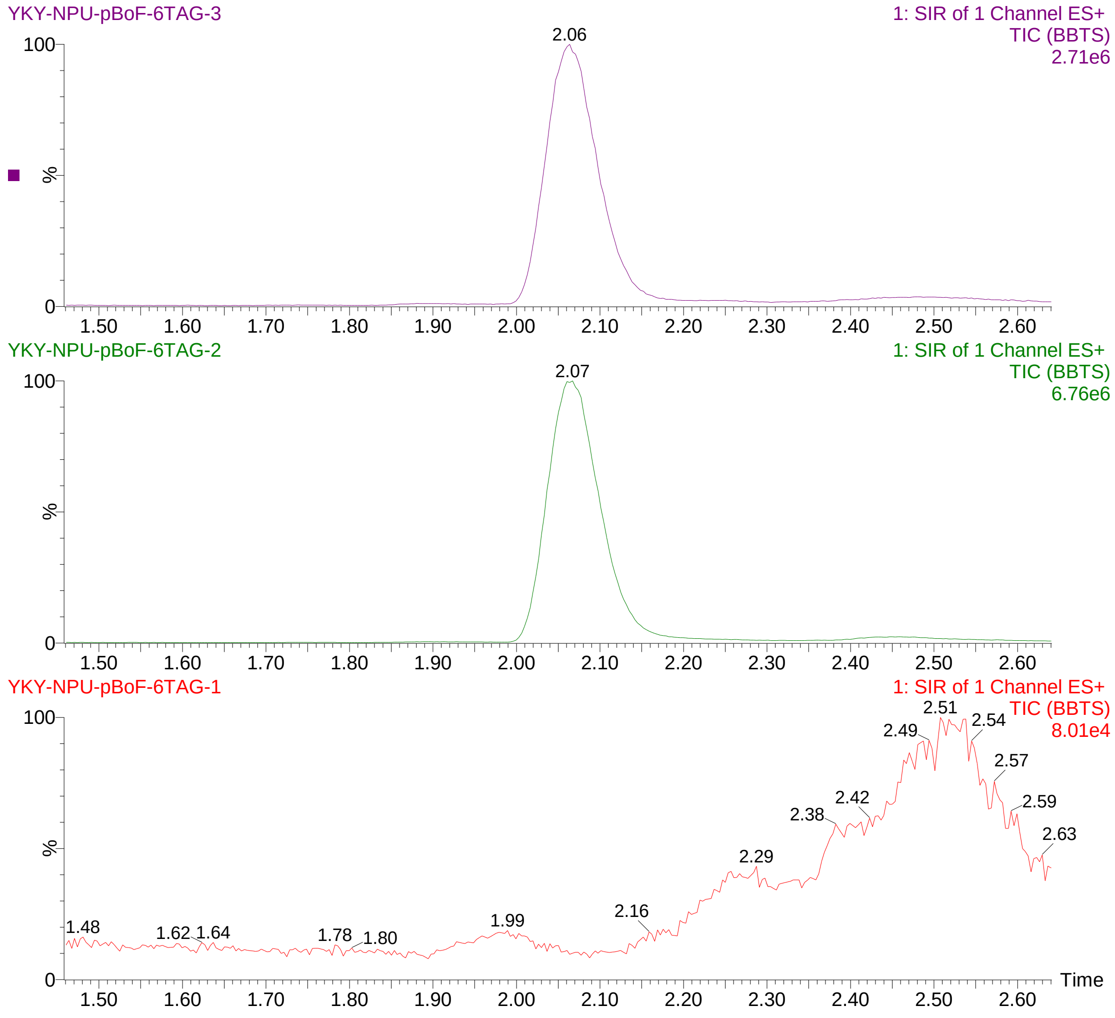

Supplementary Figure 79 Mass Spectrometry (MS) Analysis of cyclo-CLLFV[TAG] containing *p*-boronophenylalanine.

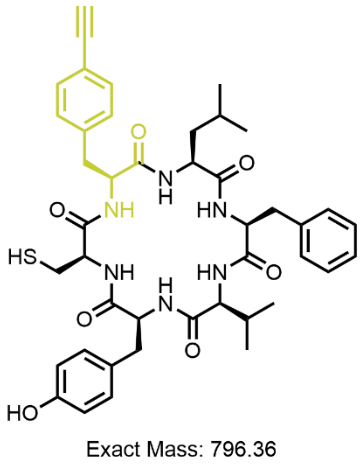

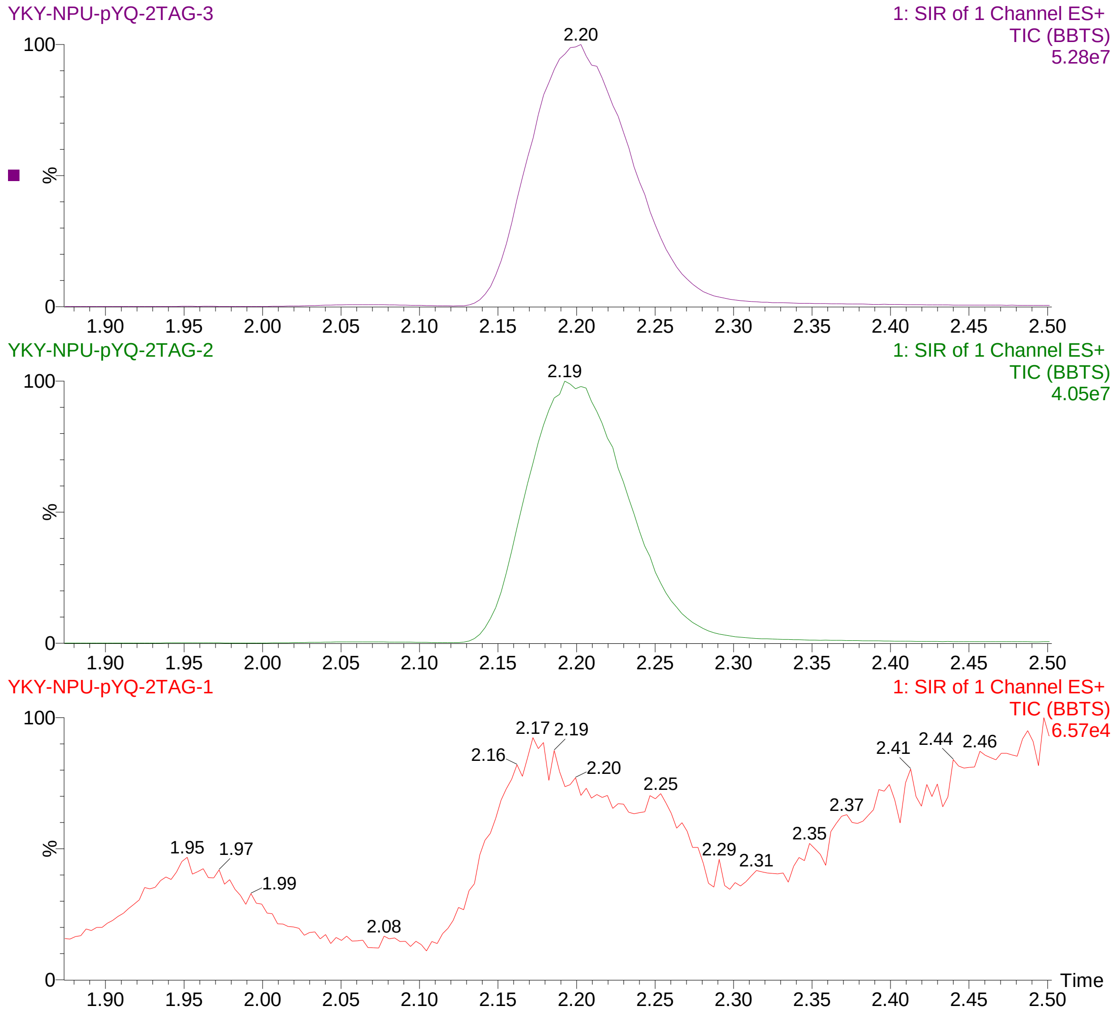

Supplementary Figure 80 Mass Spectrometry (MS) Analysis of cyclo-C[TAG]LFVY containing *p*-ethynylphenylalanine.

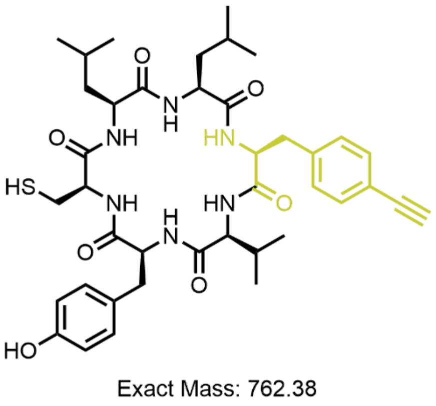

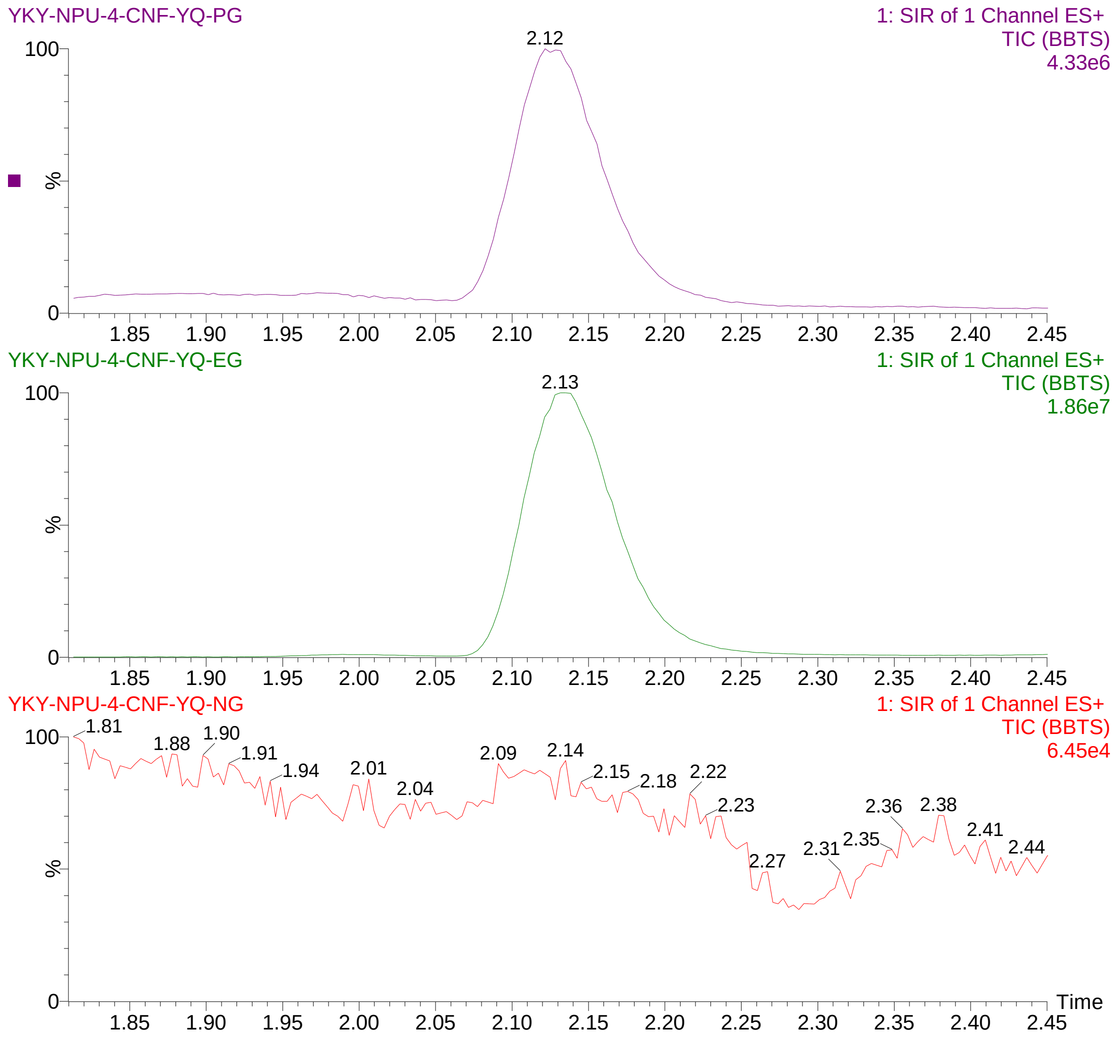

Supplementary Figure 81 Mass Spectrometry (MS) Analysis of cyclo-CLL[TAG]VY containing *p*-ethynylphenylalanine.

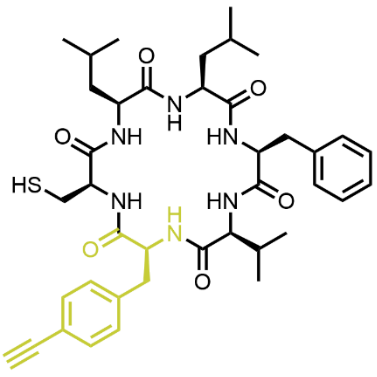

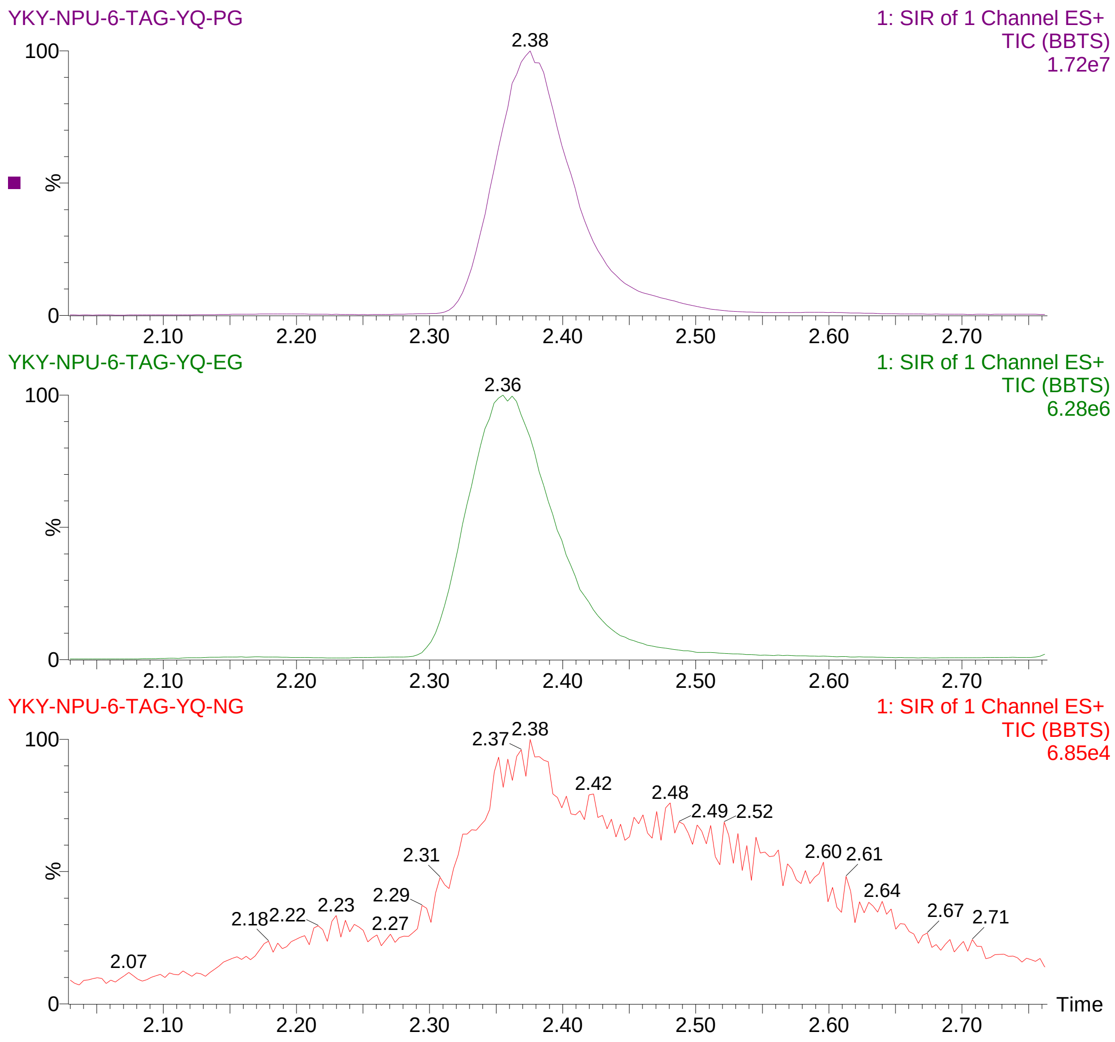

Supplementary Figure 82 Mass Spectrometry (MS) Analysis of cyclo-CLLFV[TAG] containing *p*-ethynylphenylalanine.

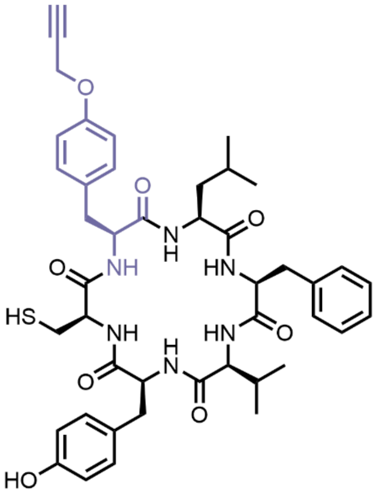

Exact Mass: 826.37

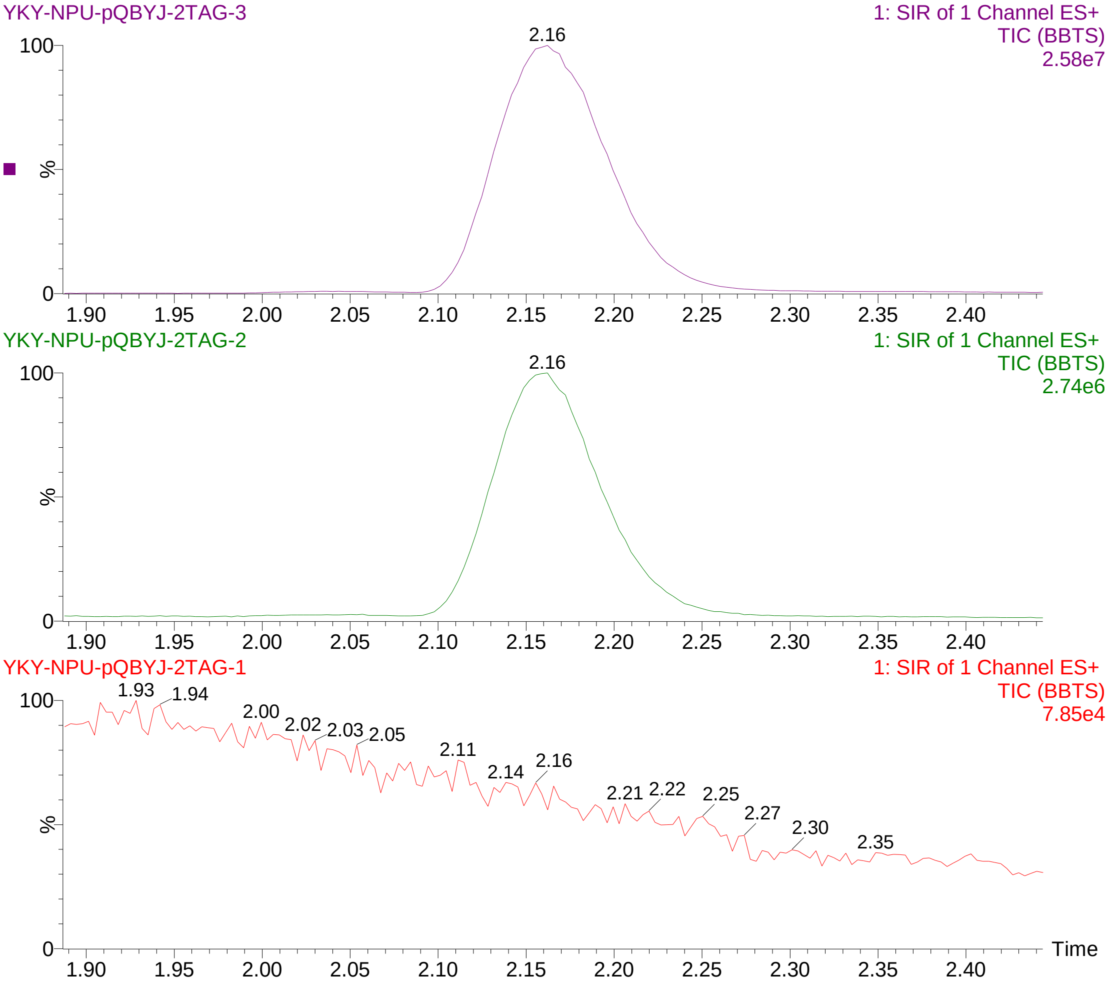

Supplementary Figure 83 Mass Spectrometry (MS) Analysis of cyclo-C[TAG]LFVY containing *p*-propargyloxyphenylalanine.

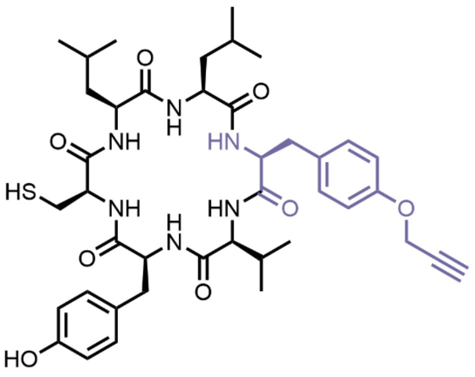

Exact Mass: 792.39

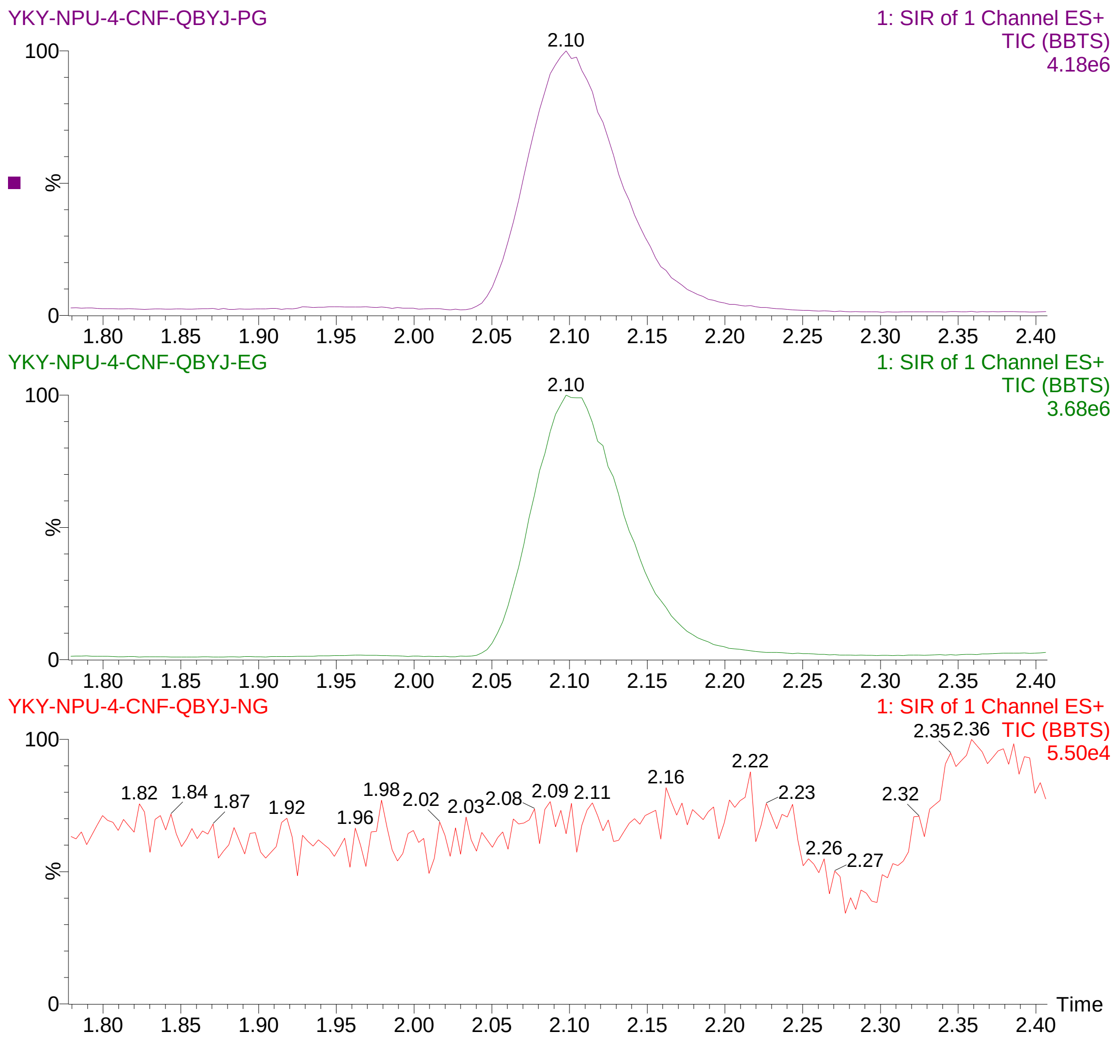

Supplementary Figure 84 Mass Spectrometry (MS) Analysis of cyclo-CLL[TAG]VY containing *p*-propargyloxyphenylalanine.

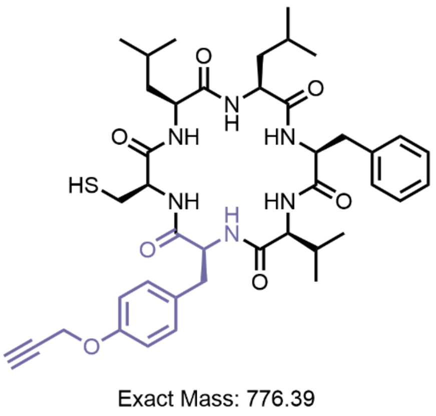

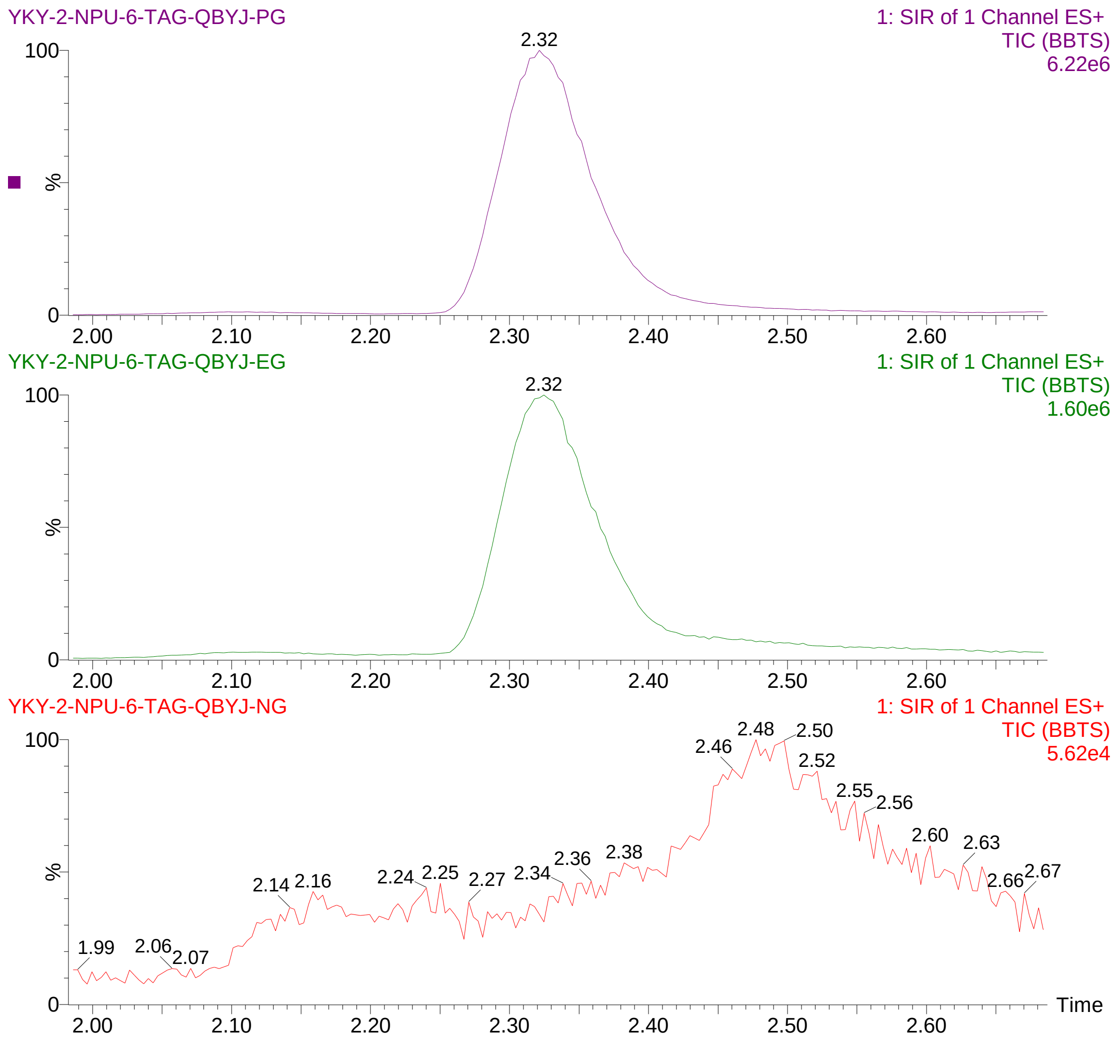

Supplementary Figure 85 Mass Spectrometry (MS) Analysis of cyclo-CLLFV[TAG] containing *p*-propargyloxyphenylalanine.
