# Supplementary material for: A robust platform streamlining aromatic noncanonical amino acid biosynthesis and genetic code expansion in Escherichia coli
Source: Nat Commun. 2025 Sep 29;16:8605. doi: 10.1038/s41467-025-63679-6 (PMC12480666; doi:10.1038/s41467-025-63679-6)
Supplement: Supplementary file 3 — Description of Additional Supplementary Files [file 41467_2025_63679_MOESM3_ESM.pdf]

Description Of Additional Supplementary Files

File name: Supplementary Data 1
Description: all the amino acid and nucleotide sequences in this work.

File name: Supplementary Data 2
Description: all the primers sequences used for plasmid construction in this work.
