# Supplementary material for: A robust platform streamlining aromatic noncanonical amino acid biosynthesis and genetic code expansion in Escherichia coli
Source: Nat Commun. 2025 Sep 29;16:8605. doi: 10.1038/s41467-025-63679-6 (PMC12480666; doi:10.1038/s41467-025-63679-6)
Supplement: Supplementary file 4 — Supplementary Data 1 [file 41467_2025_63679_MOESM4_ESM.pdf]

Supplementary data 1

Protein and DNA sequences

PpLTA:

MNGETSRPPALGFSSDNIAGASPEVAQALVKHSSGQAGPYGTDELTAQVKRKFCEIFE
RDVEVFLVPTGTAANALCLSAMTPPWGNIYCHPASHINNDECGAPEFFSNGAKLMTV
DGPAAKLDIVRLRERTREKVGDVHTTQPACVSITQATEVGSIYTLDEIEAIGDVCKSSS
LGLHMDGSRFANALVSLGCSPAEMTWKAGVDALSFGATKNGVLAAEAIVLFNTSLAT
EMSYRRKRAGHLSSKMRFLSAQIDAYLTDDLWLRNARKANAAAQRLAQGLEGLGG
VEVLGGTEANILFCRLDSAMIDALLKAGFGFYHDRWGPNVVRFVTSFATTAEDVDHL
LNQVRLAADRTQER

ATGAACGGCGAAACGAGCCGCCCGCCGGCGCTGGGCTTTAGCAGCGATAACATTG
CGGGCGCGAGCCCGGAAGTGGCGCAAGCGCTGGTGAAACATAGCAGCGGCCAAG
CGGGCCCGTATGGCACCGATGAACTGACCGCGCAAGTGAAACGCAAATTTTGCGA
AATTTTTGAACGCGATGTGGAAGTGTTTCTGGTGCCGACCGGCACCGCGGCCAAC
GCGCTGTGCCTGAGCGCGATGACCCCGCCGTGGGGCAACATTTATTGCCATCCGGC
GAGCCATATTAACAACGATGAATGCGGCGCGCCGGAATTTTTTAGCAACGGCGCGA
AACTGATGACCGTGGATGGCCCGGCGGCGAAACTGGATATTGTGCGCCTGCGCGA
ACGCACCCGCGAAAAAGTGGGCGATGTGCATACCACGCAGCCGGCGTGCGTGAG
CATTACCCAAGCGACCGAAGTGGGCAGCATTTATACCCTGGATGAAATTGAAGCGA
TTGGCGATGTGTGCAAAAGCAGTAGCCTGGGCCTGCACATGGATGGCAGCCGCTT
TGCGAACGCGTTAGTGAGCCTGGGCTGCAGCCCGGCGGAAATGACCTGGAAAGC
GGGCGTGGATGCGCTGAGCTTTGGCGCGACCAAAAACGGCGTGCTGGCGGCGGA
AGCGATTGTGCTGTTTAACACGAGCCTGGCGACCGAAATGAGCTATCGCCGCAAA
CGCGCGGGCCATCTGAGCAGCAAAATGCGCTTTCTGAGCGCGCAGATTGATGCGT
ATCTGACCGACGATCTGTGGTTACGCAACGCGCGCAAGGCGAATGCCGCGGCGCA
GCGCTTAGCGCAAGGCCTGGAAGGCTTAGGTGGCGTGGAAGTGCTGGGCGGCAC
CGAAGCGAACATTCTGTTTTGCCGCCTGGATAGCGCGATGATTGATGCGCTGCTGA
AAGCGGGCTTTGGCTTTTATCATGATCGCTGGGGCCCGAACGTGGTGCGCTTTGTG
ACGAGCTTTGCGACCACCGCGGAAGATGTGGATCATCTGCTGAACCAAGTGCGCC
TGGCGGCGGATCGCACCCAAGAACGCTAA

LmLTA:

MSTPRTTATAAKPKPYSFVNDYSVGMHPKILDLMARDNMTQHAGYGQDSHCAKAA
RLIGELLERPDADVHFISGGTQTNLIACSLALRPWEAVIATQLGHISTHETGAIEATGHK
VVTAPCPDGKLRVADIESALHENRSEHMVIPKLVYISNTTEVGTQYTKQELEDISASCK
EHGLYLFLDGARLASALSSPVNDLTLADIARLTDMFYIGATKAGGMFGEALIILNDAL
KPNARHLIKQRGALMAKGWLLGIQFEVLMKDNLFFELGAHSNKMAAILKAGLEACG
IRLAWPSASNQLFPILENTMIAELNNDFDMYTVEPLKDGTCIMRLCTSWATEEKECHR
FVEVLKRLVASTA

ATGAGCACCCCGCGCACCACCGCGACCGCGGCGAAACCGAAACCGTATAGCTTTG
TGAACGATTATAGCGTGGGCATGCATCCGAAAATTCTGGATCTGATGGCGCGCGAT
AACATGACGCAGCATGCGGGCTATGGCCAAGATAGCCATTGCGCGAAAGCGGCGC
GCCTGATTGGCGAACTGCTGGAACGCCCGGATGCGGATGTGCATTTTATTAGCGGC
GGCACGCAGACCAACCTGATTGCGTGCAGCCTGGCGCTGCGCCCGTGGGAAGCG

GTGATTGCGACGCAGCTGGGCCATATTAGCACCCATGAAACCGGCGCGATTGAAG
CGACCGGCCATAAAGTGGTGACCGCGCCGTGCCCGGATGGCAAACTGCGCGTGGC
GGATATTGAAAGTGCGCTGCATGAAAACCGCAGCGAACACATGGTGATTCCGAAA
CTGGTGTATATTAGCAACACCACCGAAGTGGGCACGCAGTATACCAAACAAGAAC
TGGAAGATATTAGCGCGAGCTGCAAAGAACATGGCCTGTATCTGTTTCTGGATGGC
GCGCGCCTGGCGAGCGCGCTGAGCAGCCCGGTGAACGATCTGACCCTGGCGGAC
ATTGCGCGCCTGACCGATATGTTTTATATTGGCGCGACCAAAGCGGGCGGCATGTT
TGGCGAAGCGCTGATTATTCTGAACGATGCGCTGAAACCGAACGCGCGCCATCTG
ATTAAACAGCGCGGCGCGCTGATGGCGAAAGGCTGGCTGCTGGGCATTCAGTTTG
AAGTGCTGATGAAAGATAACCTGTTTTTTGAACTGGGCGCGCATAGCAACAAAAT
GGCGGCGATTCTGAAAGCGGGCCTGGAAGCGTGCGGCATTCGCCTGGCGTGGCCG
AGCGCGAGCAATCAGCTGTTTCCGATTCTGGAAAACACCATGATTGCGGAACTGA
ACAACGATTTTGATATGTATACCGTGGAACCGCTGAAAGATGGCACCTGCATTATGC
GCCTGTGCACGAGCTGGGCGACCGAAGAAAAAGAATGCCATCGCTTTGTGGAAGT
GCTGAAACGCCTGGTGGCGAGCACCGCGTAA

NmLTA:

MASNDSCIEDTVSFTSDNIAAAAPEIVQAMAQACQGNAQPYGGDALTQNVEAQLKAI
FECDLQLFLVPTGSAANAISLAALTPPWGAILCHQESHINNDECGAPEFFTAGAKLIAV
AGTHGKLDPQALTQAARNKRGDVHSVEPTTVSITQATEVGSIYALDELNEIGQICRNE
GLKLHMDGARFANALSALGCTPAEMTWKAGVDVLSFGATKNGSLCAEAIILFDKSYA
QEIAFRRKRGGHLLSKMRFLSAQMHAYLADDLWLTNARHANLMAARLAAGLSALSR
VSLIAPTESNIIFCRMPTKMIAALQQQGFQFYHDRWGDGIVRLVTSFATTQAQVDTFIA
AAAQLNQNTD

ATGGCGAGCAACGATAGCTGCATTGAAGATACCGTGAGCTTTACGAGCGATAACAT
TGCGGCCGCGGCGCCGGAAATTGTGCAAGCGATGGCGCAAGCGTGCCAAGGCAA
CGCGCAGCCGTATGGCGGCGATGCGCTGACCCAAAACGTGGAAGCGCAGCTGAA
AGCGATTTTTGAATGCGATCTGCAGCTGTTTCTGGTTCCGACCGGCAGCGCGGCGA
ACGCCATTAGCTTAGCGGCGCTGACCCCGCCGTGGGGCGCGATTCTGTGCCATCAA
GAAAGCCATATTAACAACGATGAATGCGGCGCGCCGGAATTTTTTACCGCGGGCGC
GAAACTGATTGCGGTGGCGGGCACCCATGGCAAACTGGATCCGCAAGCGCTGACC
CAAGCGGCGCGCAACAAACGCGGCGATGTGCATAGCGTGGAACCGACCACCGTG
AGCATTACCCAAGCGACCGAAGTGGGCAGCATTTATGCGCTGGATGAACTGAACG
AAATTGGTCAGATTTGCCGCAACGAAGGCCTGAAACTGCACATGGATGGCGCGCG
CTTTGCGAACGCGCTGAGCGCCTTAGGCTGTACCCCGGCGGAAATGACCTGGAAA
GCGGGCGTGGATGTGCTGAGCTTTGGCGCGACCAAAAACGGCAGCCTGTGCGCG
GAAGCGATTATTCTGTTTGATAAAAGCTATGCGCAAGAAATTGCGTTTCGCCGCAA
ACGCGGCGGCCATCTGCTGAGCAAAATGCGCTTTCTGAGCGCGCAGATGCATGCG
TATCTGGCGGATGATCTGTGGCTGACGAATGCCCGCCACGCGAACCTGATGGCCGC
CCGCCTGGCCGCCGGCTTAAGTGCGCTGAGTCGCGTGAGCCTGATTGCGCCGACC
GAAAGCAACATTATTTTTTGCCGCATGCCGACCAAAATGATTGCGGCGCTGCAGCA
ACAAGGCTTTCAGTTTTATCATGATCGCTGGGGCGATGGCATTGTGCGCCTGGTGA
CGAGCTTTGCGACCACCCAAGCGCAAGTGGATACCTTTATTGCGGCCGCGGCGCA
GCTGAATCAGAACACCGATTAA

CsLTA:

MYSFKNDYSEGAHPKILEALIASNLEQTEGYGEDHYSQKAAWLLKEMIGRDDIAVHF
FVGGTQTNLTAISAFLRPHQAVIAAATGHIATHETGAIEATGHKVITVETSDGKLRMDH
IQSVLDGHTDEHMVSPKMVYISNSTEVGSIYKKAELEGLSQFCKANNLLLYLDGARL
GSALTSKENDMTLLDLGRLTDVFYIGGTKNGALMGEALIICNDFLKEDFRFHIKQKGA
LLAKGRLLGIQFEALFKDNLYFELAEHANQMAVRLQDEIKKLGFSFLISSPSNQVFPIFP
NSVIEKLQEKYAFHIWEKVDDSYSAIRLVTSWATKEEAVSNFVKDLNNIVF
ATGTATAGCTTTAAAAACGATTATAGCGAAGGCGCGCATCCGAAAATTCTGGAAGC
GCTGATTGCGAGCAACCTGGAACAGACCGAAGGCTATGGCGAAGATCATTATAGT
CAGAAAGCGGCGTGGCTGCTGAAAGAAATGATTGGCCGCGATGATATTGCGGTGC
ATTTTTTTGTGGGCGGCACGCAGACCAACCTGACCGCGATTAGCGCGTTTCTGCGC
CCGCATCAAGCGGTGATTGCGGCGGCCACCGGCCATATTGCGACCCATGAAACCG
GCGCGATTGAAGCGACCGGCCACAAAGTTATTACCGTGGAAACGAGCGATGGCAA
ACTGCGCATGGATCATATTCAGAGCGTGCTGGATGGCCATACCGATGAACACATGG
TGAGCCCGAAAATGGTGTATATTAGCAACAGCACCGAAGTGGGCAGCATTTATAAA
AAAGCGGAACTGGAAGGCCTGAGTCAGTTTTGCAAAGCGAACAACCTGTTACTGT
ATCTGGATGGCGCGCGCCTGGGCAGCGCGCTGACGAGCAAAGAAAACGATATGAC
CCTGCTGGATCTGGGCCGCCTGACCGATGTGTTTTATATTGGCGGCACCAAAAACG
GCGCGCTGATGGGCGAGGCGCTGATTATTTGCAACGATTTTCTGAAAGAAGATTTT
CGCTTTCATATTAAACAGAAAGGCGCGCTGCTGGCGAAAGGCCGCCTGCTGGGCA
TTCAGTTTGAAGCGCTGTTTAAAGATAACCTGTATTTTGAACTGGCGGAACATGCG
AATCAGATGGCGGTGCGCCTGCAAGATGAAATTAAAAAACTGGGCTTTAGCTTTCT
GATTAGCAGCCCGAGCAACCAAGTGTTTCCGATTTTTCCGAACAGCGTGATTGAAA
AACTGCAAGAAAAATATGCGTTTCATATTTGGGAAAAAGTGGATGATAGCTATAGC
GCGATTCGCCTGGTGACGAGCTGGGCGACCAAAGAAGAAGCGGTGAGCAACTTT
GTGAAAGATCTGAACAACATTGTGTTTTAA

RpTD:

MTQLDTTTLPDLSAIAGLRARLKQWVRTTPVFDKTDFEPVPGTAVNFKLELLQASGTF
KARGAFSNLLALDDDQRAAGVTCVSAGNHAVGVAYAAMRLGIPAKVVMIKTASPAR
VALCRQYGAEVVLAENGQTAFDTVHRIESEEGRFFVHPFNGYRTVLGTATLGHEWLE
QAGALDAVIVPIGGGGLMAGVSTAVKLLAPQCQVIGVEPEGADAMHRSFETGGPVK
MGSMQSIADSLMAPHTEQYSYELCRRNVDRLVKVSDDELRAAMRLLFDQLKLATEP
ACATATAALVGGLKAELAGKRVGVLLCGTNTDAATFARHLGLG
ATGACGCAGCTGGATACCACGACCCTGCCGGATCTGAGCGCGATTGCGGGCCTGC
GCGCGCGCCTGAAACAGTGGGTGCGCACCACCCCGGTGTTTGATAAAACCGATTT
TGAACCGGTGCCGGGCACCGCGGTGAACTTCAAACTGGAACTGCTGCAAGCGAG
CGGCACCTTTAAAGCGCGCGGCGCGTTTAGCAACCTGCTGGCGCTGGATGATGAC
CAACGCGCCGCGGGCGTGACGTGCGTGAGCGCGGGCAACCATGCCGTTGGCGTT
GCGTATGCGGCGATGCGCCTGGGCATTCCGGCGAAAGTGGTGATGATTAAAACCG
CGAGCCCGGCGCGCGTGGCGCTGTGCCGTCAGTATGGCGCGGAAGTGGTGCTGGC
GGAAAACGGTCAGACCGCGTTTGATACCGTGCATCGCATTGAAAGCGAAGAAGGC
CGCTTTTTTGTGCATCCGTTTAACGGCTATCGCACCGTGCTGGGCACCGCGACCCT
GGGCCATGAATGGCTGGAACAAGCGGGCGCGCTGGATGCGGTGATTGTGCCGATT
GGCGGTGGCGGCCTGATGGCGGGCGTGAGTACCGCGGTGAAACTGCTGGCGCCG
CAGTGCCAAGTGATTGGCGTGGAACCGGAAGGCGCGGATGCGATGCATCGCAGCT

TTGAAACCGGCGGCCCGGTGAAAATGGGCAGCATGCAGAGCATTGCGGATAGCCT
GATGGCGCCGCATACCGAACAGTATAGCTATGAACTGTGCCGCCGCAACGTGGATC
GCCTGGTGAAAGTGAGCGATGATGAACTGCGCGCGGCGATGCGTCTGCTGTTCGA
TCAGTTAAAACTGGCGACGGAACCGGCGTGCGCGACGGCGACCGCGGCGTTAGT
GGGCGGCCTGAAAGCGGAACTGGCGGGCAAACGCGTGGGCGTGCTGCTGTGCGG
CACCAACACCGATGCGGCGACCTTTGCGCGCCATCTGGGCCTGGGCTAA

sfGFP:

MSKGEELFTGVVPILVELDGDVNGHKFSVRGEGEGDATNGKLTLKFICTTGKLPVPW
PTLVTTLTYGVQCFSRYPDHMKRHDFFKSAMPEGYVQERTISFKDDGTYKTRAEVKF
EGDTLVNRIELKGIDFKEDGNILGHKLEYNFNSHNVYITADKQKNGIKANFKIRHNVE
DGSVQLADHYQQNTPIGDGPVLLPDNHYLSTQSVLSKDPNEKRDHMVLLEFVTAAGI
THGMDELYKLLEHHHHHH

ATGAGCAAAGGCGAAGAACTGTTTACCGGCGTGGTGCCGATTCTGGTGGAACTGG
ATGGCGATGTGAACGGCCATAAATTTAGCGTGCGCGGCGAAGGCGAAGGCGATGC
GACCAACGGCAAACTGACCCTGAAATTTATTTGCACCACCGGCAAACTGCCGGTG
CCGTGGCCGACCCTGGTGACCACCCTGACCTATGGCGTGCAGTGCTTTAGCCGCTA
TCCGGATCACATGAAACGCCATGATTTTTTTAAAAGCGCGATGCCGGAAGGCTATG
TGCAAGAACGCACCATTAGCTTTAAAGATGATGGCACCTATAAAACCCGCGCGGA
AGTGAAATTTGAAGGCGATACCCTGGTGAACCGCATTGAACTGAAAGGCATTGATT
TTAAAGAAGATGGCAACATTCTGGGCCATAAACTGGAATATAACTTTAACAGCCAT
AACGTGTATATTACCGCGGATAAACAGAAAAACGGCATTAAAGCGAACTTTAAAAT
TCGCCATAACGTGGAAGATGGCAGCGTGCAGCTGGCGGATCATTATCAGCAGAAC
ACCCCGATTGGCGATGGCCCGGTGCTGCTGCCGGATAACCATTATCTGAGCACGCA
GAGCGTGCTGAGCAAAGATCCGAACGAAAAACGCGATCACATGGTGCTGCTGGAA
TTTGTGACCGCGGCGGGCATTACCCATGGCATGGATGAACTGTATAAACTGCTCGA
GCACCACCACCACCACCACTGA

Her2-scFV:

MKKNIAFLLASMFVFSIATNAYADIQMTQSPSSLSASVGDRVTITCRASQDVNTAVAW
YQQKPGKAPKLLIYSASFLYSGVPSRFSGSRSGTDFTLTISSLQPEDFATYYCQQHYTTP
PTFGQGTKLEIKRTGSTSGSGKPGSGEGSEVQLVESGGGLVQPGGSLRLSCAASGFNIK
DTYIHWVRQAPGKGLEWVARIYPTNGYTRYADSVKGRFTISADTSKNTAYLQMNSLR
AEDTAVYYCSRWGGDGFYAMDYWGQGTLVTVSSLEHHHHHH

ATGAAAAAAAACATTGCGTTTCTGCTGGCGAGCATGTTTGTGTTTAGCATTGCGAC
CAACGCGTATGCGGATATTCAGATGACGCAGAGCCCGAGCAGCCTGAGCGCGAGC
GTGGGCGATCGCGTGACCATTACCTGCCGCGCGAGCCAAGATGTGAACACCGCGG
TGGCGTGGTATCAGCAGAAACCGGGCAAAGCGCCGAAACTGCTGATTTATAGCGC
GAGCTTTCTGTATAGCGGCGTGCCGAGCCGCTTTAGCGGCAGCCGCAGCGGCACC
GATTTTACCCTGACCATTAGCAGCCTGCAGCCGGAAGATTTTGCGACCTATTATTGT
CAGCAGCATTATACCACCCCGCCGACCTTTGGCCAAGGCACGAAACTGGAAATTA
AACGCACCGGCAGCACGAGCGGCAGCGGCAAACCGGGCAGCGGCGAAGGCAGC
GAGGTGCAATTAGTTGAAAGCGGCGGTGGCCTGGTGCAGCCGGGCGGCAGCCTG
CGCCTGAGCTGCGCGGCGAGCGGCTTTAACATTAAAGATACCTATATTCATTGGGT
GCGCCAAGCGCCGGGCAAAGGCCTGGAATGGGTGGCGCGCATTTATCCGACCAAC
GGCTATACCCGCTATGCGGATAGCGTGAAAGGCCGCTTTACCATTAGCGCGGATAC

GAGCAAAAACACCGCGTATCTGCAGATGAACAGCCTGCGCGCGGAAGATACCGCG
GTGTATTATTGCAGCCGCTGGGGCGGCGATGGCTTTTATGCGATGGATTATTGGGGC
CAAGGCACCCTGGTGACCGTGAGCAGCCTCGAGCACCACCACCACCACCACTGA

Her2-Fab-light chain:

MKKNIAFLLASMFVFSIATNAYADIQMTQSPSSLSASVGDRVTITCRASQDVNTAVAW
YQQKPGKAPKLLIYSASFLYSGVPSRFSGSRSGTDFTLTISSLQPEDFATYYCQQHYTTP
PTFGQGTKLEIKRTVAAPSVFIFPPSDEQLKSGTASVVCLLNNFYPREAKVQWKVDNA
LQSGNSQESVTEQDSKDSTYSLSSTLTLSKADYEKHKVYACEVTHQGLSSPVTKSFNR
GEC

ATGAAAAAGAATATCGCATTTCTTCTTGCTAGCATGTTCGTTTTTTCTATTGCTACAA
ACGCATACGCTGACATCCAGATGACCCAGTCTCCATCCTCCCTGTCTGCATCTGTAG
GAGACAGAGTCACCATCACTTGCCGGGCAAGTCAGGATGTGAATACCGCGGTCGC
ATGGTATCAGCAGAAACCAGGGAAAGCCCCTAAGCTCCTGATCTATTCTGCATCCT
TCTTGTATAGTGGGGTCCCATCAAGGTTCAGTGGCAGTAGATCTGGGACAGATTTC
ACTCTCACCATCAGCAGTCTGCAACCTGAAGATTTTGCAACTTACTACTGTCAACA
GCATTACACTACCCCTCCGACGTTCGGCCAAGGTACCAAGCTTGAGATCAAACGA
ACTGTGGCTGCACCATCTGTCTTCATCTTCCCGCCATCTGATGAGCAGTTGAAATCT
GGAACTGCCTCTGTCGTGTGCCTGCTGAATAACTTCTATCCCAGAGAGGCCAAAGT
ACAGTGGAAGGTGGATAACGCCCTCCAATCGGGTAACTCCCAGGAGAGTGTCACA
GAGCAGGACAGCAAGGACAGCACCTACAGCCTCAGCAGCACCCTGACGCTGAGC
AAAGCAGACTACGAGAAACACAAAGTCTACGCCTGCGAAGTCACCCATCAGGGC
CTGTCCTCGCCCGTCACAAAGAGCTTCAACAGGGGAGAGTGTTAA

Her2-Fab-heavy chain:

MKKNIAFLLASMFVFSIATNAYAEVQLVESGGGLVQPGGSLRLSCAASGFNIKDTYIH
WVRQAPGKGLEWVARIYPTNGYTRYADSVKGRFTISADTSKNTAYLQMNSLRAEDTA
VYYCSRWGGDGFYAMDYWGQGTLVTVSSASTKGPSVFPLAPSSKSTSGGTAALGCL
VKDYFPEPVTVSWNSGALTSGVHTFPAVLQSSGLYSLSSVVTVPSSSLGTQTYICNVN
HKPSNTKVDKKVEPKSCDKTHTAAALEHHHHHH

ATGAAAAAGAATATCGCATTTCTTCTTGCATCTATGTTCGTTTTTTCTATTGCTACAA
ACGCGTACGCTGAGGTGCAGCTGGTGGAGTCTGGAGGAGGCTTGGTCCAGCCTGG
GGGGTCCCTGAGACTCTCCTGTGCAGCCTCTGGGTTCAATATTAAGGACACTTACA
TCCACTGGGTCCGCCAGGCTCCAGGGAAGGGGCTGGAGTGGGTCGCACGTATTTA
TCCTACCAATGGTTACACACGCTACGCAGACTCCGTGAAGGGCCGATTCACCATCT
CCGCAGACACTTCCAAGAACACGGCGTATCTTCAAATGAACAGCCTGAGAGCCGA
GGACACGGCCGTGTATTACTGTTCGAGATGGGGCGGTGACGGCTTCTATGCCATGG
ACTACTGGGGCCAAGGAACCCTGGTCACCGTCTCCTCAGCCTCCACCAAGGGCCC
ATCGGTCTTCCCCCTGGCACCCTCCTCCAAGAGCACCTCTGGGGGCACAGCGGCC
CTGGGCTGCCTGGTCAAGGACTACTTCCCCGAACCGGTGACGGTGTCGTGGAACT
CAGGCGCCCTGACCAGCGGCGTGCACACCTTCCCGGCTGTCCTACAGTCCTCAGG
ACTCTACTCCCTCAGCAGCGTGGTGACTGTGCCCTCTAGCAGCTTGGGCACCCAG
ACCTACATCTGCAACGTGAATCACAAGCCCAGCAACACCAAGGTGGACAAGAAA
GTTGAGCCCAAATCTTGTGACAAAACTCACACAGCGGCCGCACTCGAGCACCACC
ACCACCACCACTGA

J591-Fab-light chain:

MKKNIAFLLASMFVFSIATNAYADIVMTQSPSSLSASVGDRVTITCKASQDVGTAVDW
YQQKPGKAPKLLIYWASTRHTGVPDRFTGSGSGTDFTLTISSLQPEDFADYFCQQYNS
YPLTFGGGTKLEIKRTVAAPSVFIFPPSDEQLKSGTASVVCLLNNFYPREAKVQWKVD
NALQSGNSQESVTEQDSKDSTYSLSSTLTLSKADYEKHKVYACEVTHQGLSSPVTKSF
NRGEC
ATGAAAAAGAATATCGCATTTCTTCTTGCTAGCATGTTCGTTTTTTCTATTGCTACAA
ACGCATACGCTGACATCGTGATGACCCAGTCCCCCTCCTCCCTGTCTGCCTCCGTG
GGCGACAGAGTGACCATCACATGCAAGGCCTCCCAGGATGTGGGCACCGCCGTGG
ACTGGTATCAGCAGAAGCCTGGCAAGGCCCCTAAGCTGCTGATCTACTGGGCCTC
CACCAGACACACCGGCGTGCCTGACAGATTCACCGGCTCCGGCTCTGGCACCGAC
TTCACCCTGACCATCTCCAGCCTGCAGCCTGAGGACTTCGCCGACTACTTCTGCCA
GCAGTACAACTCCTACCCTCTGACCTTCGGCGGAGGCACCAAGCTGGAAATCAAA
CGAACTGTGGCTGCACCATCTGTCTTCATCTTCCCGCCATCTGATGAGCAGTTGAA
ATCTGGAACTGCCTCTGTCGTGTGCCTGCTGAATAACTTCTATCCCAGAGAGGCCA
AAGTACAGTGGAAGGTGGATAACGCCCTCCAATCGGGTAACTCCCAGGAGAGTGT
CACAGAGCAGGACAGCAAGGACAGCACCTACAGCCTCAGCAGCACCCTGACGCT
GAGCAAAGCAGACTACGAGAAACACAAAGTCTACGCCTGCGAAGTCACCCATCA
GGGCCTGTCCTCGCCCGTCACAAAGAGCTTCAACAGGGGAGAGTGTTAA

J591-Fab-heavy chain:

MKKNIAFLLASMFVFSIATNAYAEVQLVQSGAEVKKPGASVKISCKTSGYTFTEYTIH
WVKQASGKGLEWIGNINPNNGGTTYNQKFEDRATLTVDKSTSTAYMELSSLRSEDTA
VYYCAAGWNFDYWGQGTTVTVSSASTKGPSVFPLAPSSKSTSGGTAALGCLVKDYF
PEPVTVSWNSGALTSGVHTFPAVLQSSGLYSLSSVVTVPSSSLGTQTYICNVNHKPSNT
KVDKKVEPKSCDKTHTAAALEHHHHHH
ATGAAAAAGAATATCGCATTTCTTCTTGCATCTATGTTCGTTTTTTCTATTGCTACAA
ACGCGTACGCTGAAGTGCAGCTGGTGCAGTCTGGCGCCGAAGTGAAGAAACCTG
GCGCCTCCGTGAAGATCTCCTGCAAGACCTCCGGCTACACCTTCACCGAGTACAC
CATCCACTGGGTGAAACAGGCCTCCGGCAAGGGCCTGGAATGGATCGGCAACATC
AACCCTAACAACGGCGGCACCACCTACAACCAGAAGTTCGAGGACCGGGCCACC
CTGACCGTGGACAAGTCCACCTCCACCGCCTACATGGAACTGTCCTCCCTGCGGT
CTGAGGACACCGCCGTGTACTACTGCGCCGCTGGCTGGAACTTCGACTACTGGGG
CCAGGGCACCACAGTGACAGTCTCGAGCGCCTCCACCAAGGGCCCATCGGTCTTC
CCCCTGGCACCCTCCTCCAAGAGCACCTCTGGGGGCACAGCGGCCCTGGGCTGCC
TGGTCAAGGACTACTTCCCCGAACCGGTGACGGTGTCGTGGAACTCAGGCGCCCT
GACCAGCGGCGTGCACACCTTCCCGGCTGTCCTACAGTCCTCAGGACTCTACTCCC
TCAGCAGCGTGGTGACTGTGCCCTCTAGCAGCTTGGGCACCCAGACCTACATCTG
CAACGTGAATCACAAGCCCAGCAACACCAAGGTGGACAAGAAAGTTGAGCCCAA
ATCTTGTGACAAAACTCACACAGCGGCCGCACTCGAGCACCACCACCACCACCAC
TGA

Npu:

MIKIATRKYLGKQNVYDIGVERYHNFALKNGFIASNCLLFVYCLSYDTEILTVEYGILP
IGKIVEKRIECTVYSVDNNGNIYTQPVAQWHDRGEQEVFEYCLEDGCLIRATKDHKF
MTVDGQMMPIDEIFERELDLMRVDNLPNGTAANDENYALAA
ATGATCAAAATTGCTACCCGAAAATATCTAGGAAAACAAAACGTATATGACATCGG

TGTCGAACGCTATCACAACTTCGCTCTCAAAAATGGTTTTATCGCTTCTAACTGCCT
GCTCTTTGTCTACTGTCTTTCCTATGACACTGAGATCCTCACAGTCGAATACGGCAT
TTTGCCGATCGGTAAAATTGTGGAAAAGCGTATCGAGTGTACCGTTTACAGCGTAG
ACAATAACGGGAACATATATACCCAACCAGTGGCGCAGTGGCACGATCGTGGCGA
ACAGGAAGTGTTTGAATATTGCCTGGAAGATGGCTGCCTGATTCGCGCCACCAAA
GATCATAAATTCATGACGGTTGATGGTCAGATGATGCCGATCGACGAAATTTTTGAA
CGTGAACTTGACCTGATGAGAGTAGATAACTTACCGAATGGAACCGCCGCAAACG
ATGAAAACTATGCCCTAGCTGCCTAA
