# Supplementary material for: A robust platform streamlining aromatic noncanonical amino acid biosynthesis and genetic code expansion in Escherichia coli
Source: Nat Commun. 2025 Sep 29;16:8605. doi: 10.1038/s41467-025-63679-6 (PMC12480666; doi:10.1038/s41467-025-63679-6)
Supplement: Supplementary file 5 — Supplementary Data 2 [file 41467_2025_63679_MOESM5_ESM.pdf]

Supplementary data 2

Primers used in this study.

Primer	Sequence (5'-3')	Description
PpLTA-F	taataaggagatataccATGAACGGCGAAACGAGCCGCCC	Four LTAs genes were constructed into pACYCDuet-1 plasmid by homologous recombination
PpLTA-R	tcgacttaagcattatgTTAGCGTTCTTGGGTGCGATCCG	
CsLTA-F	taataaggagatataccATGTATAGCTTTAAAAACGATTAT	
CsLTA-R	tcgacttaagcattatgTTAAAACACAATGTTGTTCAG	
NmLTA-F	taataaggagatataccATGGCGAGCAACGATAGCTGCATT	
NmLTA-R	tcgacttaagcattatgTTAATCGGTGTTCTGATTCAGC	
LmLTA-F	taataaggagatataccATGAGCACCCCGCGCACCACC	
LmLTA-R	tcgacttaagcattatgTTACGCGGTGCTCGCCACCAG	
pA-Rp-line-F	CATAATGCTTAAGTCGAACAGA	
pA-Rp-line-R	GGTATATCTCCTTATTAAAGT	
Y151TAG-F	aacgtg**tag**attaccgcGGATAAACAGAAAAACGGCAT	Y151TAG mutant construction
Y151TAG-R	gcggtaat**cta**cacgttATGGCTGTTAAAGTTATATTCC	
pIFRS-F	aagaggagaaattaaccATGGATAAAAAGCCTCTGAACACT	PylRS mutants and $tRNA^{Pyl}_{CUA}$ genes were constructed into pCDF plasmid by homologous recombination
pIFRS-R	agctcagctaattaagcTTACAGGTTAGTAGAAATACCATTG	
PylHRS-F	aagaggagaaattaaccATGGATAAGAAGCCGCTGGAT	
PylHRS-R	agctcagctaattaagcTTACAGGTTGGTAGAGATACCGT	
pCDF-line-F	GCTTAATTAGCTGAGCTTGGAC	
pCDF-line-R	GGTTAATTTCTCCTCTTTAATG	
pAzF/NaRS-F	caaaggaggtgcggccgcATGGACGAGTTCGAAATGATT	MjTyrRS mutants and $tRNA^{Tyr}_{CUA}$ genes were constructed into pUltra plasmid by homologous recombination
pAzF/NaRS-R	cgtttaaacgcggccgcTTACAGACGTTTGCGAATTGG	
pBoF-F	caaaggaggtgcggccgcATGGATGAATTTGAAATGATT	
pBoF-R	cgtttaaacgcggccgcTTACAGGCGTTTGCGAATCGG	
pUltra-line-F	GCGGCCGCGTTTAAACGGTCTC	
pUltra-line-R	GCGGCCGCACCTCCTTTGTGA	
Her2-sc-S9-F	cagagcccg**tag**agccTGAGCGCGAGCGTGGGCGAT	Antibody fragments TAG mutants construction
Her2-sc-S9-R	ggct**cta**cgggctctgCGTCATCTGAATATCCGCAT	
Her2-sc-K42-F	cgggc**tag**gcgccgaaaCTGCTGATTTATAGCGCGAG	
Her2-sc-K42-R	tttcggcgc**cta**gcccgGTTTCTGCTGATACCACGCCA	
Her2-A121-F	ctcctca**tag**tccaccaAGGGCCCATCGGTCT	
Her2-A121-R	tggtgga**cta**tgaggagACGGTGACCAGGGTT	
J591-A116-F	tcgagc**tag**tccaccaagGGCCCATCGGTCTT	

J591-A116-F	cttggtgga**cta**gctcgaGACTGTCACTGTGGT	
2-TAG-F	taactgc**tag**ctctttgtCTACTGTCTTTCCTA	Npu mutant construction
2-TAG-R	acaaagag**cta**gcagttaGAAGCGATAAAACCATTT	
4-TAG-F	cctgctc**tag**gtctactGTCTTTCCTATGACACTGAGA	
4-TAG-R	agtagac**cta**gagcaggCAGTTAGAAGCGATAA	
6-TAG-F	ctttgtc**tag**tgtcttTCCTATGACACTGAGATCCT	
6-TAG-R	aagaca**cta**gacaaagAGCAGGCAGTTAGAAGCGAT	

Lowercase letters indicated homologous arms and TAG mutation in genes was shown in bold.
